# A nutrition programme using positive deviance approach to reduce undernutrition among urban poor children under-five in Malaysia: A cluster randomised controlled trial protocol

**DOI:** 10.1371/journal.pone.0275357

**Published:** 2022-10-13

**Authors:** Lok Poh Chek, Wan Ying Gan, Yit Siew Chin, Norhasmah Sulaiman

**Affiliations:** 1 Department of Nutrition, Faculty of Medicine and Health Sciences, Universiti Putra Malaysia, Serdang, Selangor, Malaysia; 2 Research Centre of Excellence, Nutrition and Non-Communicable Diseases, Faculty of Medicine and Health Sciences, Universiti Putra Malaysia, Serdang, Selangor, Malaysia; Public Library of Science, UNITED KINGDOM

## Abstract

**Background:**

Childhood undernutrition remains a public health issue that can lead to unfavourable effects in later life. These effects tend to be more devastating among urban poor young children, especially in light of the recent COVID-19 pandemic. There is an immediate need to introduce interventions to reduce childhood undernutrition. This paper described the study protocol of a nutrition programme that was developed based on the positive deviance approach and the evaluation of the effectiveness of the programme among urban poor children aged 3 to 5 years old.

**Methods:**

This mixed-method study will be conducted in two phases at low-cost flats in Kuala Lumpur. Phase one will involve a focus group discussion with semi-structured interviews to explore maternal feeding practices and the types of food fed to the children. Phase two will involve a two-armed cluster randomised controlled trial to evaluate the effectiveness of a programme developed based on the positive deviance approach. The programme will consist of educational lessons with peer-led cooking demonstrations, rehabilitation, and growth monitoring sessions. Intervention group will participate in the programme conducted by the researcher for three months whereas the comparison group will only receive all the education materials and menus used in the programme after data collection has been completed. For both groups, data including height, weight, and dietary intake of children as well as the nutritional knowledge and food security status of mothers will be collected at baseline, immediate post-intervention, and 3-month post-intervention.

**Expected results:**

The positive deviance approach helps to recognise the common feeding practices and the local wisdom unique to the urban poor population. Through this programme, mothers may learn from and be empowered by their peers to adopt new feeding behaviours so that their children can achieve healthy weight gain.

**Trial registration:**

This study was registered with clinicaltrials.gov: NCT04688515 on 29 December 2020, https://www.clinicaltrials.gov/ct2/show/NCT04688515.

## Introduction

Child undernutrition can manifest in three main forms, namely underweight, stunting, and wasting. Globally, 149.2 million children under-five (22.0%) were stunted while another 45.4 million children (6.7%) were wasted in 2020 [1]. The estimated global prevalence of underweight was also high at 12.6% in 2020 [2]. In Malaysia, the prevalence of stunting is showing an increasing trend from 20.7% in 2016 [3] to 21.8% in 2019 [4]. Likewise, the same rising trend was also observed in the prevalence of underweight children whereby it increased from 13.7% in 2016 [3] to 14.1% in 2019 [4]. On the other hand, the prevalence of wasting decreased by 2.1% from 11.5% in 2016 [3] to 9.4% in 2019 [4]. Although the prevalence of wasting was declining, the latest national prevalence of undernutrition among children under-five continued to be high. With the alarming trend of persistent undernutrition among children in Malaysia, there is an immediate need to introduce effective strategies to mitigate this national problem.

In view of the COVID-19 pandemic, urban poor populations, particularly children, are becoming even more susceptible to malnutrition. Significant deterioration in nutritional status among them is likely compared to before. Prior to the pandemic, the low-income population already suffered immensely because they reside in high-cost urban areas. In the aftermath of socioeconomic crisis due to the Movement Control Order (MCO) implemented in the country to control the spread of COVID-19, the urban poor population faces even more hardship and struggle to survive [5]. Young children living in urban poor families are likely exposed to an even higher risk of undernutrition because financially-stricken parents might cut down on meal frequency and diet quality to cope with the economic hardship following the pandemic. In view of this, it is vital to further strengthen the intervention programme and to scale up any actions needed to address the worsening issue of undernutrition among children.

Intervention studies aimed at reducing undernutrition among Malaysian children under-five are still lacking. Most local intervention studies focused on childhood obesity among school children [6–11], or undernutrition among indigenous children [12]. However, similar issues among urban poor children are rarely addressed, perhaps due to the perception that residents in urban areas are likely to be wealthier with better living standards. Besides that, nutritional interventions targeting undernourished children are usually implemented in traditional methods such as the provision of supplemental foods [13–15], growth monitoring [16, 17], and micronutrient supplementation [18–20]. Such approaches would subject financial burden on the government and also create unwanted dependency on the external aid among the communities. Relying solely on food supplementation from external assistance without proper education is insufficient to address nutritional issues among the vulnerable population. A more effective intervention strategy should incorporate positive behavioural changes that can support the targeted community in actively seeking practical solutions in their local setting.

In light of this, the introduction of more inventive approaches is deemed necessary to enhance the impact of the intervention. Since the daily diet of young children is mainly controlled by their mothers [21], maternal feeding practices play a vital role in the solution of the undernutrition issue. Hence, innovative interventions such as the positive deviance (PD) approach that encourage positive and sustainable change in maternal behaviours when feeding their children can highly effective in reducing undernutrition among children under-five.

The PD is described as any uncommon outlying yet successful norms, behaviours, or practices that may be beneficial and able to successfully solve the community problem [22]. The approach focuses on recognising the PD that causes a person to outperform others in the same community [23]. Such practice is eventually shared within the community to deal with the community’s problem [24]. This approach also emphasizes on the application of culturally-acceptable solutions in the local setting that can potentially promote positive behavioural changes among mothers. Eventually, it is hoped that they will take the necessary initiatives to achieve healthy weight gain in their children.

To date, there is a lack of local studies on interventions that encourage positive and sustainable behavioural changes that can improve child nutritional status among primary caregivers of young children. Elsewhere, a few intervention studies are using the PD approach to improve the nutritional status of undernourished children in countries such as Ecuador, Ethiopia, and Burundi [25–29]. Nonetheless, mixed findings have been shown in other countries. A few studies showed that the approach was successful in improving nutritional status with a reduction in undernutrition prevalence, significant weight gain, and increased nutrient intake of children while some studies showed no effect [24, 30, 31]. Apart from the positive outcomes in terms of growth and nutrient intake, the effectiveness of a PD programme in improving other aspects such as food security status and nutritional knowledge of mothers remains unknown.

Therefore, this study aims to describe the study protocol of a nutrition programme on urban poor children using the PD approach. Firstly, the PD behaviours of the urban poor households will be identified and subsequently these findings will be applied to develop a locally and culturally-acceptable solution to tackle the child undernutrition issue among the urban poor. These solutions include a combination of nutrition education with peer-led cooking session and promotion of positive behavioural changes among caregivers of young children. The objectives of this study are to explore maternal feeding practices and foods that are fed to children of both positive deviant (PD) family (poor family with well-nourished children) and non-positive deviant (NPD) family (poor family with undernourished children) as well as to evaluate the effectiveness of the programme in reducing undernutrition among urban poor children aged 3 to 5 years old.

## Materials and methods

The underlying protocol follows SPIRIT Statement (S1 Checklist) and CONSORT Checklist (S2 Checklist).

### Study design

This is a mixed-method study that involves both qualitative and quantitative methods. In phase one of the study, a qualitative research method will be used while in phase two, a two-armed cluster RCT will be conducted. The primary outcome of the RCT study will be weight-for-age z-score (WAZ), while the secondary outcomes will include height-for-age z-score (HAZ), weight-for-height z-score (WHZ), nutrient intake, diet quality, food security status and nutritional knowledge of mothers. The study population includes 3 to 5 years old children with their mothers who live together in the low-cost PPR flats in Kuala Lumpur. Children aged 3 to 5 years old are recruited because when compared to younger children (<2 years old), older children are at higher risk of undernutrition [32].

#### Phase 1 (qualitative study)

In the first phase of the study, the identification and classification of families into PD family and NPD family based on the children’s anthropometric measurements will be first carried out. Following that, a qualitative study in the form of focus group discussion (FGD) with semi-structured interviews will be conducted. The inclusion criteria for the participants in Phase I, i.e. the mothers are: Malaysians, above 18 years old with children aged 3 to 5 years old, living in low-cost PPR flats and with a monthly household income of <RM3000. The monthly household income is set at RM3000 because this is the requirement to apply for residing in a public low-cost PPR flat [33]. For a household with more than one child aged 3 to 5 years old, the oldest child will be recruited as the index child. This is because older children have more autonomy in their daily diet, causing them to have higher chances of undernutrition compared to younger children [21]. Mothers whose children are taken care of by other adults will be excluded. Mothers whose children are diagnosed with chronic diseases, under treatment for communicable disease, learning disabilities will also be excluded as these children may have different eating habits and nutritional status, thus possibly contributing to a biased outcome. Apart from that, mothers with overweight or obese children (WHZ > +2SD) will also be excluded. Lastly, mother-child dyads who are involved in any other intervention or clinical research, mothers with mental disabilities, and mothers with low literacy level to fully understand the content of the questionnaires will also be excluded as they might not be capable to follow and involve completely in the present study.

A list of flats under the People’s Housing Project in Kuala Lumpur will be acquired from the Kuala Lumpur City Hall. By using the purposive sampling method, two flats with the top two highest number of housing units will be chosen. Then, the researcher will contact the chairperson of the community representative committee to obtain a list of households with children aged 3 to 5 years old. Household visits will be made to the selected flats. Purposive sampling will be used to select the subjects with well-nourished or undernourished child. Two focus groups will be recruited from the PD family [family with well-nourished under-five child who has normal growth indicators (-2SD≤Z≤ +2 SD for WAZ, HAZ and WHZ)] while another two from the NPD family [family with undernourished child who is either underweight, stunting or wasting (WAZ/HAZ/WHZ<-2SD)], with each group consists of 6 participants. Thus, the estimated number of participants will be 24 or the participants will be interviewed until a saturation point is reached. Data saturation is considered reached when no additional insights can be identified from the participants and data start to be repetitive.

Before the interview, the participants will be approached to obtain their informed consent and permission for audio-recording. A short briefing will be given to the participants. After that, semi-structured face-to-face interviews in the form of FGDs will be held in the community hall in the flat. The researcher serves as a moderator to regulate the whole process of the FGDs. Every informant in the group will be given a chance to answer each question. Each session of FGD will take approximately one hour. An interview protocol, including a script and a list of questions pertaining to maternal feeding practices, will be prepared in advance. The examples of questions are “*What food did you usually feed your child in the morning*, *noon and evening*?*”* and “*What do you do when your child does not want to eat*?”.

After collecting the qualitative data, thematic analysis [34, 35] with NVivo 12 (Melbourne, Australia), which is the computer-assisted qualitative data analysis software (CAQDAS), will be used to analyse the data. Peer debriefing and data source triangulation will be performed. To perform data triangulation, multiple data sources will be collected from two different groups, namely the PD family and NPD family, to obtain information on feeding practices and food being fed to their children.

#### Phase 2 (quantitative study)

A two-armed, cluster randomised controlled trial will be carried out to determine the effectiveness of a nutrition programme. The selection criteria for respondents in this phase will be almost similar to those in Phase 1, except for one criterion related to children. In Phase 2, only undernourished children (either underweight, stunting, or wasting) aged 3 to 5 years old will be recruited to evaluate the effectiveness of the intervention programme in this particular group. The WHO Child Growth Standards for children under 5 will be used to classify children into underweight (WAZ< -2SD), stunting (HAZ< -2SD) and wasting (WHZ< -2SD) [36]. The exclusion criteria in Phase 2 are similar to the exclusion criteria in Phase 1.

### Sample size estimation

Sample size will be calculated using the formula for a RCT comparing two groups of equal size [37, 38], two-tailed test with 80% power and 5% significance level. As there was minimal information on the nutritional status of urban poor children and the effectiveness of nutrition programme in the setting of Malaysia, this study used the information obtained from a previous study that was not done in Malaysia but was using PD approach and it is relevant to the context of the present study [25]. By referring to the mean and SD of the WAZ of children aged 36 to 48 months in the intervention (using the PD approach) and comparison groups from a previous study [25], the minimum sample size required will be 28 in each group. It is estimated that there are around 10 households with undernourished children under five in a cluster of PPR flat. Thus, the cluster size per arm, m, is assumed to be 10 and the intra-cluster correlation coefficient (ICC) is assumed to be 0.03 [39]. Hence, with a calculated design effect of 1.27 and a non-response rate of 20%, the sample size is increased to 41 in each group.

### Sampling

In Phase 2, the sampling will be based on the similar list of flats and households used in Phase 1, i.e. the two PPR flats with the highest and second-highest number of housing units. A total of fourteen flats, seven each for intervention and comparison groups, will then be chosen using a simple random sampling. Cluster randomisation will be performed to reduce the chance of contamination. Each flat is a cluster unit. Since the area of PPR flats in Kuala Lumpur is classified into four zones, residents from two zones will be randomly selected as intervention groups while another two zones as comparison groups. Then, by using the list of households with children aged 3 to 5 years old from the seven targeted flats in the respective zones, household visits will be carried out again to conduct anthropometric measurements on the children to obtain a list of households with undernourished children. After that, the mother-child dyads from the list who meet the inclusion and exclusion criteria will be number coded by the researcher and randomly selected to participate in the present study. The randomization process will be conducted using a computer-generated software (www.randomizer.org) by an independent third party. Participants (n = 82) will be randomized using permuted block randomization into two groups: intervention or comparison group in a 1:1 ratio.

### Development of materials for intervention

All materials will be developed by referring to the published PD guidelines or education modules obtained from previous PD studies. The materials will also refer to official nutrition-related guidelines such as the Malaysian Dietary Guidelines (MDG) for Children and Adolescents [40] and recipes or menus from the Nutrition Society of Malaysia. The findings related to the PD behaviours and foods from the PD family obtained from FGDs in Phase 1 will also be incorporated into the materials. The tools will be designed in a user-friendly form such as leaflets, infographics, and posters so that the messages can be easily delivered to and understood by the target population, i.e. the mothers.

### Implementation of intervention

For the intervention group, a nutrition programme consisting of an education session with peer-led cooking session and rehabilitation session will be provided. The programme will be known as the Nutrition Education and Rehabilitation Session (NERS). It is planned as a 3-month intervention with a total of 12 sessions, each session lasting for two hours for one day in a week, making up a total of four days in one month. The two-hour session consists of half-an-hour of education lesson followed by one and a half-hour of peer-led cooking session. The rehabilitation session will constitute the remaining days before the next education session. The overall education session will be conducted by the researcher. However, the cooking session will be led by volunteers who are recruited from the PD families in the initial stages of the study. Peer-led cooking session will encourage the involvement and active interaction from the volunteers to assist participating mothers in the food preparation processes for child feeding. The participating mothers will need to bring along their undernourished children during this session and feed their children with the prepared meals when they finish cooking. During the process, volunteered mothers from PD family are encouraged to share their knowledges of preparing food and child feeding to the participating mothers to enhance the PD effect. Five to six mothers will be assigned in a group to prepare meals for five to six children. Each group will be assigned with one volunteer to help them throughout the process. Fresh ingredients will be provided. The meal in each cooking session will be prepared according to the pre-planned NERS menu and will be fed as a snack or additional meal to children. This programme will also include a growth monitoring session whereby the participating mothers weigh their children during each session of NERS before the start of the education session. A weighing scale will be provided by the researcher. Mothers will be trained to weigh their children so that they can monitor their children’s nutritional status.

### Comparison group

Mothers from the comparison groups will receive all the education materials and menus used in the programme. In order to encourage continued participation and to prevent dropouts from the comparison group during the 3-month programme, incentives will be provided for children who complete the anthropometric measurements and mothers who return the completed questionnaire during each follow-up.

### Training of volunteers

Volunteers (mothers of well-nourished children) will be recruited from PD families that are identified in the initial phase. Fifteen or more volunteers will be recruited. They will play a main role in conducting the peer-led cooking session and actively supporting the participating mothers of the intervention group from the NPD family to acquire a new skill. A few discussions will be conducted with the volunteers to equip them with the necessary knowledge and skills so that can lead the cooking demonstration and help the participating mothers.

### Measurements

All the measurements in the study will be taken at the baseline, immediate post-intervention, and 3-month post-intervention for both intervention and comparison groups. A Malay language self-administered questionnaire on sociodemographic characteristics, nutritional knowledge, and food security status will be answered by mothers. The sociodemographic background of the mother-child dyads will be self-reported by the mothers, including information on both the child and the mother.

#### Primary outcome

Anthropometric measurements of the children will be performed by the researcher. The height of the children will be measured to the nearest 0.1 cm by using a SECA Body Meter 213 (SECA, Germany). Weight will be measured to the nearest 0.1 kg by using a TANITA Digital Weight Scale HD662 (TANITA Corporation, Japan). The collected anthropometric data will be entered and analysed by using the WHO Anthro Survey Analyser [41]. Z-score for weight-for-age (WAZ) will be calculated and underweight (WAZ<-2 SD) children will be identified according to the WHO Child Growth Standards for children under the age of 5 [36].

#### Secondary outcomes

In addition, z-scores for height-for-age (HAZ) and weight-for-height (WHZ) will be calculated. Stunted (HAZ<-2 SD) and wasted (WHZ<-2 SD) children will also be identified based on the WHO Child Growth Standards [36]. Mothers will be interviewed by the researcher on the dietary intake of their children based on the 24-hour dietary recall for three days, including two weekdays and another day on the weekend. For intervention group, the data collection of dietary intake at immediate post-intervention will take place one day after the last session of the program.

Dietary intake will be entered into the Nutritionist Pro^™^ Diet Analysis Software (Axxya, USA) based on the Nutrient Composition of Malaysian Foods database. The adequacy of macronutrient and micronutrient will be determined by referring to the Acceptable Macronutrient Distribution Range (AMDR) of macronutrients and compared with the Recommended Nutrient Intakes (RNI) for Malaysians [42]. Diet quality will also be evaluated using the Healthy Eating Index for Malaysians (HEI) [43]. The possible composite HEI score ranges from 0 (low diet quality) to 100 (good diet quality), in which less than 51% indicates poor diet, 51 to 80% indicates diet requiring improvement, and more than 80% indicates good diet quality [43]. In addition, for the intake of food promoted during the program, mothers will be asked regarding the inclusion of the food in any meals, the frequency and quantity of the foods eaten by their child in the past two weeks.

Nutritional knowledge level of mothers will be assessed by using a questionnaire adapted from a previous study among Malaysian adults [44]. This questionnaire consists of 20 questions on nutritional knowledge related to calorie, carbohydrate, food pyramid, fat, protein, and others. The total scoring will be the total number of correct answers. Based on the percentage of the score, those less than 50% will be classified as unsatisfactory, between 51% to 74.9% as moderate, and more than 75% as satisfactory [44].

**Fig 1:**
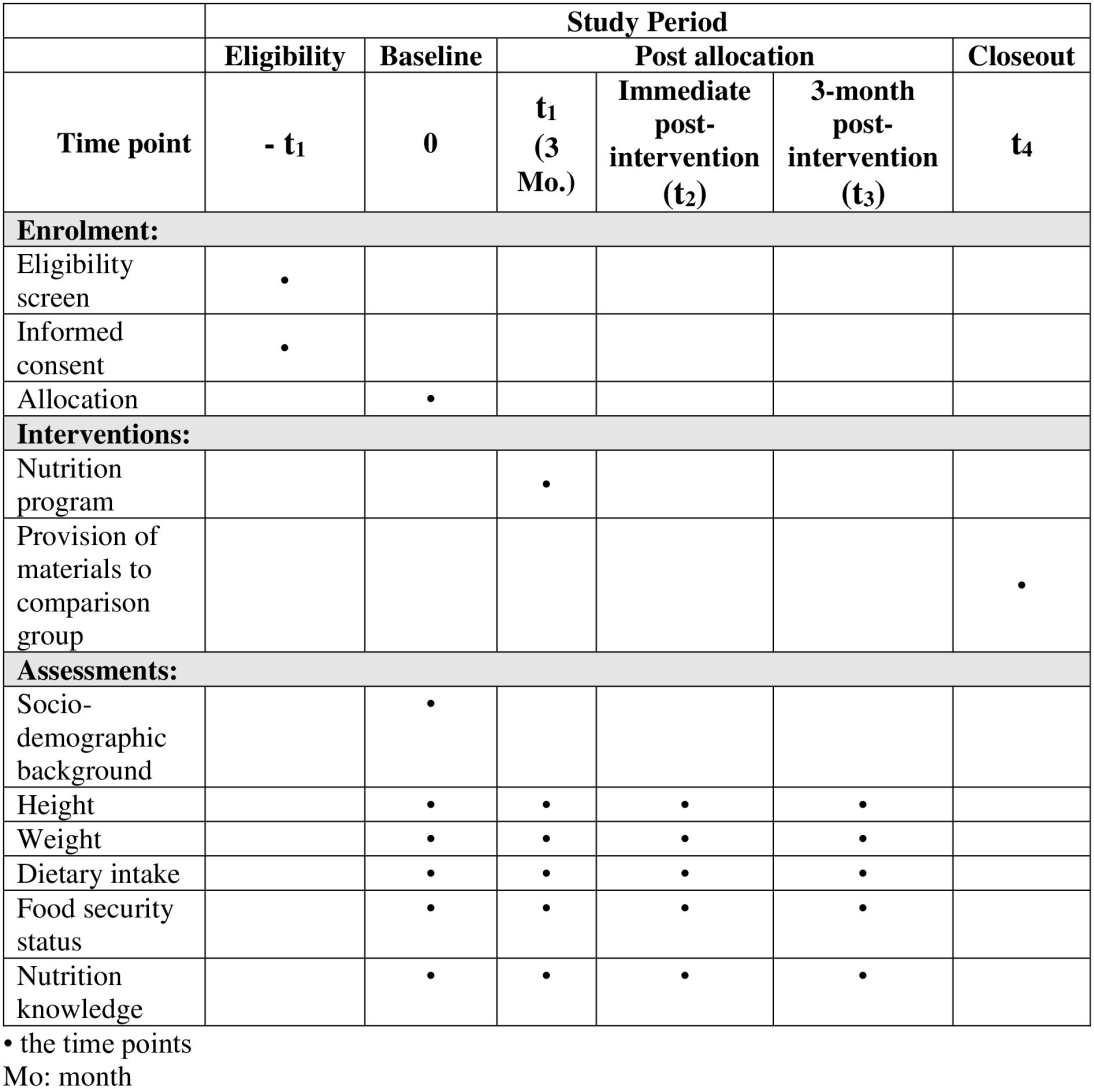
The study timeline. • the time points; Mo: month.

Food security status will be determined with the USDA six-item short form of the Household Food Security Survey Module [45]. It is a self-reported tool that contains six questions related to household food security to be answered by mothers. The scoring will be calculated by summing up the total number of affirmative responses. Respondents with less than two affirmative responses will be classified in the “food secure” group while those with two or more affirmative responses will be categorised as “food insecure”. The questionnaire has been previously used in Malaysia [46]. The overview of the study timeline is shown in Fig 1.

**Fig 2:**
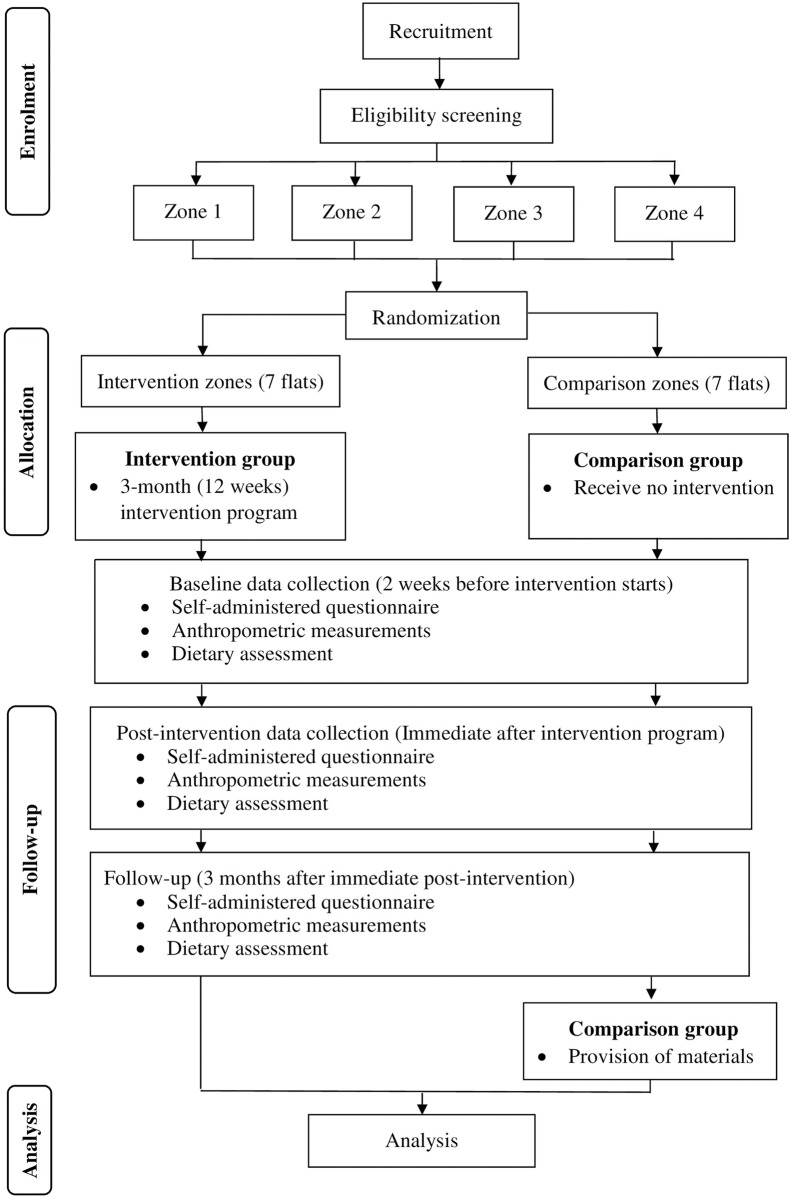
CONSORT diagram of the study flow.

Fig 2 shows the CONSORT flow diagram of the present study protocol.

### Ethics approval and consent to participate

Ethical approval was obtained from the Ethics Committee for Research Involving Human Subject (JKEUPM) of Universiti Putra Malaysia (Reference no: JKEUPM-2020-349) on 20 December 2020. Permission to conduct the study in the flats was obtained from Kuala Lumpur City Hall and management office of the selected PPR flats. The written informed consent will be obtained from the participants. The study was registered at ClincalTrials.gov (Identifier: NCT04688515) on 29 December 2020.

The purposes of the study will be explained by the researcher to the participants as well as the procedures to be performed, their discomforts and risks, and the guarantees of confidentiality. The participants will voluntarily agree to participate in this study and will be able to withdraw consent at any time, before or during it, without any penalty. There is minimal risk in this study. The only concern is the food safety issue during the cooking demonstration session in the intervention group. However, by taking precautions such as providing fresh ingredients, washing hands and wearing necessary protective coverings, the possible risk can be reduced. Moreover, mothers will also be reminded to notify the researcher if their children experience discomfort or show symptoms such as vomit and diarrhoea after consuming the meals provided in the program. The child will be taken to the nearby clinic or hospital as soon as possible for further treatment.

### Data monitoring

Investigators from the Department of Nutrition will be involved in the design and the implementation of the interventions. The principal investigator will be responsible for supervising the administrative management of the study and the execution of the trial, record keeping and data management. This study will evaluate the nutrition programme using positive deviance approach that is highly unlikely to be related to serious adverse events. Thus, no data monitoring committee or auditing will be needed. However, the principal investigator will take care of monitoring and will be responsible for the progress of the study.

### Potential amendments

The recruitment of participants is scheduled to be carried out in 2022. Phase 1 of study (FGD) is expected to be carried out from February to June 2022 and Phase 2 (RCT) is expected to start in September 2022. To date, the recruitment of participants for Phase 1 has begun and is on-going. However, this might be temporally suspended as a response to controlling the COVID-19 pandemic in the country and that would affect the project timeline. In case a need for modification should arise, it will be registered and reported in this journal.

### Statistical analysis

All data collected will be entered and analysed by using IBM SPSS Statistics 26 (SPSS Inc., Chicago, IL, USA). The normality distribution of data will be tested by evaluating the values for skewness and kurtosis between -2 and +2 [47]. The differences in anthropometric measurements (WAZ, HAZ, WHZ), dietary practices (energy, macronutrient and micronutrient intakes, diet quality and consumption of the promoted foods) of children, food security status, and nutritional knowledge of mothers between intervention and comparison groups at baseline (before intervention), immediate post-intervention, and 3-month post-intervention will be tested using the independent-samples t-test and repeated measure ANOVA. Furthermore, Generalised Linear Models (GLM) will be performed to evaluate the effectiveness of the programme. All p-values are two-tailed and the level of significance will be set at p<0.05 and post-hoc adjustment will be performed for multiple comparisons. All data will be treated following an intention-to-treat approach and multiple imputation will be used for treating missing data whenever possible.

## Discussion

This study aims to evaluate the effectiveness of a nutrition programme that is going to be developed by using the PD approach to reduce undernutrition among urban poor children. This approach aims to tap into locally available and affordable resources to provide culturally acceptable and sustainable solutions for resource-challenged communities, i.e. the urban poor households to overcome undernutrition issues. We hope to recognise some practices, particularly in terms of feeding practices and foods being fed to children that are unique, applicable, and suitable for the urban poor population. From the perspective of the urban poor population, these practices that originate from their neighbourhood are locally available, accessible, affordable, and culturally adaptable. Compared to other approaches that follow or adopt common good practices from other countries, such locally-originated practices identified by the PD approach would be more relevant and convincing to be applied by the urban poor community in Malaysia.

The PD findings identified in the first phase of the study will also be incorporated as part of the educational materials in developing the intervention programme in the second phase. By doing so, it will facilitate the process of passing the knowledge to mothers as those knowledges shared during the programme originate from peers who live in the same limited-resource living environment as them. This may further enhance the confidence of mothers of undernourished children that they are also capable of improving the nutritional status of their children. They will believe that local solutions exist and are available and accessible to them. Subsequently, this will empower them to change their behaviours and practise the necessary skills to improve the nutritional status of their children.

In contrast to previous NERS designated in guidelines, the education session in this study will be conducted by the researcher instead of volunteers from the community. The modules of education session in this study are modified according to government guidelines so that the nutritional knowledge provided in the process is suitable for the target group. Nevertheless, the concept of a peer-led cooking session is retained. The cooking session will be led by volunteers from the PD family. As peers with well-nourished children living in the same environment as the participating mothers, the knowledge and experiences shared by them with the participating mothers can enhance the PD effect and further empower positive behavioural change in the mothers of undernourished children [48].

Furthermore, the PD programme in this study will also include two specific sessions for rehabilitation and growth monitoring purposes. The purpose of the rehabilitation session is to initiate rehabilitation in undernourished children and allow the mothers to practise their learned skills at home comfortably in a supportive environment [48]. For the growth monitoring session, the mothers, rather than a researcher, will be the person who weighs the children. This will be an important initiative for mothers to monitor children’s nutritional status. When they observe that their children are gaining weight and becoming healthy under their care, it will boost their confidence to feed their children appropriately.

Compared to this study, previous intervention studies that used the PD approach focused predominantly on improving the growth outcomes and nutrient intake of children. Maternal perspectives were rarely studied. As the primary caretakers, mothers are the most likely persons to influence and control the daily nutrient intake of young children. Thus, this study emphasises the role of mothers in improving the nutritional status of urban poor children and maternal factors such as their food security status and nutritional knowledge will be studied. In addition, mixed findings are obtained from previous PD studies. The present study, therefore, aims to provide further evidence on the effectiveness of using the PD approach in helping undernourished children to gain weight healthily through a positive behavioural change in their mothers. This is in line with the global initiatives in decreasing child undernutrition. For example, the second goal of Sustainable Development Goals (SDGs) is to eradicate all forms of malnutrition by 2030 by ending hunger as well as achieving food security and improved nutrition [49]. Furthermore, Malaysia also aspires to achieve the six global nutrition targets by 2025 with the ultimate target of a 40% decrease in the number of children under-five who are stunted as well as a reduced level of childhood wasting to less than 5% [50].

It is worth noting that the design of this study does not fully allow us to conclude that the effectiveness of the programme is entirely due to the impact of the food provided as part of the intervention and the use of peer educators. To date, no intervention studies using the PD approach have been conducted in Malaysia. In light of the government initiatives to tackle undernutrition issues in under-five children, the findings of this study will provide vital insight as to the innovative approaches that are necessary for decreasing the high prevalence of child undernutrition in the country. In summary, this study intends to apply the PD approach as an innovative method to discover locally available and culturally adapted solutions, i.e. the outperformed feeding practices and PD foods, so that they can be used to initiate positive behavioural change among mothers of undernourished children and to empower them to promote healthy weight gain in children.

## Supporting information

S1 Checklist Checklist of recommended items in a clinical trial protocol. (DOC)

S2 Checklist Checklist of information to include in a randomised trial. (DOC)

S1 File Study protocol approved by JKEUPM. (PDF)
