# Supplementary material for: A nutrition programme using positive deviance approach to reduce undernutrition among urban poor children under-five in Malaysia: A cluster randomised controlled trial protocol
Source: PLoS One. 2022 Oct 13;17(10):e0275357. doi: 10.1371/journal.pone.0275357 (PMC9562161; doi:10.1371/journal.pone.0275357)
Supplement: S1 File — (PDF) [file pone.0275357.s003.pdf]

RESEARCH PROPOSAL

EFFECTIVENESS OF A NUTRITION PROGRAM USING POSITIVE DEVIANCE APPROACH TO REDUCE UNDERNUTRITION AMONG URBAN POOR CHILDREN UNDER FIVE IN KUALA LUMPUR, MALAYSIA

Principle Investigator : **Assoc. Prof. Dr. Gan Wan Ying**

Co- Investigators : **Assoc. Prof. Dr. Norhasmah Sulaiman**
Assoc. Prof. Dr. Chin Yit Siew
Chek Lok Poh

Document submitted for ethical approval from JKEUPM 2020

CHAPTER 1
INTRODUCTION

1.1 Background

Globally, childhood malnutrition remains a public health concern. Malnutrition can be diverse from undernutrition to overnutrition (Development Initiatives, 2018). The indicators used to determine childhood malnutrition include weight-for-age, height-for-age and weight-for-height based on the WHO Growth Standard (WHO, 2006). A young child, primarily refers to those under the age of 5, is suffering from undernutrition when the child is lacking of adequate nutrition that necessary for proper growth and health due to direct or indirect causes such as not having enough food (UNICEF, 2019). Undernourished children who fail to grow in height and weight as other normal children can be underweight, stunting or wasting. Children are underweight when they are too thin for age, stunted when too short for age and wasted when too thin for height (UNICEF, 2019).

In 2019, it was estimated that 6.9% or 47 million children under 5 were wasted globally, in which approximately 14.3 million were severely wasted, whereas, stunting is threatening the lives of an estimated 21.3% or 144 million children under 5 globally (UNICEF, WHO & The World Bank, 2020a). Besides, underweight is affecting 13.5% of the children in the world in 2017 (UNICEF, WHO & The World Bank, 2020b). In Malaysia, according to the National Health and Morbidity Survey (NHMS) 2019, the national prevalence of underweight, stunting and wasting among children under 5 was 14.1%, 21.8% and 9.4%, respectively (IPH, 2020). These findings indicate that undernutrition remains alarmingly high both globally and nationally.

In line with that, a set of six global nutrition targets by 2025 had been endorsed which include an achievement of 40% reduction in the number of children under 5 who are stunted and a reduction or maintenance of childhood wasting to be less than 5% (WHO, 2014). Furthermore, another global target namely the Sustainable Development Goals (SDGs) also stated in its second goal to end all forms of malnutrition by 2030 by ending hunger, achieving food security and improved nutrition (United Nations, 2015). However, the prevalence of undernutrition in Malaysia is declining too slowly to meet global targets on time (UNICEF, WHO & The World Bank, 2019).

The endeavour in unravelling child undernutrition at global and country level is undoubtable. Yet, a recent global report revealed that the processes in addressing child undernutrition issues are less equity-sensitive (Development Initiatives, 2020). The report asserted the existence of inequalities in the field of undernutrition in term of sex, age, wealth status, education level and location. For example, a larger disparity of stunting prevalence can be seen between low-income (43.6%) and high-income household (18.6%) (Development Initiatives, 2020). These facts indicate that inequalities are truly exist and there are many groups of people have been marginalized from the progress of addressing undernutrition issues, especially those urban poor population.

There is growing evidence showing that there is still an extensive proportion of the population struggling to catch up with the rapid development of country such as the low-income group (Shahar et al., 2019), indicating the emergence of ‘new poor’ or more known as urban poverty (Khoo et al., 2018). Urban poverty, which is generally referred to a situation when the urban household has low income or income falls below the poverty line even though there are family members who are still working or being paid within the household, probably due to the increasing living cost in urban areas (Khoo et al., 2018).

In the few decades, the total population growth in Malaysia had increased for almost three-fold from 13.7 million in 1980 to 32.68 million in 2019 (Department of Statistic Malaysia, 2020) and Malaysia is urbanized rapidly (United Nations, 2019). The population who lived in

urban areas had doubled within 30 years, increasing from 34.2% in 1980 to 71% in 2010 (Department of Statistics Malaysia, 2010). Within Malaysia, Kuala Lumpur and Putrajaya have been reported to be fully urbanized (100%) (Department of Statistics Malaysia, 2010). Besides that, the low-income group is referring to the B40 income group in the country, who represent the bottom 40% of household income distribution of Malaysian population with income less than RM4850 (Department of Statistics Malaysia, 2020). In order to address the issues of squatters and residence requirements of this low-income group in urbanized area such as Kuala Lumpur, low-cost flats are built and being sold or rent with affordable price by government to the B40 income group through the implementation of People's Housing Project (*Program Perumahan Rakyat*, PPR) (Ministry of Housing and Local Government, 2018).

Urban poverty is not merely a concept that defines through income level but indeed it always in line with the deprivation of basic privileges such as lack of government policy, inadequate law protection, inability to reach out to financial services, poor housing condition and high living costs (Tacoli et al., 2015), which might greatly affect nutritional status of an individual. A UNICEF study on urban child poverty and deprivation Kuala Lumpur showed that urban poor population living in low-cost flats were indeed living in a harsh environment without safe and sound living environment, and facing food insecurity problem (UNICEF, 2018). Those children who are residing in low-cost flats suffered the most as they do not have the capabilities to change their current living situation and they become more susceptible to undernutrition, proven by the findings of high prevalence of underweight (15%), stunting (22%) and wasting (20%) (UNICEF, 2018). Hence, urban poor children who are living and growing up in such an underprivileged environment have much needed attention from stakeholders so that appropriate action can be taken.

There are various programs such as Nutrition Activities at Childcare Centres (TASKA), Rehabilitation Program for Malnourished Children (PPKZM) and Community Feeding Program have been conducted throughout Malaysia with the intention to decrease the phenomena of childhood undernutrition among poor families but limited success has been seen (Wan Manan et al., 2019) and the prevalence of undernutrition remains high in Malaysia (IPH, 2020). Therefore, future nutrition programs may need an immediate change by employing more intensified actions and effective strategies such as focusing more on increasing nutritional knowledge and empowering the adoption of positive health and eating behaviours.

Since young children are generally relying on their mothers to obtain daily diet (UNICEF, 2019), maternal feeding practices seem to play a vital role in implementation of interventions for undernourished children. Hence, alternative interventions such as encouraging positive and sustainable change in maternal behaviour when feeding their children might be efficient in reducing undernutrition in children under 5. Positive deviance (PD) approach that focuses on recognising any uncommon outliers yet successful either in behaviour, attitude or condition that cause a person to be outperformed from others in a community (Herington & Fliert, 2017) and spreading those inherent wisdom in dealing with community's problem within a community (Albanna & Heeks, 2019) might be a suitable approach to reduce undernutrition.

Positive deviance (PD) is defined as any unusual norm, behaviours or practices that is beneficiary and capable of successfully unravelling the community's problems (The CORE group, 2002). Hence, in the same situation of socio-economic deprivation as peers, individuals, who succeed in finding a way to conquer challenges and deliberately practice those uncommon but beneficiary actions or behaviours, have been identified as positive deviants (Schooley & Morales, 2007). Positive deviance in the context of child nutrition is a community-based participatory nutrition education approach that is explicitly defined as adaptive child care practices, positive hygiene practices, and feeding practices which allow children to grow

normally in a harsh environment with limited resources (Zeitlin et al., 1990). It is also an approach rooted in the concept that the problem occurred in a community can be resolved by using solutions that already existed within a community (Sternin et al., 1998).

The PD approach in nutrition program might function as a tool to discover positive deviant practices in positive deviant mothers of well-nourished children and spread this wisdom to mothers of undernourished children who live in the same resource-challenged environment. Those mothers are empowered to initiate positive behaviour change in feeding their children as a start-off to promote healthy weight gain in their children (Herington & Fliert, 2017). Hence, a nutrition program developed by using PD approach might convince mothers that solution to promote weight gain in children is affordable, existed and available within their own community. By participating in such program, they may learn from and empower by peers who outperformed in the same resource-challenged setting during the process of adopting new and good behaviours, in which could further enhance their confidence in feeding their children and achieving healthy weight gain in their children.

1.2 Problem Statement

Urbanisation in Malaysia has caused hasty increase in urban population, which contributes to numerous issues such as escalating economic and social costs as well as the shortage of housing that diminished their living standard (Chamhuri et al., 2016). Urban poor population are surviving and living in a harsh environment with limited resources. They often suffer from food shortage and have diminished ability to reach nutritious diet which causes them to be undernourished (Tacoli et al., 2015). However, the urban poor population is somehow being ignored and less emphasised in the effort to end poverty and malnutrition internationally and nationally due to the norm and expectation that people who capable to resident in urban area are richer and having better living quality. Despite of adults in urban poor families are bearing the consequences of urbanisation and poverty, those children who grow up in such families are more vulnerable than their parents with higher risk of undernutrition.

Under-five children who are undernourished are vulnerable and suffered more than expected. They might experience growth and developmental delay, particularly on brain and cognitive development which subsequently causes them to suffer from severe irreversible physical and cognitive damage such as learning difficulties, future adulthood disability, recurrent infections and multiple diseases due to diminished immunological capacity to defend against diseases and others (De & Chattopadhyay, 2019). According to the Global Nutrition Report, undernutrition is responsible for about 45% of deaths among children under 5 in both low and middle-income countries (Development Initiatives, 2018). Hence, recognising the unfavourable impacts of malnutrition on young children, effective intervention shall begin in early childhood to decrease high prevalence of child undernutrition and also to minimize the detrimental consequences in adulthood.

In the effort to reduce child undernutrition, children aged 3 to 5 years old are less focused compared to children aged below 3 years old (Ayana et al., 2015; Irarrázaval et al., 2018; Menon et al., 2015; Nowicka et al., 2014; Pravana et al., 2017; Tiwari et al., 2014). This might be due to children below 3 are mainly depending on mothers for daily diet which highly associated with maternal feeding practices such as exclusive breastfeeding, weaning and complementary feeding that need extensive nutrition knowledge (UNICEF, 2019). In contrast, children above 3 might have more autonomy in their daily diet but their food environment are still subjected to adult's influence, especially mothers who feed them daily (UNICEF, 2019). This asserted the importance of the role of mother to shape child eating behaviours when

feeding and providing foods to their children. Hence, more studies targeting this age group are required.

In the past ten years, extensive intervention studies have been conducted throughout Malaysia focusing on child nutrition issues but there are lack of intervention studies focusing on reducing undernutrition among children under 5. Local intervention studies are mostly emphasized on tackling childhood obesity among school children (Koo et al., 2018; Yusop et al., 2018; Norliza et al., 2018; Wafa et al., 2011). For those local studies that targeted young children with nutrition issues, many of these studies are focused on other population such as children living in rural or remote places and indigenous population (Phua, 2015; Siti Fatihah et al., 2019; Wong et al., 2015, 2018), but the health status of urban poor children have been ignored. Therefore, studies on this vulnerable group of children are urgently needed.

In relevant to that, Malaysia has put great commitment in reducing child undernutrition by introducing nutrition-related policies and programmes throughout the country. For example, the National Plan of Action for Nutrition of Malaysia III 2016-2025 (NPANM III) has illustrated few programs that focus on reducing undernutrition for all age groups by referring to both global policies such as Global Nutrition Targets 2025 and SDGs 2030 and local policies or action plans such as the National Nutrition Policy of Malaysia 2005 and Ministry of Health Malaysia Strategic Plan 2016-2020 (NCCFN, 2016). The nutrition programmes specific for children age 6 years old and below include Nutrition Surveillance, Rehabilitation Programme for Undernourished Children (PPKZM), Community Feeding Programme, and Nutrition Activities at Childcare Centres (TASKA) (NCCFN, 2016).

The Rehabilitation Program for Undernourished Children (PPKZM) is a program that was launched in 1989 and is still undergoing throughout Malaysia (NCCFN, 2016). This program targets undernourished children age 6 months to 6 years who are from poor family with the purpose of improving their nutritional status by supplying food baskets containing basic foods such as rice, rice noodles, flour and eggs for at least 6 months (Ministry of Health Malaysia, 2015). However, with the implementation of this program, the achievements of this program are doubtful due to challenges such as limited success rate of weight gain and ignorance of proper diet by tempting to use packages with multivitamins and special milk as quick measures to achieve weight gain (Wan Manan et al., 2019). In the light of this, introducing or incorporating a more inventive approach into these programs could be necessary in order to further catalyse the impact of intervention.

In fact, nutrition interventions that target undernourished children are commonly implemented in a more traditional way such as provision of supplemental foods (Ghodsi et al., 2018; Huybregts et al., 2012; Langendorf et al., 2014), growth monitoring (Bhardwaj et al., 2016; Gyampoh et al., 2014) and micronutrient supplementation (Eilander et al., 2010; Khan et al., 2011; Schoonees et al., 2019). In contrast, interventions that encourage the positive and sustainable change in behaviours among mothers, who generally is the primary caregiver of young children, to affect child nutritional status are less studied. This might indicate a need to introduce innovative approach in traditional nutrition program. One of such approach is PD approach that might be effective in promoting positive behavioural changes in mothers of undernourished children by empowering them to take initiatives to achieve healthy weight in children.

Interventions that encourage positive change in maternal behaviours to affect child nutritional status are less studied locally. There are a few intervention studies using PD approach to improve nutritional status of undernourished children in countries such as Ecuador, Ethiopia and Burundi (Calvince et al., 2015; Inamahoro et al., 2017; Kang et al., 2016; Nishat & Batool, 2011; Roche et al., 2017). Nonetheless, mixed findings have been shown, in which some were successful in improving nutritional status such as reduce in undernutrition

prevalence, significant weight gain and increase nutrient intake of children but some showed no effect (Albanna & Heeks, 2019; Bullen, 2011; Sosanya et al., 2018). Besides that, instead of growth outcomes and nutrient intake, the effectiveness of a PD program in improving other aspects such as food security status and nutrition knowledge of mothers are also less studied. Hence, more studies are needed to evaluate the effectiveness of a PD program in reducing child undernutrition.

Consequently, this study is designed to evaluate the effectiveness of a nutrition program using positive deviance approach in reducing undernutrition among urban poor children aged 3 to 5 years old in Kuala Lumpur by using mixed-method approach.

1.3 Research Questions

a) How and what do mothers from both positive deviant (PD) family and non-positive deviant (NPD) family feed their children?
b) What is the prevalence of undernutrition among urban poor children aged 3 to 5 years old in Kuala Lumpur?
c) Does the nutrition program using positive deviance approach is effective in improving anthropometric measurements (WAZ, HAZ, WHZ) and dietary practices (energy, macronutrient, micronutrient and diet quality) of children as well as food security status and nutrition knowledge of mothers between intervention group and comparison group at the baseline (before intervention), immediate post-intervention and 3-month post-intervention?

1.4 Significance of the Study

In Malaysia, the prevalence of undernutrition is still considered high and the progress in reducing child undernutrition is too slow to meet any under-five global targets. There is not much time left for Malaysia to catch up the progress and meet achievements on time. More intervention studies focusing on children undernutrition are needed. Hence, by using PD approach, this study hopes to empower positive behaviour change in mothers of undernourished children and encourage them to take initiatives to achieve and maintain healthy weight and dietary practices in their children.

The urban poor population are less emphasized and studied in previous studies. It is important to consider the health and nutritional status of this vulnerable group as they deserve to enjoy welfare and basic health services as others. Findings of this study could provide baseline data for future research focusing on urban poor children. Furthermore, PD approach used in this study seems to be applicable to urban poor population as this approach is an asset-based approach that utilizes local available and affordable resources in a resource-challenged community to resolve issue identified in the same community.

By using PD approach, it is possible to recognise outperformed practices which are unique in certain setting. In this study, the PD foods and feeding practices will be identified from urban poor population and are specific and applicable to this population. From the perspective of urban poor population, these foods and feeding practices found using PD approach are the local wisdoms that are locally available, accessible, affordable and culturally adaptable. Compared to other approaches, such as the use of common good practices or following good practices found in other countries, the outperformed practices to be identified using PD approach appear to be more relevant to the urban poor community in Malaysia.

When using the PD findings to develop education materials, the knowledge shared during sessions tend to be more convincing to the mothers of undernourished children. This is because those wisdoms share during program are originated from peers who live in the same limited-resource living environment as them. This may further enhance confidence of mothers

of undernourished children that they are capable to improve nutritional status of their children. They will believe that solutions are indeed locally existed, available and accessible which later empower them to practice skills acquired to improve nutritional status of their children.

Besides that, this study also aims to enhance nutritional knowledge of mothers. This study intends to provide sufficient nutritional information and knowledge to assist mothers in initiating and practicing healthy eating and feeding practices. It is hoped that those knowledge learned by mothers will be passed on to their children and that their children will be able to practice healthier lifestyle in future. This study also hopes to encourage mothers to involve actively in continuously monitoring nutritional status of their undernourished children by having periodically growth monitoring session as a way to further increase their confident in the progress of improving nutritional status of their children.

The present study also wishes to increase the alertness and awareness of stakeholders such as policy makers and nutritionists on the active role of mothers in feeding their children and the importance of positive behavioural change in improving nutritional status of children. Healthcare personnel such as nutritionists may also benefit from this study by understanding the role of a healthcare personnel in modulating and catalysing effective intervention to reduce undernutrition in children.

1.5 Research Objectives

1.5.1 General objective

To evaluate the effectiveness of a nutrition program using positive deviance approach in reducing undernutrition among urban poor children aged 3 to 5 years old in Kuala Lumpur.

1.5.2 Specific objectives

Phase 1

a) To identify positive deviant (PD) family and non-positive deviant (NPD) family.
b) To explore maternal feeding practices and food being fed to children from both positive deviant (PD) family and non-positive deviant (NPD) family.

Phase 2

c) To determine the sociodemographic characteristics (age, sex, ethnicity, parent's age, parent's working status, parent's occupation, parent's education level, parent's marital status, household size, monthly household income and financial assistance) of urban poor children.
d) To determine the prevalence of undernutrition (underweight, stunting, wasting) among urban poor children.
e) To develop and implement a nutrition program using positive deviance approach among urban poor children.
f) To compare the anthropometric measurements (WAZ, HAZ, WHZ) and dietary practices (energy, macronutrient and micronutrient intakes, and diet quality) of children as well as food security status and nutrition knowledge of mothers between intervention and comparison groups at baseline (before intervention), immediate post-intervention and 3-month post-intervention.

1.6 Research Hypothesis

There are significant differences in the anthropometric measurements (WAZ, HAZ, WHZ) and dietary practices (energy, macronutrient, micronutrient and diet quality) of children as well as food security status and nutrition knowledge of mothers between intervention group and comparison group at the baseline (before intervention), immediate post-intervention and 3-month post-intervention.

1.7 Conceptual framework

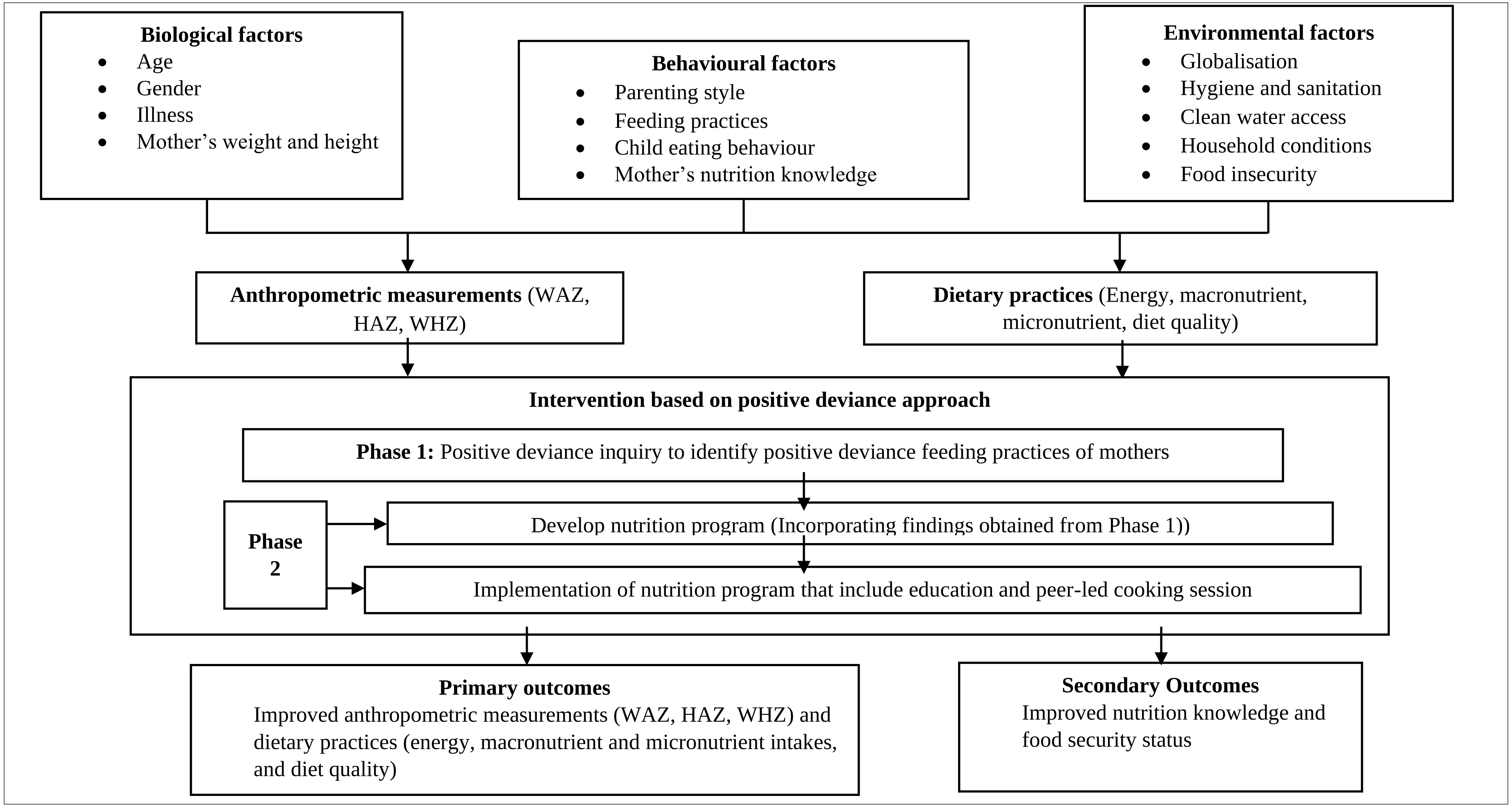

Figure 1.1: Conceptual framework of this study

CHAPTER 2
LITERATURE REVIEW

This chapter provide an insight into three main areas which include childhood undernutrition, urban poverty and positive deviance approach. In the first area, the definition, prevalence, factors and consequences of childhood undernutrition are discussed. In the section of urban poverty, the meaning of urbanisation, poverty and urban poverty with its related deprivations are reviewed. The last section provides deep understanding on the positive deviance approach and its effectiveness.

2.1 Undernutrition in Children

2.1.1 Definitions and prevalence

The absence of appropriate nutrition which caused by lack of food, inadequate nutritious food and diminished ability to utilise food is denoted as malnutrition (UNICEF, 2019). Malnutrition is a paradoxical term that describes either undernutrition or overnutrition, which can be ended up in multiple distinctive forms as divergent as underweight and obesity. Undernutrition, a form of malnutrition that characterized as a lack of adequate nutrition is not merely a devastating problem that only affects adults but also children. A child is undernourished when the child fails to grow in length and weight following an expected standard which can be manifested as underweight, stunting or wasting (UNICEF, 2019). To track physical growth of children, anthropometric measurements including weight and height are needed to be measured, which are important in identifying their nutritional status by classifying them according to WHO growth charts (WHO, 2006).

The indicators used in determining nutritional status of children from birth to 5 years include weight-for-age, height-for-age and weight-for-height in WHO growth standard (WHO, 2006). The classifications of nutritional status are according to the z-scores, whereby weight-for-age below -2SD is classified as underweight, height-for-age below -2SD is classified as stunting and weight-for-height below -2SD is classified as wasting (WHO, 2006). In other words, a child is underweight when he/she is too thin for age, stunting when he/she is too short for age and wasting when he/she is too thin for height (UNICEF et al., 2019).

The world is shouldered by the burden of malnutrition and the coexistence of multiple forms of malnutrition exist in many countries (Development Initiatives, 2020). Globally, almost 144 million children under age of 5 (21.3%) are stunted and 47 million children (6.9%) are wasted (UNICEF et al., 2020a). The global prevalence of stunting is decreasing throughout the years but it is declining too slowly, indicating that the progress to combat stunting is remaining slow (Development Initiatives, 2020). Data showed that the prevalence of stunting in 2000 was 32.5% and had decreased to 21.3% in 2019 (UNICEF et al., 2020a). However, for the prevalence of wasting, it showed an increasing trend throughout the years, growing from 4.9% in 2000 to 6.9% in 2019 (UNICEF et al., 2020a). For children who are underweight, the global prevalence was 13.5% in 2017 which had decreased from 15.4% in 2012 (UNICEF et al., 2020b). Although the prevalence of stunting and underweight around the world is showing declining trend, the prevalence of undernutrition is still alarmingly high which requires immediate actions.

Globally, the country that seemed to bear the most share of all forms of malnutrition is Asia, followed by Africa (UNICEF et al., 2019). More than half of children under 5 who were stunted (54%) or wasted (69%) lived in Asia while 40% of stunted children and 27% of wasted children in the world lived in Africa in 2019 (UNICEF et al., 2020a). In the region of South-eastern Asia, it was estimated that the prevalence of stunting in children was 24.7% (13.9 million) and about 4.7 million of children (8.2%) were estimated to be wasting (UNICEF et al.,

2019). The national prevalence of stunting among each country within South-eastern region is ranging from 4.4% to 50.9% in which Singapore documented the lowest prevalence (UNICEF et al., 2019). while Lao People's Democratic Republic documented the highest prevalence (UNICEF et al., 2019). For the national prevalence of wasting, which is ranging from 2.9% to 13.5%, Brunei Darussalam reported the lowest prevalence (UNICEF et al., 2019) while Indonesia reported the highest prevalence (UNICEF et al., 2019).

Similar to other South-eastern Asia countries, Malaysia is also bearing the burden of malnutrition with multiple forms. According to the National Health and Morbidity Survey (NHMS) 2019, the latest national prevalence of stunting in Malaysian children under 5 is as high as 21.8% (IPH, 2020). The prevalence has increased by 1.1% from 20.7% in 2016 (IPH, 2016). The reported latest national prevalence of stunting (21.8%) (IPH, 2020) is as high as the global prevalence (21.3%) (UNICEF et al., 2020a). For underweight and wasting, the prevalence was 13.7% and 11.5%, respectively in 2016 (IPH, 2016). If compare to the prevalence reported in the NHMS 2015 (IPH, 2015), both prevalence of underweight and wasting showed declining trend but if compared with global prevalence, this national prevalence of wasting (11.5%) was much higher than the global prevalence of 6.9% (UNICEF et al., 2020a). It can be concluded that the issues of underweight, stunting and wasting are persistent among children in Malaysia. Furthermore, the rising trend of stunting prevalence in Malaysia is worrying which reflected the urge to introduce immediate and effective measures to reduce stunting in Malaysia.

A study that conducted in South East Asia using a four-country multistage cluster design, namely South East Asian Nutrition Survey (SEANUTS) (Schaafsma et al., 2013) had reported relatively lower prevalence of undernutrition in Malaysia compared to global and national prevalence. In the SEANUTS Malaysia, the findings showed a respective lower prevalence with only 5.4% of thinness and 8.4% of stunting in children aged 6 months to 12 years (Poh et al., 2013). When comparing within the four countries that involved in SEANUTS, Indonesia demonstrated the highest prevalence of stunting (39% in rural and 25% in urban) (Sandjaja et al., 2013) followed by Vietnam (17.5% in rural and 7.9% in urban) (Le Nguyen et al., 2013), Malaysia (8.8% rural and 8.3% in urban) (Poh et al., 2013) and Thailand (8.4% in rural and 4.1% in urban) (Rojroongwasinkul et al., 2013). The differences in results might be due to the differences in age group being use because in SEANUTS, a wider age group (6 months to 12 years) was used while other studies such as global reports (Development Initiatives, 2018; UNICEF et al., 2019) and NHMS (IPH, 2016) were carried out in younger age group (under five years old). Younger age children have higher risk of undernutrition compared to older age (Demissie & Worku, 2013). Furthermore, the risk of undernutrition decreases with the increase in age which might be due to the contextual improvement in term of socioeconomic and nutritional and implementation of programmes that favourably targeting older children (Astatkie, 2020).

Malaysia also reported a comparatively lower national prevalence of undernutrition than other developing countries such as India (IIPS & ICF, 2017), Africa (Boah et al., 2019; Endris et al., 2017) and Nepal (Adhikari et al., 2019). For example, in India, the national prevalence of underweight, stunting, wasting among children under age of 5 was as high as 35.7%, 38.4% and 21% (IIPS & ICF, 2017). A cross-sectional study conducted in four states of India involving 2299 children under age of 5 had asserted even higher prevalence of undernutrition (Sinha et al., 2018). The study demonstrated a prevalence of underweight ranging from 46.5% to 72.5%, stunting ranging from 37.9% to 70.7% and wasting ranging from 26.4% to 39.8% (Sinha et al., 2018).

When comparing the national prevalence with prevalence reported in abundant small-scale cross-sectional studies that conducted in Malaysia, those cross-sectional studies reported

an even lower prevalence of undernutrition, ranging from 3.5% to 18.8% (Bong et al., 2015; Mohd Nasir et al., 2012; Partap et al., 2019; Zalilah et al., 2016) than the national prevalence indicated in NHMS 2016 (IPH, 2016). The disparities occurred firstly due to the sample size being included in small-scale studies are relatively lower than national survey and secondly due to the different study setting or location being conducted in those small-scale studies. Although there are differences in reporting the prevalence of undernutrition in the country, the issue of undernutrition among children shall not be neglected due to its unfavourable consequences.

Numerous global reports, national reports or surveys and research have reported a respectively unanticipatedly high prevalence of malnutrition around the world as shown in Table 2.1. In relevant to that, goals and targets followed by effective intervention implementation have to set and execute to spur the actions toward tackling undernutrition in the world. Hence, United Nations (UN) had proposed a more comprehensive and people-centred goals and targets with ambitious vision to achieve sustainable development in economic, social and environmental in the new 2030 Agenda (United Nations, 2015). The Agenda comprises 17 SDGs and 169 targets relevant to the SDGs (United Nations, 2015). Among the 17 SDGs, the nutrition-related second goal, which is to end hunger, achieve food security and improved nutrition and promote sustainable agriculture, is emphasized and should be focused (United Nations, 2015). One of the must achievable targets in the second SDG is to end all forms of malnutrition by 2030, which includes to achieve the internationally agreed targets on stunting and wasting in children under 5 years of age by 2050 (United Nations, 2015).

Besides that, a specified set of six global nutrition targets by 2050 had specified and endorsed in the World Health Assembly Resolution 65.6 to help focusing on global community in reducing significantly the burden of malnutrition by 2050 (WHO, 2014). The global nutrition targets are also intertwined and overlapped with SDGs (Development Initiatives, 2018). The targets to be achieved by 2025 include an achievement of 40% reduction in number of children under 5 who are stunted and a reduction or maintenance of childhood wasting to be less than 5% (WHO, 2014).

Similar to other nations around the world, Malaysia adopted both global nutrition targets by 2025 and SDGs and also other references in the formulation of the National Plan of Action for Nutrition of Malaysia (NPANM) III 2016-2025 (NCCFN, 2016). In the NPANM III, targets that are set to achieve by 2025 for Malaysian children under 5 included prevalence of underweight (WAZ<-2SD) to be not more than 5% or no increase from baseline data (2.5%), prevalence of stunting (HAZ<-2SD) to be not more than 11% or no increase from baseline data (1.55%) and prevalence of wasting (WHZ<-2SD) to be not more than 5% or no increase from baseline data (1.9%) (NCCFN, 2016). However, Malaysia has only a few years left to achieve these targets by 20525. The national prevalence for all the three indicators of child undernutrition remains high, rising annually and far from targets. More dedication and commitment are urgently required to achieve the targets on time.

It is undoubtable of the great global commitment in achieving global nutrition targets by 2025 and SDGs by 2030 but the progress in the country level around the world is too slow. According to the Global Nutrition Report 2020, the number of countries that are ‘on course’ in meeting any one global target is unsatisfactory. There are only 31 countries ‘on course’, 28 countries with some progress and 17 countries showed no progress or even worsening in meeting global target of childhood stunting (Development Initiatives, 2020). For childhood wasting, there are only 40 countries are ‘on course’, 15 countries reported some progress and 24 countries demonstrated no progress or worsening in achieving the target (Development Initiatives, 2020).

For the region of South-eastern Asia, most countries (Myanmar, Philippines, Vietnam, Timor-Leste, Indonesia, Cambodia) are not in track to meet the global targets for under-five stunting and under-five wasting except for Thailand, Singapore, Lao People's Democratic Republic and Brunei Darussalam, that unable to assess due to lack of data. Thailand has shown mixed results in term of progress toward global targets which they are on course to meet under-five stunting but off course to meet under-five wasting (UNICEF et al., 2019). For Malaysia, the progress toward achieving global targets for children under-five are also not in track as other countries in South-eastern Asia (UNICEF et al., 2019). These indicate that many countries do not have progress or slow progress in solving child undernutrition issues. Despite of slow progress that is worrying, implementation to achieve global nutrition targets also faces challenges as the progress in tackling malnutrition problem is permeated with inequalities and injustice.

Inequalities are the phenomena that encompass with discrimination and marginalisation of certain group of people that arise from injustice in daily life (Development Initiatives, 2020). The inequity will cause the real health and nutrition need to be ignored, resulting in vast different nutritional outcomes such as malnutrition. In general, inequity is caused by social injustice such as discrimination due to social norms, unfairness such as suboptimal access to basic services and power imbalance or political exclusion such as less chances to be allocated with certain resources due to less political power (Development Initiatives, 2020). Hence, inequalities in the context of nutrition can be illustrated in two levels which begin at basic level and extend to underlying level which embraces ample of determinants (Development Initiatives, 2020).

At the basic level, nutrition inequalities happened due to some basic determinants such as socioeconomic and political influence, human potential and social position. In this level, the three determinants influence each other, resulting in diverse inequalities in nutritional outcomes (Development Initiatives, 2020). For example, certain group of people might experience discrimination due to social norm or governance interest which further restrain their potential to share equal chances of education, employment, economic and social services and later affect their social position such as income level. Then, the different social characteristics such as sex, age, ethnicity, including the wealth status affect both the human potential and the socioeconomic and political setting. This process of inequity will be continued and extended to the underlying level that build up the daily lives of people.

At the underlying level, the two interrelated determinants are daily life and norms such as behaviour, practices and health norms and environments such as food, healthcare and living environment (Development Initiatives, 2020). In this level, nutrition inequity results from the way people live their daily life and interrelated with some environmental factors such as availability to healthy food, sanitation, healthcare services and residency location (Development Initiatives, 2020). For example, people who live in rural areas might find that they lack of access to healthy food and less opportunity to seek heath advices which shape their eating behaviours and eating practices and later might lead to child malnutrition.

The inequalities in malnutrition are manifested in different levels from individual to country level. Basically, the inequalities in malnutrition can be described in term of socio-demographic such as sex, age, education level and wealth status and environmental such as location. Globally, there are small differences by sex for both the prevalence of stunting (33.5% in boys and 31.2% in girls) and wasting (12.8% in boys and 11.5% in girls) (Development Initiatives, 2020). For the prevalence of stunting, the largest disparities are manifested in the wealth status in which higher percentage of stunting among poorest household (43.6%) than richer household (18.6%) (Development Initiatives, 2020). The prevalence of stunting is also higher in household with less educated mother (39.2%) than more educated mother (24%)

(Development Initiatives, 2020). For wasting, the prevalence demonstrated not much differences between urban (11.4%) and rural areas (12.4%) and between less educated (12.9%) and more educated mother (11.2%). The only inequalities found is the wealth gap, in which wasting is more prevalent among the poorest households (14.1%) compared to the richest households (10.0%) (Development Initiatives, 2020).

In Malaysia, the largest inequalities can be seen in the prevalence of stunting based on data obtained from NHMS 2016. There are small differences in the prevalence of stunting between urban (19.23%) and rural areas (23.34%), boys (22.2%) and girls (19.2%) and age group (23.3% for 6-11 months and 19.2% for 48-59 months), except for mother's educational level (28.7% in mother who received no education and 18.6% in mother who receive education higher than secondary) and household income (29.8% for income less than RM1000 and 17.4% for income more than RM 5000) (IPH, 2016). For the prevalence of wasting, all disparities by location (11.4% in urban and 11.7% in rural), sex (13.3% for boys and 9.7% for girls), age group (10.8% for 6-11 months and 13.6% 48-59 months), mother's education level (8.5% for not educated other and 9.3% for more educated mother) and household income (9.4% for less than RM1000 and 10.2% for more than RM 5000) are relatively small (IPH, 2016).

Although Malaysia shows quite different disparities of nutritional outcomes from the global report, stakeholders should consider that the inequalities are actually existed and many vulnerable groups are being marginalized with diminished right to share equal resources. Recognising that there are few years left for each country worldwide to achieve global targets by 2025, there is an urge to exaggerate the efforts and actions in tackling the persistent undernutrition problem. The implementation of strategies such as intervention, prevention program and political decision should begin to employ concept of equity-sensitive by involving vulnerable population who unevenly burdened by malnutrition and being ignored previously, particularly the urban poor families.

In conclusion, undernutrition is prevailing and prevalent across countries at high levels. Although abundant of strategies have been implemented, the process and effectiveness of those strategies are progressing too slow with unstratified yield. Recently, the attention is being draw to the nutrition equity. The diverse characteristics of human might result in divergent nutritional outcomes from obesity to underweight and in the process of constructing actions to unravel the consequences caused by the nutritional outcomes, the inequalities and injustice may occur, increasing the gap between the most advantaged and disadvantaged people. This emphasized the importance of coverage of nutrition care to all people disregard of any characteristics. Each individual in this world deserves the right to enjoy the right to obtain equal health and nutrition care. The shift of attention might also hope to invoke and accelerate the progress toward decreasing undernutrition in children.

2.1.2 Factor associated with child undernutrition

The Conceptual Framework of the Determinants of Maternal and Child Nutrition by UNICEF (2019) is illustrated in Figure 2.1. According to the conceptual framework, the nutritional outcomes of children is affected immediately by the diet intake and care for them while the diet and care are influenced by the food being fed to them, the feeding practices and environment. The food, feeding practices and environment are further affected by resources such as environmental, financial, social and human resources, governance and norms which include political, financial, multisectoral commitments (UNICEF, 2019).

The common causes responsible for all forms of malnutrition across the world are suboptimal feeding, which include inadequate breastfeeding and poor diet (Development Initiatives, 2018). These causes are mainly rooted in three aspects which are biological aspect, environmental aspect and behavioural aspect which are interrelated and directly or indirectly

diminished capability to obtain normal yet healthy diet (Hirani, 2012). Plenty of studies have concluded that biological, behavioural and environmental factors are associated with child undernutrition (Adhikari et al., 2019; Boah et al., 2019; Hien & Hoa, 2009; Hirani, 2012; Kavosi et al., 2014; Sinha et al., 2018; Tiwari et al., 2014).

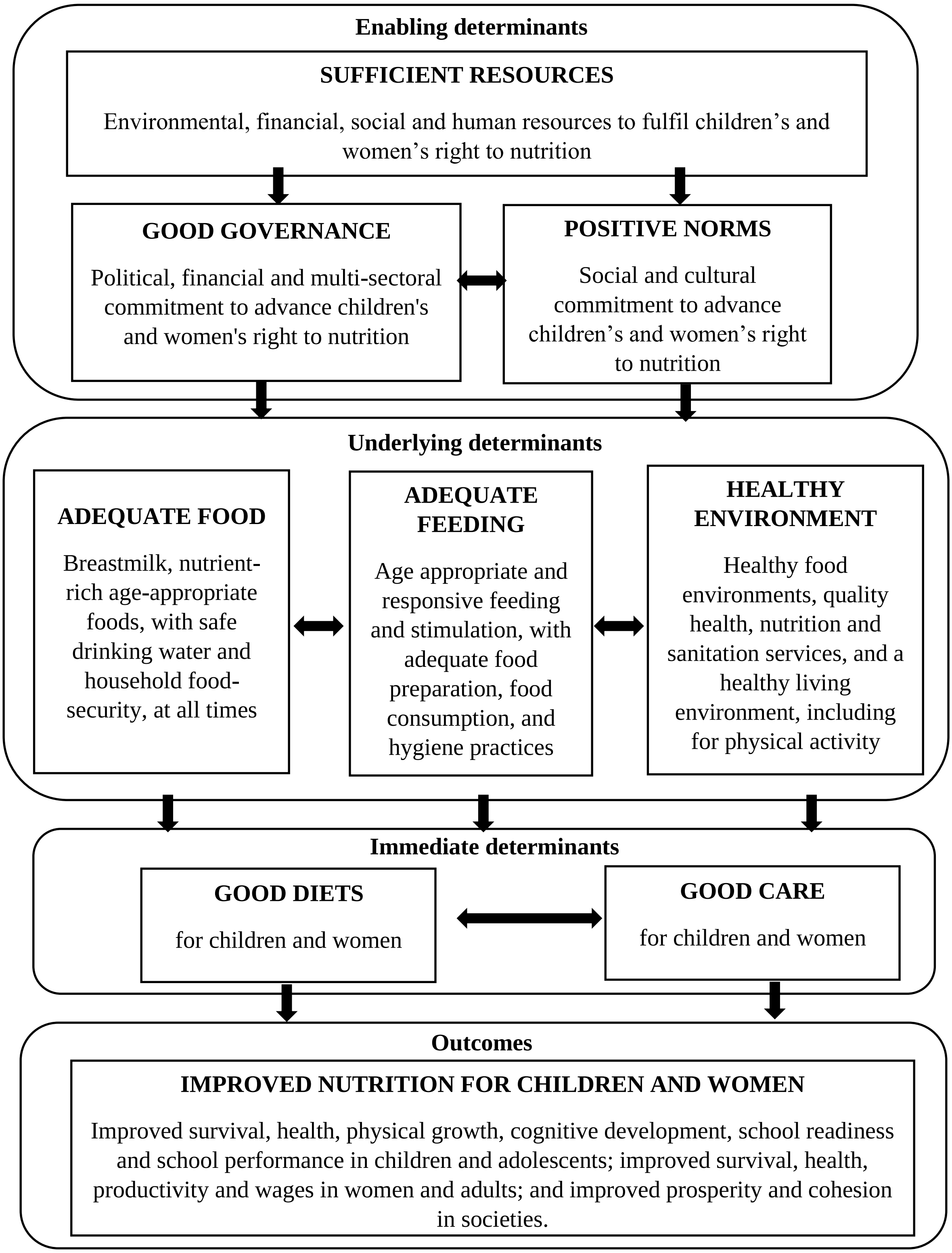

Figure 2.1: Conceptual Framework of the Determinants of Maternal and Child Nutrition Source: UNICEF (2019)

2.1.2.1 Biological factors

From biological perspective, age and sex of children are associated with undernutrition. Several previous studies have shown significant findings in the association between child's age and undernutrition (Adhikari et al., 2019; Boah et al., 2019; Demissie & Worku, 2013). For instance, a longitudinal study, the Young Lives study, conducted in Ethiopia concluded that the chances of undernutrition decreases with increasing in age (Astatkie, 2020). The possible reasons include the dependency of younger children on parental feeding to obtain food and the implementation of interventional programs that always target older children instead of younger children (Astatkie, 2020). However, another study conducted in Ethiopia has demonstrated different findings, in which younger children were at higher risk of wasting and older children were more likely to be stunted and overweight, arguing that the susceptibility of older children was rooted in decreasing in care and attention from parents when increasing in age (Demissie & Worku, 2013).

Vast studies have reported higher risk of undernutrition in male children than in female children (Boah et al., 2019; Demissie & Worku, 2013; Kavosi et al., 2014; Sinha et al., 2018), which also consistent with the recent global report (Development Initiatives, 2020) that asserted higher prevalence of undernutrition in boys (33.5% for stunting and 12.8% for wasting) than girls (31.2% for stunting and 11.5% for wasting). The possible explanations including the gender bias in child feeding by parent (Bork & Diallo, 2017), the use of different global growth references for boys and girls (Wamani et al., 2007) and the different biological mechanism between boys and girls such as boys are more susceptible to infectious disease than girls (Bork & Diallo, 2017; Wamani et al., 2007) have been proposed. Bork and Diallo (2017) further explained that young boys are more likely to show poorer nutritional status than girls because they are at higher chances of practicing early complementary feeding (particularly at age of 2 to 3 months), possibly due to maternal perception of breast milk insufficient, and those children who grew up without exclusively breastfeeding are more susceptible to infectious disease.

However, there are many disagreements being claimed alongside with those possible explanations. A study that utilized data from the Young Lives cohort study across Ethiopia, India, Peru and Vietnam had justified that the evidences and sources provided in previous studies that discussed on disadvantages of boys leading to poorer nutritional outcomes were indeed ambiguous and inconclusive although this study demonstrated a result that boys were more likely to have lower nutritional outcome than girls (Dercon & Singh, 2013). In contrast, another study aiming to determine the global trends of gender discrimination in term of child care and health had concluded that girls were more likely to have poorer nutritional outcome than boys because girls were discriminated in few dimensions such as immunisation, household allocation of food, health seeking behaviours and healthcare expenditure (Khera et al., 2014). Yet, the explanations on gender differences in health seeking attitude practices is opposed by the study of Garenne (2003). Garenne (2003) found no disparities in health seeking behaviour between boys and girls.

Despite children biological factors, maternal biological factors such as nutritional status of mothers including weight, height and BMI status are significantly associated with nutritional status of children. Few previous studies examining this association have consistently reported that mothers with poor nutritional status (underweight, short or low BMI) are more likely to bear children with poorer nutritional status (stunting, underweight and wasting) (Corsi et al., 2016; Hien & Hoa, 2009; Negash et al., 2015; Santos Felisbino-Mendes et al., 2014; Senbanjo et al., 2013; Sinha et al., 2018).

A cross-sectional study conducted by Subramanian et al. (2010) presented that for each increase in one unit of BMI in mother, there was a lower relative risk (RR) of undernutrition in children (underweight RR: 0.957, stunting RR: 0.985 and wasting RR: 0.941). The same

study also suggested that there is an intergenerational effect between mother and children. This effect could be one of the possible explanations of the influences of mother nutritional status on child nutritional status (Subramanian et al., 2010). The intergenerational effect is referred as the effects of undernutrition that pass down from one generation to another generation when mothers who grow up to be underweight, stunted or wasting, tend to raise children who have same compromised nutritional status as them, perpetuating a vicious cycle of malnutrition (Rannan-Eliya et al., 2013).

Another possible explanation of the association between maternal nutritional status and child nutritional status is due to environmental factor. In general, mother is the primary caregiver of children and the food environment of children is greatly subjected to maternal influences. When mother and children live in the same household with shared environment, factors such as lack of food access, inadequate diet and unbalance nutrition will affect both the mother and children (Gewa & Yandell, 2012). Hence, same environment with identical deprivations might expose equal chances of nutrition deficiency among them, compromising their nutritional status simultaneously. This environmental factor also supported the explanation of the intergenerational effect as living environment is a major driver to shape living and feeding style or practices, which might pass from one generation to the next (Black et al., 2013).

Other biological factors that would be related to malnutrition in young children including low birth weight (Adhikari et al., 2019; Boah et al., 2019; Hien & Hoa, 2009; Tiwari et al., 2014) and chronic or infectious diseases (UNICEF, 2019) such as anaemia (Adhikari et al., 2019), congenital abnormalities (Hirani, 2012), acute respiratory infection (Sinha et al., 2018), and diarrhoea (Ayana et al., 2015; Sinha et al., 2018). The disease-related factors which able to further deteriorate the nutritional status of children, perpetuating the vicious cycle of malnutrition.

2.1.2.2 Behavioural factors

At each stage of life, people have different nutritional needs, eating behaviours and dietary preferences. Younger children (0 to 24 months) are mainly depend on their parents for daily diet via breastfeeding and complementary feeding and they are mainly eating at home with their parents, siblings or caregivers (UNICEF, 2019). During this period, children are more subjected to poor diet when inappropriate parental feeding practices are applied. Besides that, children are fussy eaters who change taste and food preferences frequently, in which these may force their parents to feed them whatever they prefer without consideration of nutrition balance (UNICEF, 2019). Therefore, age-appropriate child feeding practices seem to be vital and critical to nutrition status of children.

Several previous studies have presented that parental feeding practices are significantly associated with high prevalence of undernutrition in children (Irarrázaval et al., 2018; Menon et al., 2015; Mwase et al., 2016; Nowicka et al., 2014). The poor and unhealthy parental feeding practices that are significantly associated with child undernutrition include prolonged breastfeeding (Tiwari et al., 2014), absence of exclusive breastfeeding (Ayana et al., 2015; Hien & Hoa, 2009), early cessation of breastfeeding (Hirani, 2012), inappropriate weaning, late initiation of complementary feeding (Pravana et al., 2017), use of formula feeding (Hirani, 2012), and bottle feeding (Pravana et al., 2017).

During toddlerhood (2 to 4 years), children might have more autonomy on their diet compared to younger children, especially when they start to eat in kindergarten or nurseries (UNICEF, 2019). When they start to eat outside from home, they also start to be affected by outside food environment such as food advertisement that overwhelmed with unhealthy snack and processed foods (UNICEF, 2019). The chances of consuming inadequate diet or poor-quality diet such as excess calorie, sugar, salt intake, less vitamin and minerals intake and over

consumption of ultra-processed foods are getting higher among them, leading them to malnutrition. At this age, the influence from adults such as parents, caregivers and also siblings are vital in acting as positive role model in demonstrating to them the appropriate way of eating adequately and healthily (UNICEF, 2019). Hence, parent's feeding practices may directly influence the children's eating behaviours and their food choices.

Parenting style is interrelated with feeding practices which acts as a driver that constructs the behaviour and emotional of children. Parenting style can be elucidated in two dimensions, which are demandingness (the extent of parental control) and responsiveness (the readiness and acceptance according to children's responses and needs) (Baumrind, 1966). Baumrind (1966) had conceptualized the possible parenting styles in three categories, including authoritative (high demandingness and high responsiveness), authoritarian (high demandingness and low responsiveness) and permissive (low demandingness and high responsiveness). In later years, these concepts had been extended and a new category of neglectful parenting style (low demandingness and low responsiveness) had been considered (Maccoby & Martin, 1983), resulting the formation of the four classical categories of parenting styles.

In 2005, Hughes et al. (2005) had developed and incorporated the measures of parenting styles in the context of child-feeding, forming four classifications of feeding styles (authoritative, authoritarian, indulgent and uninvolved) as shown in Figure 2.2. Their study showed a significant difference of child BMI-for-age z-score (BAZ) between indulgent and authoritarian feeding styles ($p<0.01$), concluding that parents who applied authoritarian feeding styles tend to have children with lower BAZ (Hughes et al., 2005). Hughes et al. (2005) explained that authoritarian feeding style is denoted as high demandingness and low responsiveness and it was highly associated with feeding practices such as restriction on certain food, pressure to eat certain food and physical punishment when children refused to eat certain food.

On the other hand, maternal feeding practices are referred as strategies in term of behavioural aspects that use by mothers to affect food consumption of their children (Gregory et al., 2010). The practices that are commonly described in studies are restriction of eating, pressure to eat and monitoring when eating (McPhie et al., 2014). A systematic review carried out by McPhie et al. (2014) had revealed significant association between maternal parenting and feeding practices. For example, mothers who tend to use more demandingness parenting styles such as permissive style were more likely to develop practices such as restricted children to eat or asserted more pressure on children to eat certain food (McPhie et al., 2014). However, the findings might subject to uncertainty because diverse measures of parenting style and feeding practices had been used in previous studies.

Ample of previous studies had reported a positive association between maternal feeding practices that restricted children to eat certain food and BMI of children by explaining that parental restriction on certain food might increase the curiousness and desires of children to consume that certain food, leading to overconsumption (Birch et al., 2001; Clark et al., 2007; Crouch et al., 2007; Johannsen et al., 2006; McPhie et al., 2014). However, the National Longitudinal Survey of Youth (NLSY) in the United State demonstrated different findings (Faith et al., 2003). The study reported that maternal feeding practices were not related to child BAZ, suggesting that exaggerated restriction by parent on the food choices of children decreased the compliance of children to food and may result in lower BAZ (Faith et al., 2003).

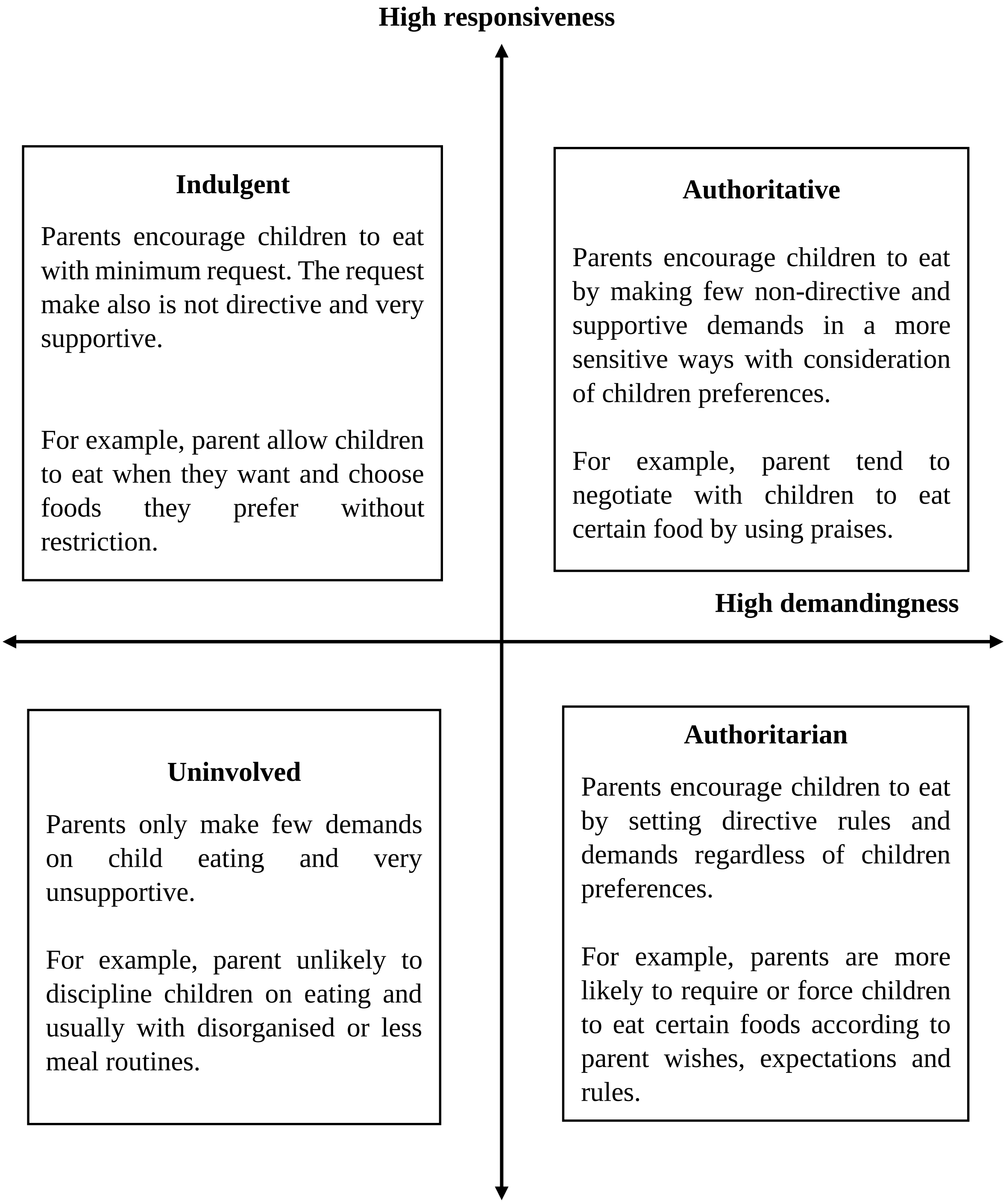

Figure 2.2: Parental feeding styles and examples of practices

Source: Hughes et al. (2005)

Furthermore, feeding practice that pressure children to eat more or certain food was significantly associated with lower weight status in children (Crouch et al., 2007; Fisher et al., 2002; Galloway et al., 2005; Mais et al., 2017). The mechanism behind the association remains unclear but assumptions concluded from previous studies include high maternal pressure to eat reduced children intake of average calories, energy-dense food and high-fat foods, perception of parent to control by giving pressure to eat when children have small appetite (Lee & Keller, 2012), the higher probabilities of pickiness in children due to high pressure of eating and the concern of parent on diet quantity instead of quality when children are picky eaters (Shloim et al., 2015; Vaitkevičiūtė & Petrauskienė, 2019).

On the other hand, for the practice of monitoring when eating, McPhie et al. (2014) concluded that this practice was less studied and there were only six cross-sectional studies reported of no relationship between maternal monitoring and food intake or weight status of children. In contrast, a previous study involving 57 families who enrolled in the Longitudinal Infant Growth Study of Unites States has shown different results, in which a negative relationship was found between monitoring practice and child BAZ (Faith et al., 2004). The difference in findings might be due to the relationship only found in children grew up in thin families who were at lower risk of obesity. Faith et al. (2004) explained that parents of families that predisposed to thinness were more likely to monitor eating practices of thinner children due to the fear of insufficient food consumption and slower growth. The way a family to procure and prepare food and how the children are being fed and supervised is vital in shaping eating behaviours and food preferences of children (UNICEF, 2019). Each child shows different and diverse eating behaviours and the most common behaviour that perceived by parents as troublesome is pickiness (Wardle et al., 2001). Wardle et al. (2001) classified child eating behaviours as food approach behaviours and food avoidance behaviours. Food approach behaviours are referred as positive behaviours when consuming foods which include food responsiveness, food enjoyment, emotional overeating and desire to drink, while food avoidance behaviours are denoted as negative eating behaviours which include satiety responsiveness, slowness in eating, emotional undereating and food fussiness (Wardle et al., 2001).

Substantial amount of studies showed consistent results that children who demonstrated food avoidance behaviours were associated with underweight and also significantly related to maternal feeding practices (Bergmeier et al., 2015; Gregory et al., 2010; Haycraft et al., 2017; Quah et al., 2019; Yuan et al., 2019). For example, parents who used negative verbalization and fail to encourage their children to eat when feeding are more likely to cause lower food acceptance among children. Their children tend to show more food avoidance behaviours such as food fussiness which decrease the variety and quantity of food being consumed and lead to higher risk of undernutrition (Bentley et al., 2011). Hence, recognising the possible relationships between feeding practices, child eating behaviours and nutritional status, the level of knowledge of mother seems to be vital for them to perform the appropriate feeding practices to cultivate healthy eating behaviours in their children.

Nutritional knowledge of parents is closely related to their education level. Numerous studies have shown that children with less educated mothers are more likely to be undernourished (Ayana et al., 2015; Boah et al., 2019; Demissie & Worku, 2013; Irarrázaval et al., 2018; Kavosi et al., 2014; Tasnim, 2018). Less educated mothers tend to have lack of adequate health and nutrition knowledge which hinder them to seek for advices, resulting in inactively engaging in health seeking behaviour and lack of ability to prevent and protect themselves and children from malnutrition (Astatkie, 2020).

Besides that, behavioural factors are also interrelated with some socio-economic and cultural factors such as food taboos, gender bias, and social norms. For instance, a systematic

review conducted by Hirani (2012) on determinants of malnutrition in young Pakistani children revealed that the most common socio-cultural factors contributing to high prevalence of malnutrition in Pakistan was gender inequality that caused uneven food distribution in a household, in which boy child is more preferred to be given more food in term of quality and quantity as compared to girl.

2.1.2.3 Environmental factors

Child undernutrition can also be associated with environmental factors such as climate change, availability of food, globalisation (UNICEF, 2019), access to clean water, sanitation and hygiene (Development Initiatives, 2018) and poor water supply in urban areas (Kavosi et al., 2014). The shock caused by climate change such as flooding may disrupt the food systems which decrease the food production, causing lower consumption of food. Besides that, children are also at high risk of infectious diseases which further cause undernutrition among them (UNICEF, 2019). The food availability which determines the access to healthy food is more related to the food environments such as marketing and remoteness of residency (UNICEF, 2019). For example, children who live in rural or remote area are more susceptible to malnutrition as they might not reach out for food retail or services that supply healthy foods and also quality health care services that promote healthy eating (Kavosi et al., 2014).

Globalization has increased the flow of products, invoke the change of information and technologies across the world and has altered the food systems entirely, affecting the food environment and eating behaviour of people (UNICEF, 2019). The benefits of globalisation include the widely accessible to diverse health food and increase food choices but on the other hand, it has struck the rapid arise of fast food and ultra-processed food markets (UNICEF, 2019). The arising of fast food and ultra-processed food markets and the convenience of parents to access those foods has changed the demand of consumer and food chain supply, creating huge challenges in promoting healthy eating in children and parents (UNICEF, 2019).

Water pollution and poor sanitary conditions are also associated with nutritional status of children. Such conditions may increase the spread of unhygienic practices and later might cause repeated intestinal infections among children (Ijarotimi, 2013; Kinyoki et al., 2015). Some infectious diseases especially those related to intestinal might diminish the potential of children to consume and absorb adequate nutrients and subsequently lead to weight loss (Tette et al., 2015). Such unnecessary and significant weight loss result in undernutrition which make them weaker and vulnerable to diseases.

Another environmental factor that is also contributed to malnutrition is the condition of a household which include the household size. Numerous studies have shown that children from larger family are more likely to be undernourished (Adhikari et al., 2019; Ayana et al., 2015; Ersino et al., 2018; Hien & Hoa, 2009; Kavosi et al., 2014). The possible explanation is that children who grow up in a large family usually indicate the fact that they are living in overcrowded housing environment. In such conditions, they might have to compromise quality and quantity of their food intake due to uneven food distribution in the family, especially in poor household (Hirani, 2012). When children eat less, they are not consuming adequate amount of nutritious food which subjected them to undernutrition.

Socioeconomic status of a family is also found to be associated with malnutrition in children. Children who grow up in a household with poor socioeconomic status are usually exposed to higher risk of poor growth and development due to limited access to adequate food (Adhikari et al., 2019; Demissie & Worku, 2013; Kavosi et al., 2014; Shariff et al., 2015; Tasnim, 2018; Tiwari et al., 2014). It seems that poverty is likely to be a detrimental factor that affect children in a family to be undernourished. When it comes to poverty, it brings out another interrelated issue, which is gaining attraction worldwide recently, namely food insecurity.

Food insecurity is a dynamic and multidimensional phenomenon involving the lack of food and nutrition security that exerts nutritional impacts. Food insecurity can be classified into two categories by using a new developed scale in 2018, called Food Insecurity Experience Scale (FIES). The first category is moderate food insecurity which refers to compromising on food quantity, quality and variety with the likelihood of skipping meals. For the second category, it refers to severe food insecurity, which is defined as the experience of running out of food and consuming no food for more than one day. In the context of food insecurity, there are four essential dimensions which are food availability, access, utilization and stability which significantly associated with malnutrition (FAO, IFAD, UNICEF, WFP & WHO, 2020). According to FAO et al. (2018), there are multiple pathways to explain how food insecurity may result in multiple forms of malnutrition, as shown in Figure 2.3.

There was association between food insecurity and undernutrition (Abdurahman et al., 2016; Ali et al., 2013; Chandrasekhar et al., 2017; Motbainor et al., 2015; Mutisya et al., 2015; Psaki et al., 2012; Sarma et al., 2017). However, studies have demonstrated mixed findings, either with significant association between food insecurity and stunting and underweight but no association between food insecurity and wasting or vice versa (Ali Naser Ihab et al., 2015; Mulu & Mengistie, 2017; Saaka & Osman, 2013). This is supported by a review conducted by Maitra (2018) which also reported mixed results in term of the association between food insecurity and child undernutrition (stunting and wasting only). Among 30 studies that evaluated the association between food insecurity and stunting in children, majority studies (15 cross-sectional studies and one longitudinal study) reported positive association, one cross-sectional study showed mixed results and four cross-sectional studies showed no association among under-five children (Maitra, 2018). For wasting among under-five children, within 15 cross-sectional studies reviewed, majority (12 studies) demonstrated no association and only three studies reported positive association (Maitra, 2018). However, the difference in results might be explained by the limited number of studies had been reviewed, the different study design had been considered and analysed together and different age groups had been included. The mechanism to explain the possible association between food insecurity and chid stunting is easily understood. Children who live in households that unable to prepare adequate diet with sufficient amount of food, good diet quality and balance nutrition are more prone to compromise their diet which subsequently might affect their nutritional status (Chandrasekhar et al., 2017; Ihab et al., 2015; Zalilah & Tham, 2002). A cross-sectional study conducted in Kelantan, Malaysia that involved 223 households had revealed that households who experienced food insecurity had lower animal protein, fruits and vegetables consumption (Ihab et al., 2015). In term of the association between food insecurity and wasting, most studies reported of no association (Betebo et al., 2017; Naser et al., 2014; Singh et al., 2014), which could be explained that wasting is acute undernutrition which the symptoms might have been disappeared when assessing food security status.

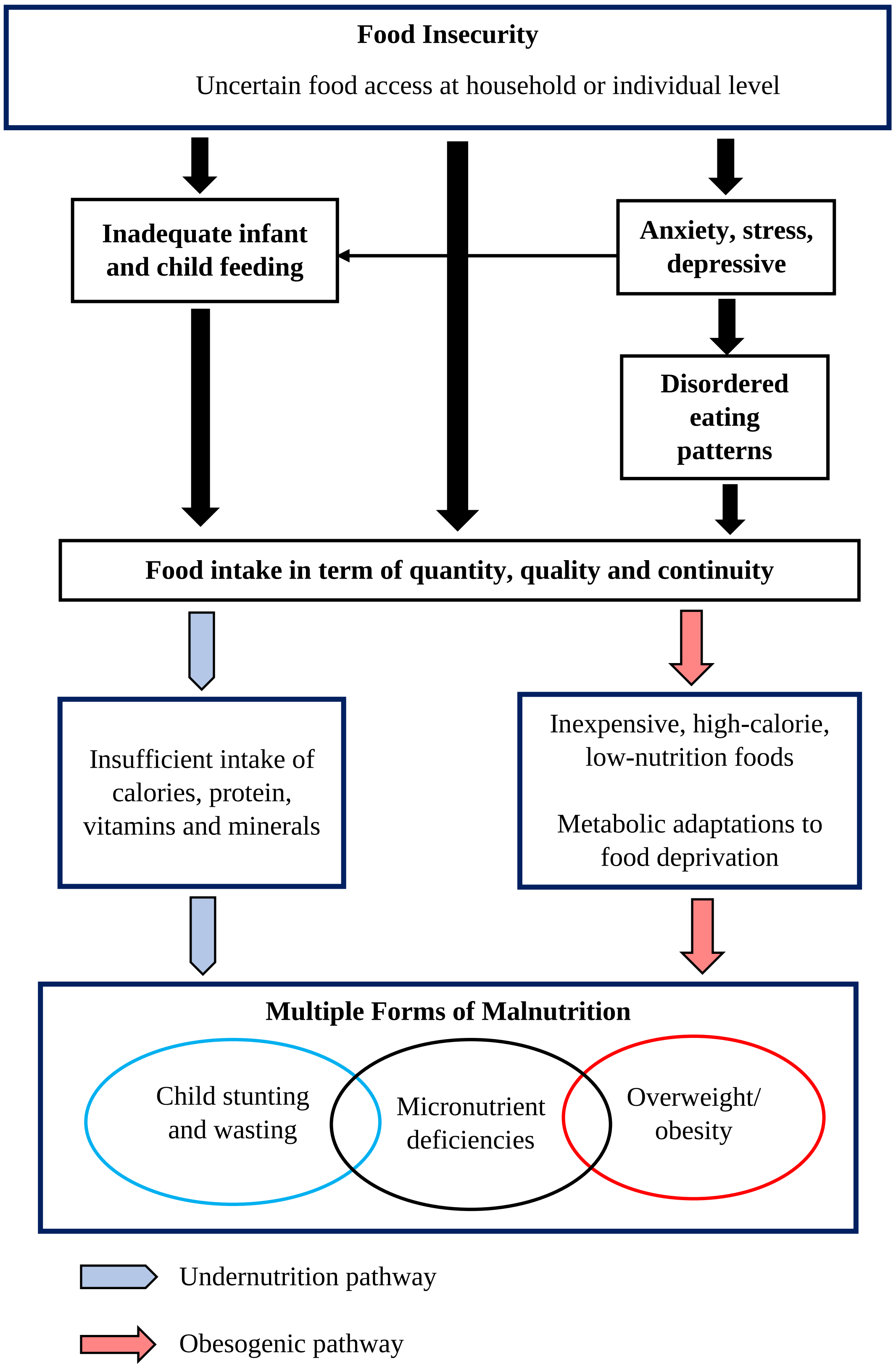

Figure 2.3: Multiple pathways revealing association between food insecurity and nutritional status

Source: FAO et al. (2018)

2.1.3 Consequences of undernutrition

The unfavourable effect of undernutrition in the early stage of life is shouldered by children around the world. The effects of undernutrition are even more devastating in young children and in more severe cases such as severe acute malnutrition. According to the Global Nutrition Report (2018), about 45% of deaths cases among children under five are related to malnutrition in both low and middle-income countries. In general, undernourished children suffer from poor growth and development, poor cognition and school performance, recurrent infections and diseases and poor earning in adulthood (UNICEF, 2019). Furthermore, the undesirable economic impact of undernutrition in children under age of 5 seems to affect different level from global to individual level (UNICEF, 2019).

Undernourished children tend to consume less food in term of both quantity and quality that is necessary for optimal growth and development (Development Initiatives, 2018). Inadequate nutrient consumption usually accompanies with loss of body fat and muscle which lead to retarded growth that in clinical setting these terms are more referred as skeletal muscle atrophy, kwashiorkor and marasmus (Shetty, 2006). Besides that, undernutrition in early life might have long-term effects on brain, neurology and intellectual development of children (Cusick & Georgieff, 2016). The simple theory behind is that brain is the most metabolically active organ that rely on diet for continuous glucose supply as energy and inadequate diet caused incomplete but lasting changes in brain structure and intellectual function (Benton, 2010).

Nutritional deficiencies in early life not only affect the brain development but also affect other domains such as cognitive, behaviour, socio-emotional skills (Prado & Dewey, 2014), motor, visual, auditory and social development, which might last through school-age and adulthood (De & Chattopadhyay, 2019). The developmental delay subsequently lead to motor disabilities such as cerebral palsy, mental retardation, impairment in visual and hearing, speech delay and learning disabilities (De & Chattopadhyay, 2019), which greatly influence the ability to learn and may result in poor educational outcomes (FAO et al., 2018). Malnourished children have a tendency to spend less time in school due to impaired brain and cognitive development, less school readiness and illness or simply due to delay in starting school and repeating grades (UNICEF, 2019).

Undernourished children especially those who have specific micronutrient deficiencies tend to compromise their ability to fully develop immune system due to poor growth, exposing them to periodic infections and recurrent diseases in adulthood (Joshi, 2018). Besides that, the likelihood to develop chronic diseases or non-communicable diseases in adulthood such as overweight, hypertension and cardiovascular disease also increase among undernourished children (UNICEF, 2015). Hence, it can be concluded that undernutrition in early year of life can increase the morbidity and mortality in children (Boah et al., 2019), in which the intergeneration malnutrition effect is unable to be eliminated in a family. Indeed, the effect will pass from generation to generation, in which the offspring of parents who had experienced malnutrition in childhood are also at high risk of malnutrition and suffer from the consequences (Martorell & Zongrone, 2012; Waber et al., 2018).

Vast amount of previous studies have indicated that people who suffered from undernutrition in early life are at greater tendency to have poorer intellectual quotient (IQ) levels (Galler, Bryce, Waber, et al., 2012; Ghazi et al., 2012; Schoenmaker et al., 2015), poorer school achievements (El Hioui et al., 2016; Prado & Dewey, 2014; Wisniewski, 2010), engaging in behavioural problems such as more anxiety, shyness, less sociable, less intellectual curiosity, greater suspiciousness of others and a low self-efficacy (Galler et al., 2013) and some attention deficit problems (Galler, Bryce, Zichlin, et al., 2012) in adulthood. Furthermore, undernourished children may experience late schooling and subsequently result in late entry

into labour force due to growth and developmental delay (UNICEF, 2019). This might also indicate that they are at high risk of reduced productivity and also earnings in later life due to lower working performance (Galler, Bryce, Waber, et al., 2012; Prado & Dewey, 2014; UNICEF, 2015).

A study conducted by Fink et al. (2016) which included 137 developing countries aimed to quantify the monetary cost related to growth faltering. The findings showed that globally, the total economic costs due to growth faltering was as high as US$176.8 billion per birth cohort and South Asia recorded the highest economic costs (US$46.6 billion), followed by Latin America (US$44.7) and sub-Saharan Africa (US$34.2 billion). For South-eastern Asia, the estimated total income lost associated with growth faltering was lower (US$18 billion). At individual level, the estimated lifetime earnings losses due to growth faltering in childhood ranged from US$300 to US$30,000. The study also concluded that undernourished children of developing countries were more likely to loss 0.5 year of educational accomplishment causing a global economic loss of $176.7 billion and an average lifetime incomes loss of US$1400 per child (Fink et al., 2016).

In addition, the economic effects of child undernutrition can be evaluated via the possible influence of change in anthropometric measurements on change in earnings. A systematic review conducted by McGovern et al. (2017) reported a significant association between increase in height and increase in lifetime earnings. Findings showed that the largest extent of reported percentage increase in lifetime wages (25% to 46%) associated with one-centimetre increase in height were found in interventional studies (7 studies with randomized design), followed by quasi-experimental studies (10 studies reported a percentage of increase in wages that ranged from 4% to 6%) (McGovern et al., 2017). This indicated that undernourished children who are shorter due to inadequate growth are more likely to have lower productivity and earnings with a greater possibility of poverty during adulthood.

The economic influences of undernutrition can also be extended from individual level to country level. The simple reason is that an individual who experienced child undernutrition is more prone to have poor growth and development which could affect working performance and subsequently undermine the progress of countries to develop the human capital (UNICEF, 2019). Human capital refers to the collective levels of education, training, skills and health, which is linked to the economics of a country (UNICEF, 2019). Mary (2018) observed a dataset of 74 developing countries from 1984 to 2014 with the intention to examine the effect of stunting on Gross Domestic Product (GDP) per capita, had estimated that an increase in GDP per capita by 10% might reduce child stunting prevalence by 2.7%. Each percentage increase in stunting prevalence may induce a decrease in GDP per capita by 0.4% (Mary, 2018). Hence, these findings suggested that undernutrition during childhood might exert lifelong economic effects which greatly affect the monetary resources at both individual and country levels. This will subsequently result in poverty which invoke a viscous cycle as poverty might further lead to undernutrition (UNICEF, 2019).In conclusion, child undernutrition is a persistent problem that affects almost all countries worldwide with alarmingly high prevalence. Undernourished children who manifested as underweight, stunting and wasting are vulnerable and suffered more than expected. To understand undernutrition in children, the enabling, underlying and immediate determinants should be considered clearly before progressing to next step of intervention. Undernourished children also shoulder with heavy burden and consequences of undernutrition such as poor development, poor school performance and recurrent infections and diseases. Hence, interventions targeting those children should be carried out and scaling-out in a more effective and innovative way to reduce undernutrition incidences in order to meet the global nutrition targets.

2.2 Urban Poor

2.2.1 Urbanisation

Urbanisation is a multifaceted phenomenon that can be defined in term of demographic, ecological, sociological and economic (Cobbinah et al., 2015a). In general, urbanisation involves the transformation of environment from formerly rural into urban settlements and the change of spatial distribution of a population from rural to urban areas (United Nations, 2019). It is common that urbanisation occurs alongside with drastic demographic and social structure changes in both rural and urban areas in the way that it often caused altering in the dominant occupations, lifestyle, culture and behaviour in both areas (United Nations, 2019). With all the changes that urbanisation invoked, the foremost consequence is a greater share of urban residents with an increase in the number of land and population size of urban residents (Tacoli et al., 2015). Although urbanisation is causing increased in urban population, the concept of urbanisation is very much different from urban population growth because the total population is also growing at the same time (United Nations, 2019).

The degree of urbanisation in an area is predominantly described as the percentage of residents in urban area but distinctive criteria have been used by different countries in measuring the level of urbanisation (United Nations, 2019). Indeed, there is no definite measurement criteria set to be accepted universally in measuring level of urbanisation (Alirol et al., 2011). Basically, the criteria in measuring the degree of urbanisation can be divided into basic administrative boundaries such as residing in capital city, population measures or demographic characteristics such as population size or density, economic characteristics such as economic activities, and functional characteristics such as existence of water-supply systems (United Nations, 2019). According to the United Nations (2019), over half of the countries or areas in the world (51.9%) used administrative characteristics in distinguishing urban and rural areas, either solely or with combination with other criteria.

According to the United Nations (2019), in the last few decades, the number of urban population around the world was rising tremendously from an estimated 0.8 billion in 1950 to an estimated 4.2 billion in 2018. From 1950 to 2018, the urban population share was increasing from 30% to 55%, indicating that more than half of the world's population residing in cities in 2018. Within the same period, the average annual rate of urbanisation was estimated at 2.54% with 0.92% increase per year in average. The process of urbanisation is never ending and is expected to continue for decades but in a slower pace. Urban population of the world is estimated to reach 5 billion in 2028 and further to 6 billion in 2041. By 2050, it is postulated that 68% of the world population will resident in urban areas with a number of 6.7 billion population. In Asia, the urban population has increased for about nine-fold, from 246 million to 2.3 billion between the period of 1950 to 2018. It was estimated that almost half of Asian population are living in urban areas in 2018 and by 2050, the forecast percentage of urban population in Asia will be as high as 66% (United Nations, 2019).

Malaysia, similar to other countries in Asia and other upper-middle income countries, is also urbanized rapidly in the few decades (United Nations, 2019). The level of urbanisation in Malaysia is deduced by its own definitions. According to the Population and Housing Census of Malaysia 2010, urban areas in Malaysia is defined as any gazetted area, including the attached built-up areas, with a total population of 10,000 or more. Built-up areas referred to any areas adjoining to a gazetted area which had at least 60% of their population (aged 15 years old and over) involved in non-agricultural activities (Department of Statistics Malaysia, 2010). The criteria to classify an area as urban area in Malaysia are also based on statistical boundaries and include any special development area which is not gazetted, able to be separated from the gazetted area or built-up area of more than five kilometre and with a population of at least

10,000 with 60% of the residents worked in non-agricultural activities (Department of Statistics Malaysia, 2010).

The total population growth in Malaysia has increased for almost three-fold from 13.7 million in 1980 to 32.68 million in 2019 (Department of Statistic Malaysia, 2020). If compared to estimation population number in fourth quarter of 2018 (32.48 million), the estimated Malaysia's population in the fourth quarter of 2019 (32.68 million) had increased by 0.6% (Department of Statistic Malaysia, 2020). However, the growth of urban population was much higher than total population in Malaysia. The population who live in urban areas has increased from 34.2% in 1980 to 71% in 2010 (Department of Statistics Malaysia, 2010) to 76.2% in 2020 (Department of Statistics Malaysia, 2020). Within Malaysia, Kuala Lumpur and Putrajaya have been reported to be fully urbanized (100%) (Department of Statistics Malaysia, 2010).

The rapid urban population growth is primarily caused by three main factors, which are natural cause, migration and reclassification (United Nations, 2019). The natural increase in urban population is related to the birth rate and death rate in urban areas while both the rates are dependent on the fertility level, life expectancy and the age distribution (United Nations, 2019). In country that urbanized hastily such as Africa, the natural causes of urban population growth include high fertility rate (5 children per woman) and longer life expectancy (Cobbinah et al., 2015a). Besides that, migration from rural to urban areas also increase the urban population when the number of migrants moving in exceed the number of migrants moving out (United Nations, 2019). The most prominent evidence of effect of rural-to-urban migration on urban population growth can be seen in China, where the largest human migration happened in history (Gong et al., 2012). Another factor contribute to urban growth is the reclassification of area, which involve expanding size of urban area and incorporating neighbouring formerly rural areas into urban areas (United Nations, 2019).

In Malaysia, the substantial urban population growth was primarily due to migration and the natural causes (Chamhuri et al., 2016). In 2018, the percentage of internal migration was as high as 89.3% (Department of Statistics Malaysia, 2019). Among the internal migrants, 28.5% of them were inter-state migrants while 60.8% of them were intra-state migrants (Department of Statistics Malaysia, 2019). Most intra-state migrants (66.9%) were moving from urban to urban, followed by rural to urban (19.8%) (Department of Statistics Malaysia, 2019). The natural increase in urban population in Malaysia might be due to the natural increase in total population as in fourth quarter in 2019, the number of live births (124,240 people) had exceeded the number of deaths (43,264 people) and the number of natural increase was as high as 80,600 people (Department of Statistic Malaysia, 2020).

2.2.2 Poverty

The concept of poverty is complex and has morphed for decades due to its multidimensional nature. The definition of poverty has evolved from primary concept that merely used income as indicators to more advance concept that used human development and environmental sustainability as indicators (Cobbinah et al., 2013). Consequently, the most common definition to describe poverty is related to monetary income or expenditure. The indicator that most frequently used in the world is the World Bank's International poverty line which set at US$1.90 (RM 4.35 as converted by using exchange rate in April 2020) in a day. Despite of monetary definition, the term of poverty can also be broadly described as deprivations that avert people from satisfying basic living needs (Asian Development Bank, 2014) such as lack of resource to obtain certain type of diet and customary living conditions or amenities, social exclusion, absence of participation in political process and socio-economic activities and structural problems that cause homelessness, joblessness, and vexation (Cobbinah et al., 2015b).

The concept of poverty is dynamic in nature in which in realistic, the condition of poverty described in a developing country is different from poverty as described in a developed country. Poverty in developing countries is related to severe deprivation that compromises basic living standard, while in developed countries, poverty issues are more focused on benefits and welfare provisions of residents (Cobbinah et al., 2015b). The concept of poverty in developing countries can be collectively described in three perspectives, which are social, environmental and economic aspects. From the social perspective, poverty can be described by using welfare indicators such as mortality rates, life expectancy and school enrolments. For example, poor people are characterized as suffering from high infant mortality rate and low life expectancy (Cobbinah et al., 2015b).

From environmental perspective, poverty can be defined through the degree of environmental degradation and the dependence on natural resources and environment for livelihood. For example, poor people are having attributes that excessively depend on natural resources as source of revenue such as by using forest clearing and traditional agriculture methods (Cobbinah et al., 2015b). Economically, poverty in developing countries is characterized as high probability of unemployment, especially in urban areas. Although there are more job opportunities in urban, not all benefits due to unsatisfactory working condition and spatial location of informal settlements in urban (Baker, 2008).

The concept of poverty can also be divided as absolute poverty and relative poverty. Absolute poverty is denoted as a situation with deprivation that merely focus on economic quantitative indicators such as income, monetary resources, level of goods that require to ensure minimum subsistence or to fulfil basic human needs including food, clothing, health, education and shelter (Cobbinah et al., 2013; Khoo et al., 2018). Unlike absolute poverty, relative poverty is more likely to describe as deprivation that related to inequalities in society, cultural and social needs (Khoo et al., 2018).

Over the past centuries, the world has done extraordinary works in reducing extreme poverty. By defining poverty based on the international poverty line (IPL) as income less than US$1.90 a day in 2011 purchasing power parity (PPP), the poverty rate was decreasing sharply from 36% in 1990 to 10% in 2015 (The World Bank, 2018) . It was estimated that 736 million people living in poverty in 2015, which had decreased tremendously compared to 2 billion people living in poverty in 1990 (The World Bank, 2018). However, although the trend is decreasing the number of poor worldwide is still alarming. This has urged the actions of governor across the world to end poverty in all forms which also has been proposed as the first SDGs by the United Nations (2015). This SDG is set to eliminate poverty worldwide and to reduce at least half of all population living in poverty by 2030 (measured as less than US$1.25 a day) by ensuring equal rights to resources or services and by decreasing their vulnerability to shock and unsound policy (United Nations, 2015).

In Malaysia, the concept of poverty is harder to describe because Malaysia is a country of diverse ethnicity with many different social, cultural and also economic structures that need to be considered (Khoo et al., 2018). Traditionally, the concept of poverty in Malaysia is more referred as a person's monetary ability to fulfil basic needs. Poverty in Malaysia was previously measured by using poverty line income (PLI) only (Chamhuri et al., 2016). PLI is a minimum income level to measure the absolute poverty which has been defined nationally to differentiate poor and non-poor. Household who earn less than the definite income level is classified as poor or vice versa. At national level, the mean PLI was set at RM2208 monthly (Department of Statistics Malaysia, 2020). By using this measurement, the percentage of absolute poverty in 2019 decreased from 7.6% in 2016 to 5.6% in 2019, while the hardcore poverty also reduced by 0.2%, from 0.6% in 2016 to 0.4% in 2019 (Department of Statistics Malaysia, 2020). Nevertheless, the merely use of PLI to measure national poverty in earlier years to measure

national poverty level was criticized because PLI is an absolute approach to measure poverty which totally excluded the consideration of deprivations related to poverty such as living standards, education and health.

Hence, despite absolute poverty, relative poverty is also measured in Malaysia by comparing household income with certain living standards set by the society. Relative poverty in Malaysia is defined as half the median income of all households in a given year which greatly depend on the current household income distribution (Department of Statistics Malaysia, 2020). A household is classified as relative poor when the total income in that particular household is below the threshold level of relative poverty. In 2019, Malaysia recorded a higher value of relative poverty (16.9%) compared to 15.9% in 2016 (Department of Statistics Malaysia, 2020).

Following that, changes have been made whereby Malaysia's poverty eradication strategies become more emphasised on equitable economic distribution at all levels with the vision to become sustainable developing country and determination to narrow income inequality gaps. By applying the concept of fair income distribution, abundant methods to measure poverty have been introduced and implemented. Gini coefficient is one of the most popular methods of determining income inequality in Malaysia. Gini coefficient is a kind of measurement using the Lorenz curve to assess the wealth inequalities, in which a lower value of Gini coefficient implies a fairer distribution of income (Department of Statistics Malaysia, 2020). In 2019, the Gini coefficient measured on the basis of gross income was 0.407, which increased by 0.008 percentage points from 0.399 in 2016 (Department of Statistics Malaysia, 2020), indicating an increase in the income inequality gap.

Furthermore, the Multidimensional Poverty Index (MPI) is also introduced to measure poverty in a broader perspective. This index measures poverty by reflecting the degree of deprivation in non-monetary aspects such as education, health and basic amenities and a lower value of MPI indicates a lower degree of deprivation. According to the Report of Household Income and Basic Amenities (2020), the national MPI in 2019 was 0.0110 (1.1%) points. The value showed a great improvement (decrease by 0.0042 index points, 0.42%) compared to 0.0152 points (1.5%) in 2016.

In these few years, the focus of poverty eradication strategies and policies has been shifted to low income group who is denoted as B40 income group that represent the bottom 40% of household income distribution of Malaysian population (EPU, 2015). The middle-income group is denoted as M40 income group who represents the households within 41% to 80% of household income distribution and upper-income group is described as T20 income group who are the top 20% of the distribution. A household with an income less than RM4,850 is categorised as B40 income group, a household with a range of income between RM4,851 and RM10,970 is classified as M40 income group and a household with an income more than RM10,971 is referred as T20 income group (Department of Statistics Malaysia, 2020). The mean and median income levels for each group in 2016 and 2019 are shown in Table 2.1.

Table 2.1: Monthly household income by income groups and years

**Income classification**	**Income share**		**Mean household income (RM)**		**Median household income (RM)**	
	**2016**	**2019**	**2016**	**2019**	**2016**	**2019**
B40	16.4%	16.0%	2,848	3,152	3,000	3,166
M40	37.4%	37.2%	6,502	7,348	6,275	7,093
T20	46.2%	46.8%	16,088	18,506	13,148	15,021

Source: Department of Statistics Malaysia (2020)

In the recent Report of Household Income and Basic Amenities 2019 (Department of Statistics Malaysia, 2020), the household groups of B40, M40 and T20 are further divided into ten categories according to ten percentiles with the purpose to ease the monitoring and implementation of strategies to targeted groups and to narrow the income disparities between households. The B40 group can be further categorized into B1, B2, B3 and B4; M40 group can be further classified as M1, M2, M3 and M4; while T20 group can be further classified into T1 and T2. The income threshold and the median and mean household income levels for each subdivision of each income group are shown in Table 2.2.

Table 2.2: Median, mean household income and income threshold by household decile groups in 2019

Decile group		Income share (%)	Median Household Income (RM)	Mean household income (RM)	Income threshold (RM)
T20	T1	30.7	19,781	24,293	> 15,039
	T2	16.1	12,586	12,720	10,960-15,039
M40	M1	12.3	9,695	9,730	8,700-10,959
	M2	9.9	7,828	7,841	7,110-8,699
	M3	8.2	6,471	6,477	5,880-7,099
	M4	6.8	5,336	5,346	4,850-5,879
B40	B1	5.6	4,387	4,395	3,970-4,849
	B2	4.5	3,556	3,561	3,170-3,969
	B3	3.5	2,786	2,803	2,500-3,169
	B4	2.4	1,929	1,849	< 2,500

Source: Department of Statistics Malaysia (2020)

Nonetheless, the classification of income group is rather confusing in Malaysia as different income level has been used in different setting. For example, one of the requirement for low income group to apply for People's Housing program is that the household income of applicant to be not more than RM 3,000 but in Budget 2020, the maximum income for B40 income group is uplifting to RM4,360 for the project of Fund for Affordable Home that was launched in 2019 to assist B40 income group to purchase their first home (Ministry of Finance Malaysia, 2019). A clearer and standardized definition for the income level of each income group in different settings should be implemented throughout Malaysia for easy classification.

In summary, Malaysia reported a relative lower value of absolute poverty (5.6%), higher value of relative poverty (16.9%), higher Gini coefficient (0.407 percentage points) and improved MPI value (0.0110 (1.1%) points). These measurements indicate that although the poverty incidence reported in Malaysia is improving, the fact that the increasing income inequalities gaps between households and over millions of Malaysians are still struggling to survive in poverty shall not be ignored.

2.2.3 Poverty in urban

Historically, the issues of poverty in urban areas were less discussed. However, with the rapid development and hasty urbanization that keep increasing population living in urban areas, the emergence of urban poor is unavoidable. By using the most common definition, urban poverty can be defined as a situation when the urban household has low income or income falls below the poverty line which exposed them to deprivation that prevent them from meeting basic living standard (Khoo et al., 2018). While the urban poor population is struggling to survive in high living cost cities, they are being left out and excluded from the initiatives to reduce poverty as

most of the progress in reducing poverty in developing countries were mainly focused on rural areas (Tacoli et al., 2015). Ravallion et al. (2007) had revealed that the urbanisation of poverty had occurred as early as in 2007 by showing that in the total decrease in poverty rate (8.7%), the poverty rate decline in urban areas (2.3%) was much slower than in rural areas (4.8%) by using US$2 in a day as poverty line, indicating that the poverty was urbanized by considering that the urban population will keep increasing in the future.

It was estimated that one third of urban residents worldwide were poor, which represented approximately one quarter of the world total poverty rate (Ravallion et al., 2007). As stated in the Global Monitoring Report, the share of urban poor in Asia was increasing since 1990 from 18.3% to 22.9% in 2008 (World Bank & International Monetary Fund (IMF), 2013). In Asia, it was estimated that there was 189 million poor population lived in urban areas (13.7%) and accounted for 69% of developing countries urban poor population (Asian Development Bank, 2014). In Malaysia, the poverty rate in urban areas was decreasing from 1% in 2012 (Department of Statistics Malaysia, 2014) to 0.2% in 2016 (Department of Statistics Malaysia, 2017). In 2019, the absolute poverty in urban areas was as low as 3.8% compared to rural (12.4%) and national level (5.6%) (Department of Statistics Malaysia, 2020). The mean and median household income in urban areas (RM6,635; RM6,561) also reported the highest value than national income level (RM7,901; RM5,873) and in rural areas (RM5,004; RM3,828) (Department of Statistics Malaysia, 2020).

In fully urbanized area such as Kuala Lumpur, the absolute poverty (0.2%) and relative poverty (10.6%) are also relatively low (Department of Statistics Malaysia, 2020). However, the existence of urban poverty is becoming visible (Chamhuri et al., 2016; Khoo et al., 2018; Shahar et al., 2019) in Kuala Lumpur. In fact, the government initiatives to reduce incidence of poverty among low-income group through the implementation of People’s Housing Project (*Program Perumahan Rakyat*, PPR) within whole Malaysia (Ministry of Housing and Local Government, 2018) has indirectly disclosed the solid proof of emerging of urban poverty as this program is focusing and implementing in Kuala Lumpur.

Indeed, urban poor population are suffering from inequalities as much as those poor living in rural areas because they are invisible and marginalised as a result of the norm and expectation that people who capable to resident in urban area are richer and having better living quality. Hence, the rights and welfare of this group of population shall be protected and emphasised by governors across the world to reduce the inequalities occurred within and between countries as elucidated in SDGs. Reduce inequalities as explained in the tenth SDGs is meant to ensure equal opportunities of all populations to enjoy welfares in term of social, political and also economic perspective (United Nations, 2015).

2.2.4 Deprivations associated with urban poor

The income-based definition seems to unlikely to represent urban poverty accurately (Tacoli et al., 2015) because urban poverty is not merely a concept that can be defined merely through income level but indeed it has many dimensions that always in line with the deprivation of basic privileges such as education and foods (Khoo et al., 2018). The deprivations associated with urban poverty include lack of government policy, inadequate law protection, inability to reach out to financial services, limited or absence of safety net, poor housing condition, high living costs (Tacoli et al., 2015) and lack of access to proper education and health (Asian Development Bank, 2014).

Firstly, urban poor population is characterized as unable to enjoy privileges or benefits which help them to escape poverty due to the incompetence of political systems or policy structures that are supposed to protect them (Tacoli et al., 2015). This population is also not protected by rights through entitlement of law in the way that they are not actually and fully

recognizing their civil and political rights (Tacoli et al., 2015). Besides that, urban poor population is attributed to be incapable to reach out for financial services such as services that help to secure asset base, amenities that help to prevent from sudden erosion of assets due to injury, illness and shocks and also safety net that include insurance and organizations that provide strategies to survive (Tacoli et al., 2015).

The most common deprivation experienced by urban poor population is inadequate and uneven income due to reasons such as job-related risks that cause frequent injury or illness, payment for medical treatment, little opportunity for better income (Tacoli et al., 2015), informal employment and working poor (Asian Development Bank, 2014). They have to bear the growing living cost (Khoo et al., 2018) and high prices for public necessities such as water, sanitation, school, health care and transports (Tacoli et al., 2015). When the income is merely enough for them to survive, they unable to afford better quality residence in which many of them will compromise and choose to stay in informal settlement, cheap tenements or urban slum (Asian Development Bank, 2014). Their residence conditions always characterized as overcrowded, poor ventilation, not safe, inappropriate sanitation (Fotso et al., 2012), lack of access to public transport and basic services, which expose them to additional risk of injury or accident, higher living cost, and higher health burden (Tacoli et al., 2015).

Another vital deprivation associated with urban poverty is related to health including the social services and foods (Cobbinah et al., 2015b). Urban poor population who often experience inadequate income tend to compromise the diet in term of quality and diversity (The World Bank, 2018), which then lower their immunity and they will fall ill sooner (Cobbinah et al., 2015b). Besides, poor housing conditions might also affect their ability to access to public health infrastructure or safe and nutritious foods which increase the risk of infectious diseases and malnutrition (UNICEF, 2015).

Amongst the urban poor population, young children seem to be more vulnerable than adults (Fotso et al., 2012) as both urbanisation and poverty are associated with intrahousehold inequalities on food consumption (The World Bank, 2018). According to the World Bank (2018), in a household, women and children are allocated with smaller share of resources compared to men and children tend to consume less than their mothers. A study conducted by Fotso et al. (2012) revealed that food poverty (household's access to food) was significantly associated with stunting while assets poverty (household ownership of durable assets) and subjective poverty (households' perception toward their own wealth status) were strongly associated with undernutrition in children. In a study on urban child poverty and deprivation in low-cost flats in Kuala Lumpur, the prevalence of underweight (15%), stunting (22%) and wasting (20%) were high in young children as they were living and growing up in underprivileged environment that was not safe and lack of access to enough and healthy foods (UNICEF, 2018). The findings of the UNICEF report (UNICEF, 2018) had raised concerns about the health of urban poor population and drew more attention from stakeholders to improve the welfare of this group of people.

In summary, the urban poor population is somehow being ignored and less emphasised in the effort of stakeholders to end poverty internationally and nationally. This group of people is deprived from basic living standards and suffered as much as other poor population. Despite of adults in urban poor families are bearing the consequences of urbanisation and poverty, those children who grow up in such families are more vulnerable than their parents and at higher chances to be subjected to undernutrition. There is an immediate need to draw attention of stakeholders on this ignored group of population who deserves to share and enjoy the basic services and welfares as others.

2.3 Positive Deviance

2.3.1 Definition

The term ‘deviance’ is generally referred as anything which can be either a person or a behaviour that is not following the norm or is distinctive from traditional or current route. The term can be either denoted positively or negatively (Herington & Fliert, 2017). Negative deviance is any behaviours or practices that are violated from norm but are considered as harmful and destructive (Herington & Fliert, 2017). In contrast, positive deviance is described as any uncommon norm, behaviours or practices that happened in a more honourable way that it is beneficiary and able to successfully unravel community problem (The CORE group, 2002). Positive deviants have been identified as individuals who excel in finding a way to transcend obstacles and actively pursue those actions or behaviours under the same socio-economic distress as their peers (Schooley & Morales, 2007).

Positive deviance can be described in two typical ways, which are either from theoretical perspective or as practical strategy. From theoretical perspective, positive deviance is defined as a concept that explained the occurrence, impact and role of positive deviance toward human knowledge on sociology (Herington & Fliert, 2017). In practical setting, positive deviance can be designated as an adaptive tool to develop and implement effective strategies that is observed from outperformed peers in the same resource-challenged environment to address the social problem (Albanna & Heeks, 2019). In the beginning, positive deviance only served as theory and concept in disciplines such as sociology and psychology to study the nature of world but in later years it has been applied in multiple disciplines especially in the field of child nutrition (Herington & Fliert, 2017). Positive deviance in child nutrition is a community-based participatory nutrition education approach specifically described as the adaptive child care practices, positive hygiene practices, and feeding practices that enable children to grow normally in harsh environment with restricted resources (Zeitlin et al., 1990).

The theory of positive deviance was believed to be originated from the sociological studies of deviance in the early 1900s that mostly aimed to understand the phenomena of people acting or behaving outside the normal margin or typical norm (Herington & Fliert, 2017). The incorporation of positive deviance in literatures were polemic in the early stage as robust of sociology and psychology studies were mainly focused on negative deviance and had caused the arising of negative perception on this concept (Herington & Fliert, 2017). It was until 1960s for the positive deviance to be accepted widely after the illustration of the possibilities of violation of norm in positive way by using bell curve (Herington & Fliert, 2017). Since then, the concept of positive deviance is more broadly applied by researchers from different disciplines such as child malnutrition, infant and young child feeding practices, infectious disease (Albanna & Heeks, 2019).

According to Sternin et al. (1998), PD approach is an approach that is rooted in the principle that problem existed in a community can be solved by solutions that are already existed within community. It is also closely related to other approaches or theories such as asset-based approach and health belief model (HBM). As denoted in abundant studies (Albanna & Heeks, 2019; Pascale et al., 2010; Sosanya et al., 2018; Sternin, 2002), PD approach shares similarities with asset-based approach. Asset-based approach involves identification and mobilisation of local asset (GCPH, 2012) while PD approach utilizes a community’s inherent wisdom including knowledge to generate better solutions for a community’s problem (Albanna & Heeks, 2019).

Positive Deviance approach is also referred as a community-based participatory nutrition education approach rooted in the Health Belief Model (Roche et al., 2017). The fundamental constructs in HBM include perceived susceptibility, perceived severity, perceived benefits, perceived barriers, cues to action and self-efficacy (Glanz et al., 2008) while for PD

approach also focuses on building social support and self-efficacy to invoke positive behaviour change (Dickey et al., 2002). However, the definite relationship between PD approach to these approaches is rather ambiguous, indicating more research regarding this relationship are required.

According to Sternin et al. (1998), PD approach in a nutrition program for malnourished children might function as discovering tool to find out the locally available nutritious foods that affordable for mothers to feed their children and as a communication way to persuade mothers of undernourished children that solutions do exist within community. Based on the guidelines provided by The Child Survival Collaborations and Resources Group (The CORE group), the ultimate goals of a PD program are to promote nutritional recovery among malnourished children, to ensure sustainable rehabilitation of these children and to prevent future malnutrition within community (The CORE group, 2002). In a PD nutrition program, participants who are mostly mothers of malnourished children need to change their behaviours by practicing new practices that identified from the Positive Deviance Inquiry (PDI) with the help of volunteers who are being selected from the same community (Sternin et al., 1998).

Although previous programs were mostly conducted in poor rural setting, the programs run as well as in urban setting. According to the CORE group, urban PD projects also existed in countries such as Haiti, Ethiopia, Guinea, Madagascar, Indonesia and India (The CORE group, 2002), but none in Malaysia. With the high prevalence of childhood malnutrition in Malaysia, there is a need to employ innovative and sustainable strategies that are different from traditional strategies.

2.3.2 History

In 1976, it was the first time for the concept of PD to be proposed and utilized as a practical strategy in designing a food supplementation program based on feeding and care practices identified from families who had well-nourished children in an impending study by Wishik and Van Der Vynckt in Central America (Wishik & Van Der Vynckt, 1976). The study was the first study to referred low-income families with well-nourished children as positive deviants. However, the study only proposed the methodology to implement program by using PD approach without publishing the results of the program (Albanna & Heeks, 2019). Hence, the use of PD in practical sense was not widely recognized until the first launch of program by Save the Child in 1990s that adopted PD approach (Zeitlin et al., 1990) with Hearth session (Wollinka et al., 1997) to reduce child malnutrition in a community setting in Vietnam (Herington & Fliert, 2017).

The PDI conducted by Save the Child in Vietnam was very successful in identifying numerous PD practices. For example, feeding children with a combination of small shrimp from rice paddies and greens vegetables sweet potato tops was found to be an important PD practices but most of the mothers in the community did not believe that their children should be fed with this combination of food (Sternin, 2002). With the huge accomplishment of the Vietnam program in decreasing incidence of moderate and severe malnutrition among children under age of three by an estimated 55% to 85% (Sternin, 2002), PD approach is gaining popularity and is scaled up into bigger and complete model at international context.

Later, with the increasing establishments of manuals and guidelines on the use of PD approach in unravelling community problems such as childhood malnutrition (Sternin, 2002; Sternin et al., 1998; The CORE group, 2002; Zeitlin et al., 1990), the concept was developed and had been integrated with the Hearth model in the field of nutrition, resulting in the implementation of PD/Hearth program (The CORE group, 2002). The incorporation of Hearth model in nutrition program was introduced by Berrggren and Burkhalter and the key concept of this model is the active participation of parents in rehabilitating their malnourished children at home (Wollinka et al., 1997). The implementation of PD/Hearth model in nutrition program

was estimated to be started around 1999 until today with the promotion from some organizations such as USAID, CORE group, the Peace Corps, Save the Children, World Vision and UNICEF (Bullen, 2011; The CORE group et al., 2003).

In summary, the PD/Hearth program is a community-based nutrition rehabilitation model that claims to be sustainably rehabilitate malnourished children, decrease malnutrition prevalence and prevent malnutrition in future (The CORE group, 2002). This approach is widely used in the endeavours to eradicate malnutrition in children across more than 45 countries and with success of launching over 100 projects worldwide (Springer et al., 2016). For example, The Maternal and Young Child Health Initiative (MYCHI) in India, PD/Hearth approach to Child Nutrition - Child Survival (CS19) in Afghanistan, The 'Ame Bi Paribu' program in India, and the program of Improving Newborn, Child, and Maternal Care in Vietnam (PDI, 2017). Nonetheless, Malaysia is one of the countries where no PD program has been carried out although high prevalence of undernutrition was reported.

2.3.3 Positive deviance methodology

Different methodology to implement PD program has been proposed and presented throughout these years (Pascale et al., 2010; Sternin et al., 1998; The CORE group, 2002). According to the guideline provided by Sternin et al. (1998), the process can be described in five steps, which are identification of local resources, situational analysis, positive deviance inquiry (PDI), design of program according to PDI findings and implementation of program. In contrast, Pascale et al. (2010) had proposed four D's as key steps to implement PD program, including define problem and desired outcomes, determine common practices and occurrence of positive deviants, discover uncommon but successful behaviours and strategies through inquiry and observation, design suitable strategy based on the findings. In contrast, the guidelines of the CORE group had proposed nine vital steps in designing PD program (The CORE group, 2002) as shown in Figure 2.4. Although variations in PD methodology have been used in different studies, the concept and fundamental key activities to be conducted are still similar.

There are a series of activities that are mandatory to be conducted in a PD nutrition program to introduce new behaviours, practices and skills, empower parents to be responsible in rehabilitating their children, improve parents' confidence level and reinforce the possibilities of sustainable behavioural changes (The CORE group, 2002). The main components of the program include a Positive Deviance Inquiry, including both observations and focus group discussion to identify successful behaviours among positive deviant families, monthly growth monitoring and monthly Nutrition Education and Rehabilitation Sessions (NERS) with peer-leading cooking and education sessions (Sternin et al., 1998).

When going through the program, the malnourished children are also being fed with additional high caloric foods by their mothers and the meals being prepared are based on the menu constructed with the positive deviant foods identified from the positive deviance family (The CORE group, 2002). The education and rehabilitation sessions in a traditional PD program is conducted for 10 to 12 days within 1 to 2 weeks for quick rehabilitation and the follow-up is carried out within two weeks after the session to ensure enough days for changing of learned new practices into habit (The CORE group, 2002).

Step 1: Feasibility

This step is to decide the practicability of using the approach in the target community by considering:

- general situations (location, local foods availability and prevalence of community problems)
- community involvement (presence of community leadership and availability of volunteers)
- agency commitment (availability of trainers and staff)

↓

Step 2: Community mobilization

This step involves the selection of community leaders and health personnel and training of volunteers or participants.

↓

Step 3: Preparation for PDI

This step is to prepare personnel to conduct PDI by determining target group and training that include teaching to conduct nutritional assessment, observation during visit and situational analysis.

↓

Step 4: PDI

This step involves systematic arrangement of home visit, resemble the findings and share the findings to community.

↓

Step 5: Design context of NERS

This step involves the planning of schedule and menu, design the content and protocol according to findings from PDI and planning of activities in advance.

↓

Step 6: Conduct NERS

This step involves the collection of materials, setting up of activities, leading of the session and supervision of the progress.

↓

Step 7: Support new behaviours

This step is to conduct follow-up home visits for those participants and followed by considering the necessary to repeat education and rehabilitation sessions.

↓

Step 8: Repeat NERS as needed

This step is to ensure malnourished children in community are not left behind by determining the needs of other children to join NERS and repeating the process periodically.

↓

Step 9: Expand or promotion of program

This step involves development of program in smaller scale, working out the possible to scale-up the program and support new researcher to return to home for replication.

Figure 2.4: Steps in a PD/Hearth program

Source: The CORE group (2002)

2.3.4 Positive Deviance Inquiry

The successful practices that lead PD families to be more likely to have well-nourished children are required to be discovered by performing Positive Deviance Inquiry (PDI) prior to designing and implementing the PD program. The methods to carry out PDI differ across study but the two methods that are highly recommended or commonly use are observations and guided interviews (Sternin et al., 1998). It was also recommended to form a PDI team to conduct PDI, consisting of trained workers or volunteers from the targeted community instead of outsiders of community (Sternin et al., 1998). However, the use of PDI team to conduct PDI might result in labour extensive and possible failure of the PDI team to conduct household observation which later lead to inconclusive findings (Sternin et al., 1998). Hence, the most suitable people to conduct PDI in targeted community such as researcher should be considered and clearly determined in advance to minimize the possible failure to identify any PD practices or food.

The traditional PDI process involves identification of PD and NPD family, discover of successful practices from PD families and harmful practices or good practices that are lack practised in NPD family, selection of accessible successful practices and incorporation of these practices in designing program (Sternin et al., 1998). There are three criteria in selecting the key good practices identified and recorded from PD families, which are the practice must be accessible to everyone in the community, the practice is unique or different from norm and the practice is replicable. Yet, Lapping et al. (2002) had proposed an interesting approach to carry out PDI which was booster PDI. This new idea proposed an on-going PDI throughout the program to identify new positive deviants who adopted new behaviours during the process and to find out the reason for adoption and change in behaviours (Lapping et al., 2002). However, Lapping et al. (2002) also reminded of the challenges in using this booster PDI such as extensive labour, observations and household visits.

The findings of PDI are mainly focused on three areas which are feeding practices, caring practices and health-seeking practices (Sternin et al., 1998). An important principle to bear in mind is that not all successful practices identified from PD families are applicable to be used as key practices to promote to other community members, as the identified practices may sometimes be true but useless. Several challenges may be encountered such as unable to identify PD families, unable to identify PD behaviour, inconclusive PD findings, and fail to identify PD foods. Meanwhile, PDI is a very vital step to be taken in applying PD approach as intervention, however, most of the previous intervention studies did not report the main findings of PD practices and foods (Calvince et al., 2015; Inamahoro et al., 2017; Kang et al., 2016; Srivastava et al., 2019). There are only a few reports, guidelines or studies reported the key findings of PD feeding practices, caring practices and health-seeking behaviours (Roche et al., 2017; Sternin et al., 1998; The CORE group, 2002; The World Bank group, 2018).

An intervention study conducted by Roche et al. (2017) in Ecuador aiming to improve the anthropometric measurements and dietary intake of young children aged <24 months by using PD approach had identified the PD feeding practices, which included children being fed a variety of local foods daily, participation of children in preparing new recipes with local foods, exclusive breastfeeding and fed breast milk only until 6 months and timely introduction and consistency (start to feed purees, not just broth at 6 months). The study also showed that responsive feeding in which mother sits with child when eating and feed with love and affection was considered as PD caring practices (Roche et al., 2017). The similar study also reported that the PD hygiene practices discovered in the rural communities in Ecuador included wash hands and face with warm water before cooking and eating and keep animals out of the kitchen (Roche et al., 2017).

In contrast, another report by People in Need (PIN) in 2019 has demonstrated quite different PD practices and foods in Zambia and Ethiopia. The report illustrated the steps and

effectiveness of programs using PD approach to prevent child malnutrition, rehabilitate underweight children and encourage sustainable nutrition practices among rural communities in Zambia and Ethiopia (People in Need, 2019). The PDI was conducted through household observations to explore the differences in practices between PD and negative deviant (ND) households (referred as household with malnourished children). The outcomes of PDI included PD feeding practices such as active supervision of feeding, encouragement to eat, feed freshly cooked food, feed a variety of different food and good strategy to manage low appetite or when the child is sick; while for PD caring practices, active involvement of father in caring for children was reported (People in Need, 2019). Meanwhile, the PD health practices reported in this report included given correct treatment of diarrhea for children such as provision of Oral Rehydration Salts (with Zinc), feeding thin liquid porridge and continuing to breastfeed during the illness (People in Need, 2019).

Despite PD practices, PD foods, the specific nutritious foods being fed to children in PD family can also be identified through PDI. The PD foods are foods that are normally consumed and will be promoted to targeting communities (Sternin et al., 1998). For example, PD foods identified in mountain area of Nepal were snails, frog, crabs, fish, peanuts, wild berries and mulberries (Sternin et al., 1998). The types of PD foods are varied in different settings. For instance, the PD foods identified in rural communities of Ecuador were quinoa, large fava beans, lupine seeds, mixed beans, nettle, blackberry, cheese, guinea pig, tuna and liver (Roche et al., 2017). The PD foods discovered in rural communities of Zambia and Ethiopia were nuts, small fish, dark green leafy vegetables, tomatoes, cow peas, local "traditional" peas, hibiscus and ground maize (People in Need, 2019).

It is observed that numerous reports or studies did not show the main findings obtained in the PDI phase. Given that the findings or knowledge regarding PD practices and foods in a particular community are essential in promoting such good practices in the community, documenting and reporting those findings is extremely crucial.

2.3.6 Effectiveness of positive deviance program

Ample of positive deviance studies that focused on rehabilitating malnourished children in resource-poor setting have shown positive results in either gaining weight in children or decreasing the prevalence of undernutrition (Sosanya et al., 2018). For instance, a quasi-experimental study conducted in Migori, Kenya that involved children aged 6 to 59 months from 53 families as intervention group and 54 families as control group had shown significant decreased in underweight prevalence and weight gain in intervention children (Calvince et al., 2015). Findings showed that those children who engaged in the PD program showed an increased in mean WAZ by 0.36 SD. The prevalence of mild and moderate underweight among children in the intervention group was as high as 43.4% and 18.9%, respectively. However, after participating in the PD program, the prevalence had decreased to 34% for mild underweight and 3.8% for moderate underweight at 2-week follow-up. Besides, caregivers who engaged in the PD program also showed significant improvement in feeding practices, in which over 90% of the caregivers increased feeding frequency and food variety to their children and over 60% practised exclusive breastfeeding (Calvince et al., 2015).

Another quasi-experimental non-randomized study conducted by Roche et al. (2017) in Ecuador also showed great success of using PD approach to improve diet and reduce undernutrition in children aged below 24 months. The study involved 80 mother-children dyads from six communities as intervention group (those who participated in the PD program) and 184 mother-children dyads from 9 communities as control group. Children in the intervention group consumed higher mean energy intake with higher percentage of recommended intakes for energy, protein, vitamin A, iron, and zinc at 6-month follow up. These children also gained

weight significantly by 0.16 WAZ with prominent decrease in the prevalence of underweight from 30.4% at baseline to 23.7% at 6-month follow-up. Additionally, the prevalence of severe underweight in children was also significantly decreased by 8.7% from 10% at baseline to 1.3% at 6-month follow-up. Furthermore, mothers in the intervention group were 1.3 to 5.7 times more likely to feed their children with the promoted foods such as quinoa, mixed beans, blackberries, canned tuna, and others (Roche et al., 2017).

Besides that, a two-stage randomised cluster study done by Inamahoro et al. (2017) in Burundi comparing the effectiveness of two nutrition interventions, namely the PD program and the community health worker nutrition promotion (CHWNP) in improving nutritional status of moderately malnourished under-five children indicated that children who engaged in either program showed improvement in their nutritional status, including underweight, wasting, stunting, and mid-upper arm circumference. Findings further showed that children who enrolled in CHWNP were more likely to recover with significant improvement in nutritional status than children in PD program (Inamahoro et al., 2017). In contrast, a cluster randomized trial carried out in rural Ethiopia presented different results (Kang et al., 2016). Kang et al. (2016) showed that children who participated in a community-based participatory nutrition promotion (CPNP) program adopting PD approach which added to existing government programs had a significant increase in HAZ and WHZ with decrease in prevalence of stunting by 8.1% and underweight by 6.3% compared to children who only participated in the existing government programs only.

A systematic review carried out by Sosanya et al. (2018) presented abundant evidence of positive effects of PD program in rehabilitating malnourished children and preventing future malnutrition across multiple countries such as Zambia, Burundi, Uganda, South Africa, the Philippines, Nepal, Indonesia, India, Pakistan and Ethiopia. However, another systematic review focusing on the PD approach in reducing malnutrition among children demonstrated mixed results in term of the effectiveness of the PD program (Bullen, 2011). Bullen (2011) reported that all the nine studies that used a pre- and post-test design without a control and two randomized controlled trials (RCTs) showed positive results of the PD program in reducing malnutrition, whereas for non-randomized trial, two studies showed positive results but one showed no effect.

Some positive deviance studies stopped at the phase of identifying positive deviant without further intervention, in which these studies have shown the usefulness of PD approach in identifying outperformed practices (Albanna & Heeks, 2019). A cross-sectional study carried out in Sarolangun Jambi, Indonesia to identify positive deviance feeding and caring practices among children under 5 from poor families (<RM560 per month) showed a significant relationship between positive deviance feeding practices (p=0.001) and caring practices (p=0.013) with nutritional status of children (Merita et al., 2017). Almost all mothers (94.7%) with good PD feeding practices and 94.4% of mothers with good caring practices had children with normal weight. Findings also showed many PD feeding and caring practices including mothers would tend to feed children with fish as animal protein with tempe and tofu, wash hand before feeding, actively feeding, early introduction and familiarization with local fruits.

A qualitative study done by Kanani and Popat (2012) among urban poor children aged 6 to 18 months in India via semi-structured interviews reported that the factors contributed significantly to PD feeding practices included older age of children, smaller family size, fed actively, lower parity, greater birth interval, breastfed at least 8 to 9 times in a day and complementary feeding started at 6 to 8 months. The study also showed that the PD approach fit the urban poor setting as similar to rural areas (Kanani & Popat, 2012).

Positive deviance approach was effective in unravelling other issues despite of the success of PD program in tackling childhood malnutrition problem (Albanna & Heeks, 2019).

Previous studies have shown the usefulness of PD approach in vast contexts such as improved the compliance of adolescents in anaemia control program in India (Sethi et al., 2017), improved hygiene and sanitation behaviours in children in rural areas of Kenya (Aday et al., 2016), enhanced breastfeeding practices among tribal pregnant women in India (Srivastava et al., 2019), prevent child marriage by abduction in Ethiopia (Lackovich-Van Gorp, 2017), infectious disease control in Colombia and Ecuador (Escobar et al., 2017; Nieto-Sanchez et al., 2015), improved student clinical performance in Pakistan (Zaidi et al., 2012), cancer risk reduction in Guatemala (Vossenaar et al., 2009) and female genital cutting and HIV/AIDS program in Egypt (Schooley & Morales, 2007).

The comparison of effectiveness of PD program with other interventions are rather difficult because vast different of outcomes were measured (Sosanya et al., 2018). The effectiveness of PD studies in term of reducing prevalence of undernutrition, high recovery rate and significant weight gain among children (Bullen, 2011; Calvince et al., 2015; Kang et al., 2016; Roche et al., 2017) was as promising as other studies with other interventions or approaches such as studies that merely use nutrition education as intervention (Chaudhary et al., 2018; Ragini et al., 2015) which showed significant improvement in height, weight, and mid-upper arm circumference; integrated nutrition program that showed high recovery rate (43.9%) (Aguayo et al., 2013); community-based nutrition education studies which indicated both significant weight gain and decreased in prevalence (Majamanda et al., 2014; Roy et al., 2007; Sánchez-Encalada et al., 2019; Sharma et al., 2020). However, in term of sustainability of intervention effect, the PD studies have shown more sustain intervention effects (Sosanya et al., 2018).

When comparing PD studies with interventional studies that only implemented supplementations such as by using ready-to-use therapeutic food (RUTF) (Isanaka et al., 2010; Nga et al., 2013; Shewade et al., 2013; Weber et al., 2017) or combination program of nutrition education and supplementation (Chaudhary et al., 2018; Ciliberto et al., 2005), the results of total weight gain and prevalence of malnutrition were lower in PD program. This might be due to the effectiveness of RUTF and Ready-to-Use Supplementary Food (RUSF) as management for moderate acute malnutrition (MAM) and severe acute malnutrition (SAM) is widely recognized due to the promising effect of high recovery rate within short period as indicated in previous studies (Chaudhary et al., 2018; Schoonees et al., 2019; Wagh & Deore, 2018). However, the sustainable effect of intervention is more prominent to be seen in PD program as compared to supplementation program (Schoonees et al., 2019; Sosanya et al., 2018). Therefore, intervention program that utilized PD approach seem to bring more sustainable effect which is important in ensuring the knowledge being taught and new positive habits being adapted during the intervention program can be continued and passed from generation to generation, breaking the chain of malnutrition in a family. The effectiveness of the PD program is shown in Table 2.3.

Table 2.3: Summary of the effectiveness of the nutrition programs using positive deviance approach

Authors	Country	Study design	Samples	PD behaviours	Intervention	Findings
(Roche et al., 2017).	Ecuador	Quasi-experimental non-randomized study<br><br>-measured at baseline (before intervention) and at 6-month follow up	-80 mother–child pairs in 6 intervention communities and 184 mother–child pairs in 9 comparison communities<br><br>-Young children aged <24 months<br><br>-Anthropometric measurements: WAZ, LAZ/HAZ<br><br>-Dietary intake: FFQ and 24-hour diet recall	Positive Deviance Inquiry<br><br>-Household visit and observation in 12 families of well-nourished children<br><br>-Gave a variety of local foods daily<br><br>-Participation of children in preparing new recipes with local foods<br><br>-Timely introduction and consistency (at 6 months start to feed purees, not just broth)<br><br>-Responsive feeding: Mother sit with child when eating and feed with love and affection<br><br>-Wash hands and face with warm water before cooking and eating<br><br>-Keep animals out of the kitchen<br><br>-Exclusive breastfeeding<br><br>-Fed breast milk only (until 6 months)	PD/Hearth approach<br><br>Hearth session<br>- 2-week peer cooking session<br>- Biweekly peer home visit<br>-Participating mother brought children to house of volunteers<br>-8 to 12 pairs of mothers prepared health meal by using repeated 3 to 4 menu and fed the children<br>-12 consecutive days	At follow-up, children of intervention group consumed higher mean energy intake (differences: 178±144 kcal for infants aged 6–12 months, p<0.05; 294±153 kcal for children aged 12–18 months, p<0.05; and 271±100 kcal for children aged >18 months, p<0.05) and higher percentage of recommended intakes for energy, protein, vitamin A, iron, and zinc than children in comparison group but the mean intakes for calcium, vitamin A, and iron were still below the recommendations intake.<br><br>At follow-up, there was a significant additional weight gain of 0.16WAZ in children of intervention group.<br><br>The prevalence of underweight decreased from 30.4% at baseline to 23.7% at follow-up and the prevalence of severe underweight decrease from 10% at baseline to 1.3% at follow-up among children of intervention group.<br><br>For children in comparison group, the prevalence of underweight increased by 2.2%, from 23.4% at baseline to 25.6 at follow-up and the prevalence of severe underweight increased from 5.1% at baseline to 6.2% at follow-up.<br><br>Intervention children more likely than comparison children to consume the foods promoted in the intervention, to consume more iron, zinc, vitamin A, protein, and energy and had greater improvement in WAZ but not HAZ.

(Inamahoro et al., 2017).	Burundi	Two stage randomized cluster with comparison of two methods (CHWNP and PD-H) and pre- and post-test	-840 children aged 6 – 59 months with moderately malnutrition who participated in either Positive Deviance - Hearth (PD-H) program or Community Health Worker Nutrition Promotion (CHWNP)<br><br>-Haemoglobin level was determined<br><br>-anthropometric indicators include MUAC and classification into underweight, stunting and wasting<br><br>- measured at baseline, at 12, 60 and 120 days.	Not stated	PD-H approach<br>-intensive training of caregivers for 12 consecutive days<br><br>- training once a week for up to 4 months.<br><br>CHWNP<br>- screening and referral to health centres	- Weight gain of 200g or more was considered as recovery.<br><br>-More than 60% of children recovered as day 12.<br><br>-The proportion of children discharged cured was statistically higher at 60 days compared to baseline.<br><br>-The recovery rate of children in CHWNP (81.9% in classification group based on MUAC, 24.3% in underweight group, 11.8% in stunting group and 69 in wasting group) were statistically higher from children who participated in PD-H program (75.1%, 14.5%, 9.8% and 46.6% respectively).
Kang et al., 2016	Ethiopia	Cluster randomized trial	-1790 children aged 6 to 12 months<br><br>-876 in the intervention and 914 in the control areas<br><br>-Cluster randomization whereby 12 clusters formed.<br><br>-Intervention group participated in community-based participatory nutrition promotion (CPNP) program that adapted a PD/H approach.<br><br>-Control group participated in existing Essential Nutrition Action (ENA) program and the	-Did not conduct any step to identify PD practices.<br><br>-By referring to World Vision PD/Hearth training manual.	- CPNP which consist of 12-day nutrition sessions and cooking session (from food preparation to food feeding) led by trained operators.<br><br>-existing CMAM involved delivery of nutrition messages and individual nutrition counselling by community workers or volunteers<br><br>-existing ENA involved referral to nearest health centres with supplementation of corn-soy blend for 3 rounds.	-Children in intervention group showed a greater increase in z scores for length-for-age (0.021 z score per month) and weight-for-length (0.042 z score per month) compared to control group.<br><br>-The prevalence of stunting (8.1%) and underweight (6.3%) among children in intervention group were lower than control group at 12 months follow up.

			Community-based Management of Acute Malnutrition (CMAM) program<br><br>-Anthropometric indicators include WAZ, LAZ/HAZ and WHZ<br><br>-Measurements at enrolment, 3-month, 6-month, 12 months follow up			
(Calvince et al., 2015)	Kenya	Pipeline quasi-experimental design	-Children aged 6 to 59 months<br><br>-53 PD intervention families and 54 comparison families<br><br>-Intervention group included malnourished children who participated in following program<br><br>-Comparison group included children in the PDI list<br><br>- Anthropometric measurements included WAZ<br><br>-Measured at entry (before entering session), exit (2-week follow-up after 12 days session), graduation stages (1 day after 12-week follow-up) and at current status (-month follow up after graduation)	-PDI conducted for 3 days but the outcomes were not stated.	PD/Hearth approach<br><br>-12 days Heart session<br><br>-children in intervention group fed with nutrient dense meal	-There was statistically increase in mean WAZ of the children in intervention group at entry and at current status. ($p<0.01$)<br><br>-On average, at current status, the feeding practices of caregivers improved.<br><br>-There were higher percentage of being fed on vitamin A rich vegetables (81.1%), increased feeding frequency (96.2%), increased food variety (84.6%) and increased feeding frequency after sickness (90.2%).<br><br>-There were significant differences of breastfeeding practices ($p<0.023$) and hygiene practices ($p=0.05$) at entry and at current status.<br><br>-Increase in feeding frequency was the factors contributed the most to the weight gain in children at current status.

2.4 Government Initiatives to Eradicate Child Malnutrition

Abundant of nutrition programs have been carried out in Malaysia since 1950s to tackle malnutrition issues among all age groups (NCCFN, 2016). The NPANM III 2016-2025 has illustrated lots of policies and activities that focus on reducing malnutrition for all age groups throughout these years (NCCFN, 2016). All of the activities implemented throughout Malaysia are guided and build up with reference to both global policies such as Global Nutrition Targets 2025 and SDGs 2030 and local policies and action plans such as the National Nutrition Policy of Malaysia 2005 and Ministry of Health Malaysia Strategic Plan 2016-2020 (NCCFN, 2016). The activities related to nutrition of children age 6 years old and below include Nutrition Surveillance, Nutrition Activities at Childcare Centres (TASKA), Rehabilitation Program for Malnourished Children (PPKZM) and Community Feeding Program (NCCFN, 2016).

Nutrition Surveillance is one of the nutritional activities that conducts continuously nationwide and aims to monitor the nutritional status of young children (NCCFN, 2016). The latest data collected focusing on child health and nutrition was the NHMS 2016 (IPH, 2016) that served as important indicators in developing and planning policies and programs for the improvement of nutritional and health status of young children (NCCFN, 2016). Another activity is the Nutrition Activities at Childcare Centres (TASKA), which involves a series of nutritional activities such as healthy eating promotion, involvement of nutritionists in menu or recipes development and training of caregivers to monitor nutritional status of children and healthy meal preparation in government childcare centres (NCCFN, 2016). These activities are also supported by some agencies such as Community Development Department (KEMAS) and Institutes for National Integration Studies and Training (IKLIN).

Besides that, a nutrition program that specifically designed and implemented for undernourished children age 6 months to 6 years from poor family, namely the Rehabilitation Program for Malnourished Children (*Program Pemulihan Kanak-Kanak Kekurangan Zat Makanan*; PPKZM) has been carried out since 1989 (NCCFN, 2016). Families that involved in this program are given basic food supplies in the form of food basket containing 14 basic foods such as rice, breakfast cereals, formula milk, biscuits, flour, multivitamins and eggs for at least 6 months (Ministry of Health Malaysia, 2015). Children are given immunisation and treatment if necessary and their parents are supported with nutritional education on child nutrition and hygiene to rehabilitate their undernourished children appropriately by nutritionists in the health clinics (NCCFN, 2016). This program later is used to develop another program called Community Feeding Program that targets different population, which is the marginalised group such as indigenous children (NCCFN, 2016).

Nevertheless, the implementation of PPKZM is questionable as limited success of weight gain among those children are not seen or reported (Wan Manan et al., 2019). The effort of government to ensure those food supplies are reachable to those undernourished children in poor families is also not recognized and reported. Indeed, the food baskets given in this program are also suspicious because the basic foods supplied also included multivitamins and special milk that indirectly persuading parents to use inappropriate and quick way to increase their children weight and disregarding the importance of proper diet.

Therefore, instead of merely supplying foods, nutrition programs that are going to implement throughout country may need an immediate change by employing more intensified actions and effective strategies such as focusing more on increasing nutritional knowledge and empowering the adoption of positive health and eating behaviour. Food being supply to those benefited groups should also well-considered and planned in advance to ensure the right messages being conveyed to them and indirectly promote healthy eating concept among them. For instance, stakeholders especially governors, policy makers and leaders should aware of the inequalities that already existed in the progress of combating undernutrition as discussed earlier.

There is a group of vulnerable people, the children from urban poor family that require further attention. Appropriate interventions are needed to be carried out in order to prevent childhood undernutrition. It is time for future strategy in tackling childhood undernutrition by focusing more on nutrition inequality in order to fasten the progress of reducing undernutrition in the country.

CHAPTER 3
METHODOLOGY

3.1 Introduction
Chapter 3 discusses on the methods, variables and measurements that will be used to conduct this study. This study is a mixed method study which will be carried out by using both qualitative and quantitative methods. Hence, this study will be conducted in two phases. Phase I of the study will involve qualitative research method to explore maternal feeding practices and also foods being fed to children of both positive deviant (PD) family and non-positive deviant (NPD) family. Phase II of the study will involve quantitative research method, which is a two-armed cluster randomized controlled trial to evaluate the effectiveness of a nutrition program using positive deviance approach in reducing undernutrition among urban poor children aged 3 to 5 years old in Kuala Lumpur. Each phase will be conducted by using different sample size, sampling method, data collection, measurements and data analysis. Hence, these elements will be discussed separately except for study location and population because the two phases of study will be conducted in same location within same population.

3.2 Study Location
This study will be conducted at low-cost PPR flat in Kuala Lumpur. Kuala Lumpur is chosen as it is fully urbanized (Department of Statistics Malaysia, 2010). Low cost PPR flats were built under the program of government, namely People’s Housing Project with the initiative to provide affordable housing for low income group whose household income were less than RM3000 in a month, which is also considered as the B40 group (Ministry of Housing and Local Government, 2018).

3.3 Phase 1
3.3.1 Study Design
In the first phase of study, identification and classification of families into PD family and NPD family based on children’s anthropometric measurements will be firstly carried out, followed by a qualitative study to explore the foods being fed to children and the maternal feeding practices of both positive deviant (PD) family (poor family with well-nourished children) and non-positive deviant (NPD) family (poor family with undernourished children) who live in low-cost PPR flats in Kuala Lumpur. This study will be conducted in the form of focus group discussion (FGD) with semi-structured interview.

3.3.2 Study Population
The study population is 3 to 5 years old children (index child) with their mothers who live together in low-cost PPR flat in Kuala Lumpur. Children aged 3 to 5 years old are recruited because if compared to younger children (0 to 24 months), older children are at higher risk of undernutrition (UNICEF, 2018). Mothers are the person who will receive intervention for behavioural change and children will be measured. Mother, rather than father or other caregivers are targeted because in general, mother is the primary caregiver of children (UNICEF, 2019). For household with more than one child aged 3 to 5 years old, the oldest child will be recruited as the index child. Older children tend to have more autonomy in their daily diet and are more susceptible to undernutrition (UNICEF, 2019).

The inclusion criteria for the selection of respondents in Phase I are:
a) Malaysian
b) Children aged 3 to 5 years old
c) Mothers aged above 18 years old

d) Living in public low-cost PPR flat
e) Monthly household income of less than RM3000 as it is one of the criteria in the application of residing in public low cost PPR flat (Ministry of Housing and Local Government, 2018)

The exclusion criteria for the selection of respondents in Phase I are:
a) Children who are taken care by other adults rather than mothers such as father, grandparents and caregivers
b) Mothers with mental disabilities
c) Children with history of chronic diseases including congenital heart disease, liver disease, renal failure or sickle cell disease and any congenital abnormalities
d) Children who are under treatment for communicable disease such as measles and chickenpox
e) Children with learning disabilities
f) Mother-child dyads involved in any other intervention or clinical research
g) Children who are overweight or obese

Other adults are being excluded because this study only aims at the general primary caretaker of young children who is the mother. Mothers with mental disabilities are not recruited as they might not be capable to follow fully the education and cooking sessions during the intervention. Children with chronic disease, under treatment of communicable diseases and learning disabilities will be excluded because their eating behaviours and nutritional status might be different from others which might lead to bias in results. Mother-child dyads involved in any other intervention or clinical research are excluded to avoid the confounding effect and also the accuracy of findings. Children with overweight or obesity will also be excluded as the intervention used in this study might not be able to rehabilitate all malnourished children at the same time.

3.3.2 Sample Size Estimation
The common recommendation for the number of informants in a FGD is ranged from 4 to 12 people (Bender & Ewbank, 1994; Kitzinger, 1995). Additionally, another study that reviewed the suitable number of samples in a focus group by using calculations had presented that the mean minimum number of informants in each group was 5.2 (Carlsen & Glenton, 2011). Informants will be recruited until saturation point is reached.

3.3.3 Sampling Method
A list of flats under People's Housing Project in Kuala Lumpur will be obtained from the Kuala Lumpur City Hall (*Dewan Bandaraya Kuala Lumpur; DBKL*). A list of households with children aged 3 to 5 years old will be obtained from the chairperson of the committee in the PPR flat. Two PPR flats with the highest and the second highest number of housing units will be selected via purposive sampling method. Household visit will be carried out in the flats. A total of 24 households with children aged 3 to 5 years old will be recruited from the two selected flats or more households will be recruited from the list to reach saturation point. To ensure equal chances of obtaining information from two types of family which are PD and NPD family, two focus groups will be recruited from the PD family while another two from the NPD family. The first six informants will be assigned as FGD 1, next six informants will be assigned as FGD 2 and so on according to the family types.

3.3.4 Data Collection
First phase of the study is estimated to be carried out in January and February 2021. After obtaining the list of households with under-five children in the selected PPR flats, household visit will be carried out. Informed consent and permission to conduct anthropometric

measurements on children will be obtained from mothers. Based on the anthropometric measurements of children, Positive Deviant family (PD Family) and Non-Positive Deviant family (NPD Family) will be identified. PD family refers to family with well-nourished under-five child who has normal growth indicators (-2SD ≤ Z ≤ +2 SD for WAZ, HAZ and WHZ). NPD family will be family who has undernourished child who is either underweight, stunting or wasting (WAZ/HAZ/WHZ< -2SD). Family who has an overweight or obese child will be excluded. If the family has a child who is stunted but overweight, the family will be excluded as well.

After identification, 12 informants will be recruited from PD families while another 12 informants will be recruited from NPD families. The informed consent and permission to audio-record will be obtained from mothers who will be the informants in respective FGDs. A short briefing will be given to the informants. After that, semi-structured face-to-face interview in the form of FGDs will be held in the community hall in the PPR. A total of four FGDs (two PD family only and two NPD family only) will be conducted to collect information. Researcher acts as a moderator to regulate whole process of the FGDs. Each informant in the group will be given a chance to answer each question. The ideal language to be used during the FGD process will be Malay. Each session of FGDs will be carried out for approximately 2 hours.

3.4.5 Measurements

An interview protocol will be prepared in advance which include preparation of a script and list of questions to be asked. The questions pertaining to maternal feeding practices are adapted from the Positive Deviance Inquiry, a guideline created by Sternin, Sternin and David (1998) to help in designing a nutrition program by using PD approach. The questions related to the foods being fed to children will be included in the section of maternal feeding practices. Other questions related to health seeking and child-care behaviours are excluded as this study will only focus on feeding practices that might influence child nutritional status.

3.4.6 Quality Control

To establish the trustworthiness of qualitative data that are going to collect in this study, peer debriefing will be done to create credibility. The interview protocol will be reviewed and revised repeatedly by researcher with discussion and advices from supervisory committee. To test the validity, a qualitative research strategy which is triangulation will be implemented. Triangulation is a strategy that enable researcher to develop comprehensive understanding on interested phenomena by merging information using multiple methods or data sources (Carter et al., 2014). In this phase of study, data source triangulation will be used whereby data will be collected to gain multiple perspectives and validation of data. Hence, the data will be collected from two different groups which are PD family and NPD family.

3.4.7 Data Analysis

The collected qualitative data will be analysed by using thematic analysis (Braun & Clarke, 2006; Nowell et al., 2017). The computer-assisted qualitative data analysis software (CAQDAS) that will be used is NVivo 12 (Melbourne, Australia). Firstly, the interview will be transcribed by using true verbatim transcription whereby each word will be written down and recorded even for irrelevant filters such as ‘yeah’, ‘uhm’ and others. After transcribing, the transcript will be read thoroughly in several times and be familiar with the contents. Some initial notes will be taken to search for meanings and patterns of data and prepare for coding process. Coding will be carried out by using highlighters whereby phases or main points will be highlighted in different colours corresponding to different codes. The data will be matched and categorized into different groups according to the characteristics represented by respective codes. Next, the codes will be turned and organised into themes or sub-themes by either

combining two relevant themes or splitting up a theme into two when the codes seem to be irrelevant. After generating themes and sub-themes, researcher will need to review the themes again to ensure they are representing extracted data accurately. Next step will be naming themes in relevant to their represented characteristics.

3.5 Phase 2

3.5.1 Study Design

A two-armed cluster randomized controlled trial will be carried out to determine the effectiveness of a nutrition program developed by using PD approach. Mother-child dyads will be randomly allocated into either intervention group or comparison group. Intervention group will be mother-child dyads whose children are undernourished and will need to participate in the PD program. The comparison group will also be mother-child dyads whose children are undernourished but will not receive any intervention. They will be provided with the developed educational materials used in the program after the program has been completed.

3.5.2 Study Population

The respondents' selection criteria in Phase II are almost similar as in Phase 1 except for the second inclusion criterion that is related to children. In Phase 2, only undernourished children (either underweight, stunting or wasting) aged 3 to 5 years old will be recruited in order to test the effectiveness of the intervention in this particular group. Hence, the inclusion criteria for the selection of respondents in Phase II are:

a) Malaysian
b) Undernourished children aged 3 to 5 years old
c) Mothers aged above 18 years old
d) Living in public low-cost PPR flat
e) Monthly household income of less than RM3000 (Ministry of Housing and Local Government, 2018)

The exclusion criteria in this phase are similar to the exclusion criteria in Phase 1.

3.5.3 Sample Size Estimation

A quasi-experimental study in Migori, Kenya conducted by Calvince et al. (2015) showed that at baseline, the mean and SD of weight-for-age (WAZ) of children aged 36 to 48 months in the intervention (using PD approach) and comparison groups were -1.38±1.75 and -1.12±1.32, respectively. At 6 months follow-up after graduation from the program, the mean and SD of WAZ of these children in the intervention and comparison groups were -0.65±0.58 and -1.30±1.54, respectively (Calvince et al., 2015). The sample size formula for an RCT comparing two groups of equal size (Florey, 1993; Noordzij et al., 2010) was used in this study:

$$n = \frac{2[(a + b)^2 \sigma^2]}{(\mu 1 - \mu 2)^2}$$

n = the sample size for each group
$\mu 1$ = mean WAZ of treatment group (-0.65)
$\mu 2$ = mean WAZ of control group (-1.3)
σ^2 = population variance (SD) (1.21)
a = conventional multiplier for alpha that set at 0.05 = 1.96
b = conventional multiplier for power that set at 0.80 = 0.842

According to the formula, the minimum sample size needed will be 54 for each group. It is estimated that there are around 10 households with undernourished children under five in a PPR flat. A previous study which is a cluster randomised trials focusing on the maternal and

child health had given an intra-cluster correlation coefficients (ICC) that ranged from 0 to 0.09, with most variables showed ICC less than 0.04 (Reading et al., 2000). Hence, it is assumed that the ICC to be used in calculating sample size in this study will be 0.03. By using equally sized cluster, the number of clusters required per arm, k, was calculated by using following formula (Hemming et al., 2011):

$$k = \frac{n_1[1 + (m - 1)\rho]}{m}$$

k = number of clusters required per arm
n_1 = sample size per arm under individual randomization (54)
m = cluster size per arm (10)
ρ = intra-cluster correlation coefficient (ICC) (0.03)

After calculating and rounding off, the number of clusters required per arm, k, is 7. Then, the required sample size per arm, n_c, is calculated by using following formula (Hemming et al., 2011):

$$n_c = \frac{n_1\,k[1 - \rho]}{(k - n_1 p)}$$

The sample size per arm obtained is 68. After taking into account the 20% non-response rate, the total sample size needed in this study will be 163 respondents. As such, the sample size required for each intervention and comparison group will be 82 respondents.

3.5.4 Sampling Method

The similar list of PPR flats and households in Phase 1 will be used, including the two PPR flats with the highest and second highest number of housing unit used in Phase 1. Eight PPR flats will be selected via simple random sampling method. Intervention and comparison groups will be recruited separately and randomly from 4 different PPR flats respectively. Household visit will be carried out again to conduct anthropometric measurements on children to obtain a list of households with undernourished children. Then, they will be randomly allocated into either intervention or comparison group.

3.5.3.1 Randomization and blinding

There are high possibilities that mother-child dyads in the intervention group are living in the similar flat as mother-child dyads of the comparison group, in which this might increase the chances of contamination. Recognising that, a cluster randomization design will be used to minimize the possibilities of contamination. Cluster randomization will be performed in a way that intervention group will be recruited from PPR flats where recruitment of comparison group will not be carried out in the same flats. By doing so, intervention and comparison groups will be recruited from different flats, in which this could minimize the risk of contamination. Prior to randomization, each mother-child dyad will be number coded by researcher. The coded list will then send to research assistant to perform random allocation. Mother-child dyads will be assigned into intervention or comparison group by using simple random sampling method with 1:1 allocation. The randomization list will later pass to researcher for invitation of mother-child dyads who fulfil the inclusion and exclusion criteria to participate in the program.

3.5.5 Development of Materials for Intervention

Before implementing intervention, information and materials will be gathered and developed in order to serve as necessary and successful tools for behavioural change in mothers of undernourished children. The methods that will be used are a combination of education on new knowledge and encouragement to practice new skill by conducting a 3-month program. It is

planned to conduct a nutrition program that will consist of education session with peer-led cooking session and rehabilitation session. It is also planned to collectively call the session as Nutrition Education and Rehabilitation Session (NERS). All materials will be developed by referring to published PD guidelines or education modules from previous PD studies (Sternin et al., 1998; The CORE group, 2002), government's nutrition-related guidelines such as Malaysian Dietary Guidelines for Children and Adolescents (NCCFN, 2013), recipes or menu from government or society such as Nutrition Society of Malaysia, and also findings related to PD behaviours and foods from PD family obtained from FGDs in Phase 1. It is planned to develop several topics as shown in Table 3.1.

Table 3.1: The proposed Nutrition Education and Rehabilitation Session (NERS) modules

Week	Modules
Week 1	Breast-feeding and weaning
Week 2	Eat a variety of food
Week 3	'*Suku-suku separuh*'
Week 4	Be physically active
Week 5	Carbohydrate
Week 6	Fish, egg, meat, poultry, legume, nut
Week 7	Milk and milk product
Week 8	Appropriate fat intake
Week 9	Limit salt/sugar/sauce
Week 10	Attain healthy weight
Week 11	Menu
Week 12	Educate children on nutrition

The education session will be delivered in both verbal and written short messages. The short messages of each topics will be written on a small card with simple language and concise sentences or presented as simple as possible in the form of leaflet, infographic or poster for the use of mothers. Some education sessions will be delivered in interactive ways such as through mini quiz and games. The delivery of new knowledge will be carried out for around 30 minutes with some explanations as the first activity in each session of weekly NERS.

For the peer-led cooking session, NERS menu will be planned in advance before implementation of the program according to the MDG for Children and Adolescents (NCCFN, 2013), the Recommended Nutrient Intake (RNI) (NCCFN, 2017) and recipes or menu from government or nutrition society such as Nutrition Society of Malaysia. The findings of PD foods from FGDs in Phase 1 will also be incorporated in NERS menu. According to the RNI, the daily macronutrient requirement of children is shown in Table 3.2 below.

Table 3.2: RNI for macronutrient among children aged 1 to 6 years according to gender

	Age	Energy (kcal/day) *based on recommended PAL 1.4	Carbohydrate (g/day) *50-65% of total energy	Protein (g/day)	Fat (g/day)
**Boys**	1-3 years	980	122.5-159.25	12	27-38
	4-6 years	1300	162.5-211.25	16	36-51
**Girls**	1-3 years	900	112.5-146.25	12	25-35
	4-6 years	1210	151.25-196.63	16	34-47

PAL: physical activity level
Source: NCCFN, 2017

Meals that will be prepared according to NERS menu in cooking session will serve as extra meal for children which will be scheduled halfway between other daily meal. Hence, menu that is going to prepare will provide additional calorie and nutrient to undernourished children so that they can attain enough nutrient for nutrient recovery and weight gain. NERS menu will be designed in a way that each child's portion will provide approximately 600 to 800 kcal and contain 20 to 30 grams of protein with reference of the RNI (NCCFN, 2017) It is planned to use a new menu in each week of NERS. Hence, a total of 12 menu will be planned in advanced. Each menu will be developed by incorporating the special PD foods discovered in Phase 1.

3.5.6 Implementation

3.5.6.1 Intervention Group

3.5.6.1.1 Nutrition Education and Rehabilitation Session (NERS)

By referring to previous studies and PD guidelines (Sternin et al., 1998), it is planned to conduct a 3-month nutrition program that will comprise education session with peer-led cooking session and rehabilitation session, namely the NERS. The NERS in the present study basically will be designed based on the findings obtained in Phase 1 and adapted the concept of original NERS as illustrated in the guidelines by Sternin et al. (1998).

According to guidelines by Sternin et al. (1998), the education and peer-led sessions should be held for 12-days continuously and the rest of the days in a month should be the rehabilitation session. However, considering that intense session might cause fatigue and also forgotten of knowledge learned, this study is planned to separate the 12 sessions so that participating mothers will be exposed to adequate nutrition knowledge in each week of each month. Hence, this study is planned to design a nutrition program for 3 months with a total of 12 sessions of NERS. It is planned that in each NERS, the education session to be carried out for about 2 hours for only one day in each week and a total of 4 days in one month. A 2-hour education session will consist of half hour education lesson and one and half hour peer-led cooking session. The rehabilitation session will be designed in the same way as previous guidelines, which will be the rest of the days following each education session until the next education session. The purpose is to initiate rehabilitation in undernourished children and at the same time allowing mothers to practice learned skill at home comfortably in a supportive environment (Sternin et al., 1998).

In contrast to previous NERS designated in guidelines, in this study, the education session in NERS will be conducted by researcher instead of volunteers from the community. The modules of education session in this study are modified according to government guidelines so that the nutrition knowledges provided in the process are suitable and fit the target group. Yet, the concept of peer-led cooking session is still being adopted. The cooking session will be led by volunteers from PD family instead of researcher because a demonstration of cooking session by peers with well-nourished children who live in the same environment as the participating mothers will be more convinced. This might increase the PD effect and further empower positive behavioural change in the mothers of undernourished children (Sternin et al., 1998).

For the peer-led cooking session, it is planned in a way that participating mothers involve actively from food preparation to food feeding with assistance of volunteers from PD families. Participating mothers will need to bring along their children during this session and feed their children with the prepared meal after cooking. The cooking demonstration will be led by volunteers from PD family and during the process, volunteers will involve and interact actively with participating mothers to assist them from food preparation to child feeding. Five to six mothers will be assigned in a group to prepare enough amount of meals for five to six children. Each group will be assigned with one volunteer to help them throughout the process.

The meal that is going to prepare in each cooking session will be prepared according to NERS menu and will be fed as snack or additional meal to children.

3.5.6.1.2 Schedule of NERS

A schedule of NERS will be set with volunteers and be discussed with participants which are the intervention group so that everyone is available and satisfied with the agreed time. A place will also be identified with the help of volunteers for NERS such as community hall in PPR flats.

3.5.6.1.3 Training of volunteers

Volunteers will be recruited from PD family identified in Phase I. Volunteers from PD family seems to be more convincingly than outsiders which indirectly enhance confidence of participating mothers. Fifteen or more volunteers will be recruited. Volunteers will play a main role in conducting peer-led cooking session and actively support NPD family to acquire new skill during the cooking session. Few discussion sessions will be conducted with volunteers before running the program to equip them with necessary knowledge and skill to help the participating mothers in the peer-led cooking session.

3.5.6.1.3 Growth monitoring

Mothers in the intervention group will be taught and trained to weigh their children so that they able to monitor their children nutritional status. The participating mothers need to weigh their children during each session on NERS before starting of education session. Weighing scale will be provided for participating mothers to measure their children during NERS. This is to increase the confidence of parent to feed their children appropriately and to show them that their children are gaining weight and becoming healthy under their care.

3.5.6.2 Comparison Group

After the 3-month program is completed, mothers from comparison groups will receive all the education materials and menu developed and used in the program.

3.5.7 Measurements

A Malay language self-administered questionnaire will be answered by the mothers to obtain information on socio-demographic characteristics, nutrition knowledge and food security status. Mothers will also be interviewed by researcher on the dietary intake of their children. Anthropometric measurements will be conducted by the researcher. The timeline for study measurements is presented in Table 3.3.

Table 3.3: Timeline for study measurements

No.	Measurements	Baseline (T0)	Immediate post-intervention (T1)	3-month post-intervention (T2)
1.	Socio-demographic background	✓		
2.	Height	✓	✓	✓
3.	Weight	✓	✓	✓
4.	Dietary intake	✓	✓	✓
5.	Food security status	✓	✓	✓
6.	Nutrition knowledge	✓	✓	✓

3.5.7.1 Socio-demographic background

Socio-demographic background of the mother-child dyads will be self-reported by the mothers, including information on child such as age, sex, ethnicity, and parent such as age, working status, occupation, education level, marital status, household size, monthly household income and financial assistance. The classification for occupation will refer to the Malaysia Standard Classification of Occupation (MASCO) 2013 (Ministry of Human Resources Malaysia, 2013). The monthly household income will be classified into three categories which are <RM1000, RM1000-1999, RM2000-RM2999 (IPH, 2020).

3.5.7.2 Anthropometric measurements

Height of the children will be measured to the nearest 0.1 cm by using a SECA Body Meter 213 (SECA, Germany) and weight will be measured to the nearest 0.1 kg by using a TANITA Digital Weight Scale HD662 (TANITA Corporation, Japan). The collected anthropometric data will be entered and analysed by using WHO Anthro Survey Analyser, a tool developed by World Health Organization (WHO) to analyse anthropometric survey data of children under five years of age based on weight and height (WHO, 2019). The weight-for-age z-score (WAZ), height-for-age z-score (HAZ) and weight-for-height z-score (WHZ) will be calculated by the software and will classify children into three categories which are underweight, stunting and wasting according to the WHO Child Growth Standards for children under age of 5 (WHO, 2006) as shown in Table 3.4.

Table 3.4: Classification of children under 5 according to z-score

Z-score values	Classification		
	**Weight for Age Z-score (WAZ)**	**Height for Age Z-score (HAZ)**	**Weight-for-Height Z-score (WHZ)**
> +3SD			Obesity
+2SD < Z ≤ +3SD			Overweight
-2SD ≤ Z ≤ +2 SD	Normal	Normal	Normal
-3SD ≤ Z < -2SD	Underweight	Stunting	Wasting
< -3SD	Severe underweight	Severe stunting	Severe wasting

Source: WHO (2006)

3.5.7.3 Diet assessment

The dietary intake of children will be assessed by using 24-hour dietary recall for three days, including two days in weekday and another day in weekend. Mothers will be asked to recall food intakes of their children at the three points of time which are at baseline, immediate post-intervention and 3-month post intervention.

Multiple pass 24-hour recall method will be used to limit the underreporting of nutrient intake (USDA, 2019). By using this method, researcher and mothers need to review the eating episodes several times to ensure information about food intake is given in detail and accurate. Firstly, mothers will be required to recall and list out foods and beverages being consumed by children in the past 24 hours. In the second pass, more detailed description such as description and preparation method will be obtained. In the final pass, researcher need to review the data collected and opt for further clarification such as additional eating occasions, food portion size, food labels and others with the aid of household measurement cups and spoons.

Dietary intake will be entered into a computer software, Nutritionist Pro™ Diet Analysis Software (Axxya, USA) by using the Nutrient Composition of Malaysian Foods database (Tee et al., 1997). In the case of absence of information of certain food items in the Malaysian database, other databases such as ASEAN Food Composition database (Institute of Nutrition Mahidol University, 2014) and Singapore food composition electronic database

(Health Promotion Board, 2011) will be used. Besides that, food label on packaged food will be referred or local recipes will be entered for analysis. Information such as total daily energy intake (kcal) and total daily macronutrients intake including carbohydrate, protein and fat as well as total daily micronutrient intake such as vitamin A, vitamin C, thiamine, riboflavin, niacin, calcium, iron, and sodium will be obtained. The nutrient intake of every child will then be compared with the RNI for Malaysians (NCCFN, 2017). The adequacy of macronutrient will be determined according to the Acceptable Macronutrient Distribution Range (AMDR) of macronutrients, which is 50-65% of total energy from carbohydrate, 10-20% of total energy from protein and 25-30% of total energy from fat. For micronutrient, either meet or exceed 100% of RNI will be considered as adequate intake (NCCFN, 2017).

Diet quality will be evaluated using Healthy Eating Index for Malaysians (HEI). The Healthy Eating Index for Malaysians (HEI) was developed by Lee et al. (2011) and had been validated (Goh & Norimah, 2012). The HEI consists of nine components, which are cereals and grains, vegetables, fruit, milk and milk products, poultry, meat and egg, fish, legumes, percentage of energy from fat and sodium intake (Lee et al., 2011). Each component is assessed to determine the compliance toward serving size as recommended in MDG for Children and Adolescents (NCCFN, 2013) as shown in Table 3.5. The score for each component is ranged from 0 (lack of compliance) to 10 (full compliance) and will be calculated by using the following formula: (actual serving consumed based on diet recall/recommended serving size based on MDG for Children and Adolescents) × 10.

Table 3.5: Criteria scoring for Healthy Eating Index for Malaysian Children

HEI component	Range of score	Criteria for minimum score of 0	Criteria for maximum score of 100
HEI composite score	0 to 100	-	-
Cereals and grains	0 to 10	0 serving	4 to 8 servings
Vegetables	0 to 10	0 serving	3 servings
Fruit	0 to 10	0 serving	2 servings
Milk and milk products	0 to 10	0 serving	1 to 3 servings
Poultry, meat and egg	0 to 10	0 serving	½ to 2 servings
Fish	0 to 10	0 serving	1 serving
Legumes	0 to 10	0 serving	½ to 1 serving
Percentage of energy from fat	0 to 10	≥ 35%	≤ 25%
Sodium	0 to 10	≥ 1900 mg	≤ 1200 mg

Source: Lee et al. (2011) and NCCFN (2013)

For percentage of energy from fat and sodium intake, the score will be calculated proportionately for the in-between whole number responses as shown in Table 3.6. Then, the composite score in percentage will be calculated using a formula (total score of 9 components/ 9 × 10) × 100%. The possible composite HEI score is ranged from 0 (low diet quality) to 100 (good diet quality), in which less than 51% indicated poor diet, 51 to 80% indicated diet requiring improvement, and more than 80% indicated good diet quality (Lee et al., 2011).

Table 3.6: Proportionate scoring for Healthy Eating Index for Malaysian Children

HEI score	0	1	2	3	4	5	6	7	8	9	10
% of energy from fat	≥ 35	34	33	32	31	30	29	28	27	26	≤ 25
Sodium (mg)	≥ 1900	1830	1760	1690	1620	1550	1480	1410	1340	1270	≤ 1200

3.5.7.4 Nutrition knowledge

To assess the nutrition knowledge level of mothers, questionnaire pertaining to nutrition knowledge will be taken from previous study which is '*Kajian KAP Makanan dan Pemakanan (1997)*' for adults (NCCFN, 1997). This questionnaire consists of 20 questions on nutritional knowledge which are related to calorie, carbohydrate, food pyramid, fat, protein and others. Each question answered correctly will be given one mark. The total scoring will be the total number of correct answers. Then, the percentage of score will be calculated by using formula (total score of correct answers/20 x 100%). Respondents whose percentage of score less than 50% will be classified as unsatisfactory, between 51% to 74.9% as moderate and more than 75% as satisfactory (NCCFN, 1997).

3.5.7.5 Food security status

The food security status will be determined by using the USDA six-item short form of the Household Food Security Survey Module (USDA, 2012). It contains six questions related to food security which is self-reported by mothers. The questionnaire was developed and modified from the 18-item U.S. Household Food Security Survey Module (Blumberg et al., 1999). The scoring will be calculated by summing up the total number of affirmative responses. Affirmative responses in this questionnaire will be "often" and "sometimes" or "almost every and some months". Respondents who report two or more affirmative responses will be classified as food insecurity and five or more responses will be categorised as hunger. The questionnaire was validated with reported item-score correlations ranging from 0.52 to 0.79 and a Cronbach's alpha value of 0.87 indicating good internal consistency reliability (Gulliford et al., 2004). It was previously used in Malaysia (Selamat et al., 2015).

3.5.8 Data collection

Data collection for Phase 2 of this study is expected to be conducted from March to September 2021. Figure 3.1 demonstrates the process of data collection. The findings regarding the PD practices and food obtained in the first phase will be used to develop the modules of education session and menu for peer-led cooking session. After developing materials for intervention, home visit to each identified household with children aged 3 to 5 years old will be carried out. A short brief on this study will be done followed by obtaining permission to conduct anthropometric measurements on children.

Based on the anthropometry measurements, mother-child dyads with undernourished children will be identified. These mother-child dyads with undernourished children will be randomly allocated into intervention and comparison groups. Second household visit will be carried out to invite and confirm participation. After obtaining informed consent from the mothers, anthropometric measurement will be conducted on children to serve as baseline information prior to intervention. Next, a self-administered questionnaire will be answered by mothers and dietary intake of their children will be recorded by using 24-hour diet recall at

baseline. Volunteers will also be identified and chosen from PD family. Volunteers will not participate in study as participants but will act as helpers throughout the program.

Intervention group will need to participate in a three-month program that consists of education lessons with peer-led cooking sessions and rehabilitation session, which will be collectively called as NERS. It is planned that the education session to be held once a week for 3 months and rehabilitation sessions to be the rest of day in a week after each education sessions. During the program, each education session will be held for about 2 hours, starting from education session, food preparation, cooking session and feeding session. Each session will consist of half-hour education session and one-and-half-bour peer-led cooking session. In each session, participating mothers will need to learn to measure their children with the aid of researcher and volunteers. Right after the intervention, anthropometric measurements on children and 24-hour diet recall will be conducted by researcher and the self-administered questionnaire will be distributed again to both intervention and comparison groups. At three months after the last rehabilitation session, anthropometric measurements and dietary practices (energy, macronutrient and micronutrient intakes, and diet quality) of children as well as food security status and nutrition knowledge for both intervention and comparison groups will be measured again.

Considering the pandemic of COVID-19, all SOP of COVID-19 such as wearing mask, temperature check and social distancing will be followed. If necessary, the number of people participate in a session during program, including mother-child dyads, volunteers and researcher, will be limited. For example, the number of participants in a session to be not more than 20 people. The same session to be held for several times for other participants.

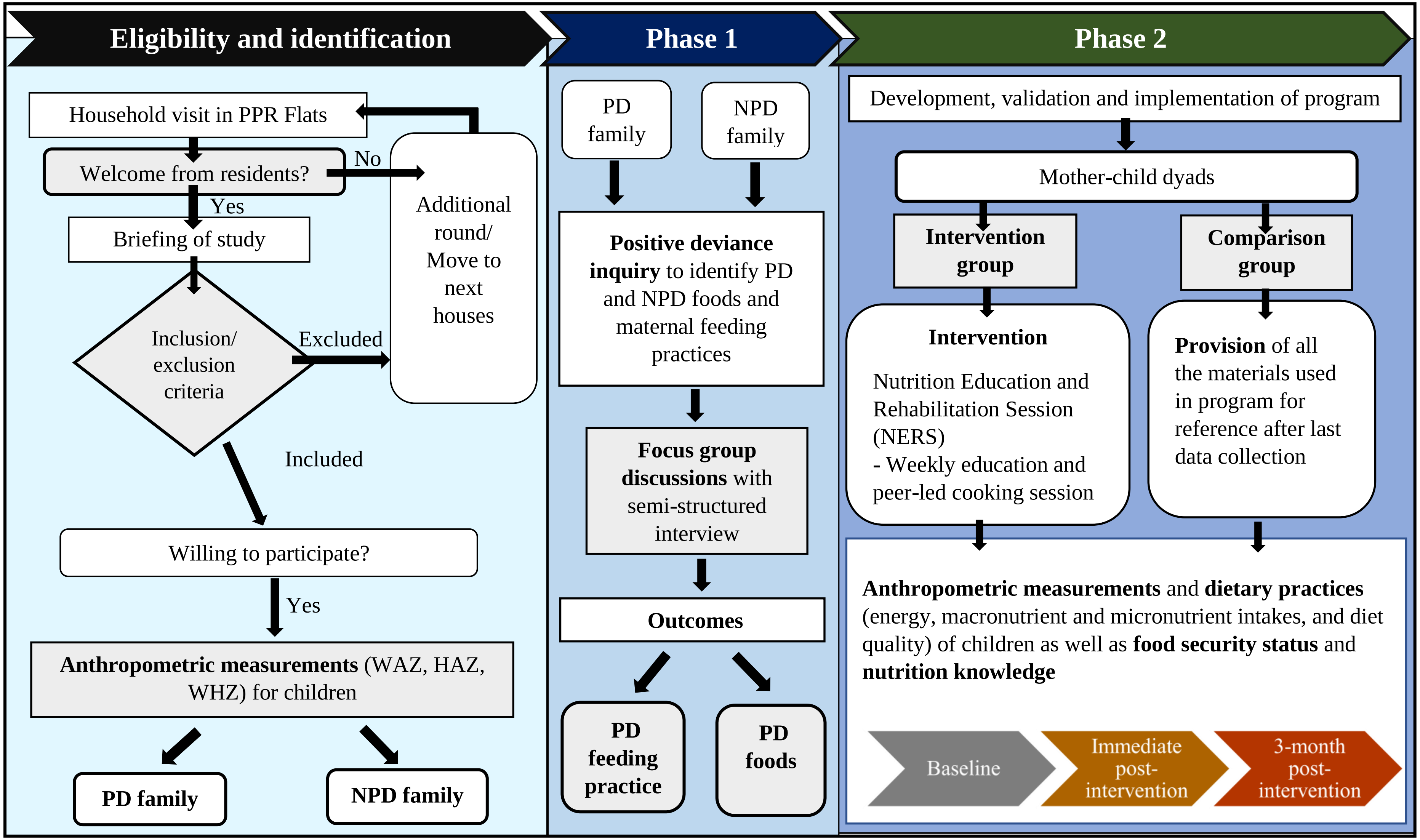

Figure 3.1: Flow chart of the data collection process

3.5.9 Data Analysis

The data collected will be entered and analysed by using IBM SPSS Statistics 26 (SPSS Inc., Chicago, IL, USA). Descriptive statistics will be used to summarise socio-demographic characteristics, WAZ, HAZ, WHZ, nutrient intake, food security status and nutrition knowledge level. Numerical data will be presented as mean (SD) or median (interquartile range; IQR) based on their normality distribution. The categorical data will be presented as frequency and percentage.

The normality distribution of data will be tested by using values for skewness and kurtosis between -2 and +2 (Field, 2017). The differences in anthropometric measurements (WAZ, HAZ, WHZ) and dietary practices (energy, macronutrient and micronutrient intakes, and diet quality) of children as well as food security status and nutrition knowledge of mothers between intervention and comparison groups will be tested using the independent-samples t-test. The effects of program on anthropometric measurements (WAZ, HAZ, WHZ) and dietary practices (energy, macronutrient and micronutrient intakes, and diet quality) of children as well as food security status and nutrition knowledge of mothers from intervention groups will be tested by using Generalized Linear Models (GLM). All p values to be used are two-tailed and the level of significance will be set at $p<0.05$.

3.6 Ethical consideration

Ethical approval is applied from Ethics Committee for Research Involving Human Subject (JKEUPM) of Universiti Putra Malaysia (Reference no: JKEUPM-2020-349). Permission to conduct the study in PPR flats will be obtained from Kuala Lumpur City Hall and management office of the selected PPR flats. Mother-child dyads will be included in this study, whereby the children will be under five. Hence, informed written consent will be obtained from their mothers. Their involvement is voluntary. They have the right to withdraw from this study at any time without providing any reason. All the information obtained and identity information provided will remain confidential throughout and after the study. Any child who has shown poor outcomes in either anthropometric measurements or questionnaire will be referred to health clinic for further action and management.

The safety of foods during the cooking demonstration session will be guaranteed by using only fresh ingredients. All participants will be required to wash hands before and after food preparation and cooking session. Participants will also need to wear necessary protective coverings such as apron, glove and hair net. Whenever the child feels not comfortable and with symptoms such as vomit and diarrhea after eating the meals provided in the program, mothers will be reminded to contact the researcher and the child will be sent to the nearby clinic or hospital as soon as possible for further treatment.

3.7 Incentives

Incentives will be given to all respondents, who participated in this study and successfully returned the answered questionnaire, as token of appreciation in the form of monetary or stationery.

3.8 Pilot test

The pilot test will be conducted in another two PPR flats in KL that is not targeted to participate in the study. This pilot test will be conducted in two phases. In the first phase, household visits and anthropometric measurements will be conducted to identify twelve households (6PD and 6NPD family). They will later participate in FGDs to test the feasibility of the interview protocol that contains pre-determined semi-structured questions in determining parental feeding practices and foods being fed to children. After that, in next phase, another group of 30 households with children aged 3 to 5 will be recruited. According to children's

anthropometric measurement, they will be assigned into either intervention group or comparison group. The findings from FGDs will be used to develop the education materials in advance. The NERS will be conducted with participation of intervention group to test the feasibility and determine the smoothness of program for one month. The information obtained from 24-hour diet recall will also be used to determine the validity of HEI by evaluating the correlation between composite score of the HEI and adequacy of nutrient intakes. The validity and reliability of self-administered questionnaire will also be pre-tested. The content validity will be evaluated by experts in nutrition field and Cronbach's alpha coefficient will be used to determine the internal consistency reliability of the questionnaires.

REFERENCES

Abdurahman, A. A., Mirzaei, K., Dorosty, A. R., Rahimiforoushani, A., & Kedir, H. (2016). Household food insecurity may predict underweightand Wasting among children aged 24–59 months. *Ecology of Food and Nutrition*, *55*(5), 456–472. https://doi.org/10.1080/03670244.2016.1207069

Abuya, B. A., Ciera, J., & Kimani-Murage, E. (2012). Effect of mother's education on child's nutritional status in the slums of Nairobi. *BMC Pediatrics*, *12,* 80. https://doi.org/10.1186/1471-2431-12-80

Aday, J., Hyden, A., Osking, J., & Tomedi, A. (2016). Hygiene, sanitation, and behaviors that produce positive deviant outcomes in childhood growth in rural eastern Kenya: A qualitative positive deviant investigation. *Annals of Global Health*, *82*(3), 437. https://doi.org/10.1016/j.aogh.2016.04.212

Adhikari, R. P., Shrestha, M. L., Acharya, A., & Upadhaya, N. (2019). Determinants of stunting among children aged 0–59 months in Nepal: findings from Nepal Demographic and Health Survey, 2006, 2011, and 2016. *BMC Nutrition*, *5,* 37. https://doi.org/10.1186/s40795-019-0300-0

Aguayo, V. M., Agarwal, V., Agnani, M., Das Agrawal, D., Bhambhal, S., Rawat, A. K., Gaur, A., Garg, A., Badgaiyan, N., & Singh, K. (2013). Integrated program achieves good survival but moderate recovery rates among children with severe acute malnutrition in India. *American Journal of Clinical Nutrition*, *98*(5), 1335–1342. https://doi.org/10.3945/ajcn.112.054080

Akombi, B. J., Agho, K. E., Hall, J. J., Wali, N., Renzaho, A. M. N., & Merom, D. (2017). Stunting, wasting and underweight in Sub-Saharan Africa: A systematic review. *International Journal of Environmental Research and Public Health*, *14*(8), 863. https://doi.org/10.3390/ijerph14080863

Albanna, B., & Heeks, R. (2019). Positive deviance, big data, and development: A systematic literature review. *Electronic Journal of Information Systems in Developing Countries*, *85*(1), e12063. https://doi.org/10.1002/isd2.12063

Ali, D., Saha, K. K., Nguyen, P. H., Diressie, M. T., Ruel, M. T., Menon, P., & Rawat, R. (2013). Household food insecurity is associated with higher child undernutrition in Bangladesh, Ethiopia, and Vietnam, but the effect is not mediated by child dietary diversity. *The Journal of Nutrition*, *143*(12), 2015–2021. https://doi.org/10.3945/jn.113.175182

Alirol, E., Getaz, L., Stoll, B., Chappuis, F., & Loutan, L. (2011). Urbanisation and infectious diseases in a globalised world. *The Lancet Infectious Diseases*, *11*(2), 131–141. https://doi.org/10.1016/S1473-3099(10)70223-1

Asian Development Bank. (2014). *Urban poverty in Asia*. https://doi.org/10.1177/0169796X0602300213

Astatkie, A. (2020). Dynamics of stunting from childhood to youthhood in Ethiopia: Evidence from the Young Lives panel data. *PLoS ONE*, *15*(2), e0229011. https://doi.org/10.1371/journal.pone.0229011

Ayana, A. B., Hailemariam, T. W., & Melke, A. S. (2015). Determinants of acute

malnutrition among children aged 6–59 months in Public Hospitals, Oromia region, West Ethiopia: a case–control study. *BMC Nutrition*, *1*(1). https://doi.org/10.1186/s40795-015-0031-9

Baker, J. L. (2008). *Urban poverty: A global view*. http://www-wds.worldbank.org/external/default/WDSContentServer/WDSP/IB/2008/03/24/000333037_20080324021722/Rendered/PDF/430280NWP0Glob10Box327344B01PUBLIC1.pdf%5Cnhttp://documents.worldbank.org/curated/en/954511468315832363/pdf/430280NWP0Glob10Box327344B0

Baumrind, D. (1966). Effects of authoritative parental control on child behavior. *Child Development*, *37*(4), 887–907.

Bender, D. E., & Ewbank, D. (1994). The focus group as a tool for health research: issues in design and analysis. *Health Transition Review*, *4*(1), 63–80. https://doi.org/10.2307/40652078

Bentley, M. E., Wasser, H. M., & Creed-Kanashiro, H. M. (2011). Responsive feeding and child undernutrition in low- and middle-income countries. *The Journal of Nutrition*, *141*, 502–507. https://doi.org/10.3945/jn.110.130005.Published

Benton, D. (2010). The influence of dietary status on the cognitive performance of children. *Molecular Nutrition and Food Research*, *54*(4), 457–470. https://doi.org/10.1002/mnfr.200900158

Bergmeier, H. J., Skouteris, H., Haycraft, E., Haines, J., & Hooley, M. (2015). Reported and observed controlling feeding practices predict child eating behavior after 12 months. *The Journal of Nutrition*, *145*(6), 1311–1316. https://doi.org/10.3945/jn.114.206268

Betebo, B., Ejajo, T., Alemseged, F., & Massa, D. (2017). Household food insecurity and its association with nutritional status of children 6–59 months of age in East Badawacho District, South Ethiopia. *Journal OfEnvironmental and Public Health*, 2017, 6373595. https://doi.org/10.1155/2017/6373595

Bhardwaj, P., Sharma, S., Raghav, P., & Kumar, D. (2016). Assessment of growth monitoring activities under Integrated Child Development Services (ICDS) in western Rajasthan. *International Journal of Medical Science and Public Health*, *5*(7), 1355. https://doi.org/10.5455/ijmsph.2016.02102015201

Birch, L. L., Fisher, J. O., Grimm-Thomas, K., Markey, C. N., Sawyer, R., & Johnson, S. L. (2001). Confirmatory factor analysis of the Child Feeding Questionnaire: A measure of parental attitudes, beliefs and practices about child feeding and obesity proneness. *Appetite*, *36*(3), 201–210. https://doi.org/10.1006/appe.2001.0398

Black, R. E., Victora, C. G., Walker, S. P., Bhutta, Z. A., Christian, P., De Onis, M., Ezzati, M., Grantham-Mcgregor, S., Katz, J., Martorell, R., & Uauy, R. (2013). Maternal and child undernutrition and overweight in low-income and middle-income countries. *The Lancet*, *382*(9890), 427–451. https://doi.org/10.1016/S0140-6736(13)60937-X

Blumberg, S. J., Bialostosky, K., Hamilton, W. L., & Briefel, R. R. (1999). The effectiveness of a Short Form of the Household Food Security Scale. *American Journal of Public Health*, *89*(8), 1231–1234. https://doi.org/10.2105/AJPH.89.8.1231

Boah, M., Azupogo, F., Amporfro, D. A., & Abada, L. A. (2019). The epidemiology of undernutrition and its determinants in children under five years in Ghana. *PLoS ONE*,

14(7), e0219665. https://doi.org/10.1371/journal.pone.0219665

Bong, Y., Asma, A. S., Abdul Majid, M., & Merican, A. F. (2015). Malaysian growth centiles for children under six years old. *Annals of Human Biology*, 42(2)108–15. https://doi.org/10.3109/03014460.2014.912679

Bork, K. A., & Diallo, A. (2017). Boys are more stunted than girls from early infancy to 3 Years of age in Rural Senegal. *The Journal of Nutrition*, *147*(5), 940–947. https://doi.org/10.3945/jn.116.243246

Braun, V., & Clarke, V. (2006). Using thematic analysis in psychology. *Qualitative Research in Psychology*, *3*(2), 77–101. https://doi.org/10.1191/1478088706qp063oa

Bullen, P. A. B. (2011). The positive deviance ⁄ hearth approach to reducing child malnutrition : Systematic review. *Tropical Medicine and International Health*, *16*(11), 1354–1366. https://doi.org/10.1111/j.1365-3156.2011.02839.x

Calvince, A. O., Were, G. M., & Khamasi, J. W. (2015). Impact evaluation of positive deviance hearth in Migori. *African Journal of Food, Agriculture, Nutrition and Development*, *15*(5), 10578–10596.

Carlsen, B., & Glenton, C. (2011). What about N? A methodological study of sample-size reporting in focus group studies. *BMC Medical Research Methodology*, *11*,26. https://doi.org/doi:10.1186/1471-2288-11-26

Carter, N., Bryant-lukosius, D., Dicenso, A., Blythe, J., & Neville, A. J. (2014). The use of triangulation in qualitative research nancy. *Oncology Nursing Forum*, *41*(5), 545–547. https://doi.org/10.1188/14.ONF.545-547

Chamhuri, S., Ahmed, F., Bashawir, A., & Mia, M. S. (2016). Urbanization and urban poverty in Malaysia : Consequences and vulnerability. *Journal of Applied Sciences*, *16*(4), 154–160. https://doi.org/10.3923/jas.2016.154.160

Chandrasekhar, S., Aguayo, V. M., Krishna, V., & Nair, R. (2017). Household food insecurity and children's dietary diversity and nutrition in India. Evidence from the comprehensive nutrition survey in Maharashtra. *Maternal and Child Nutrition*, *13*(Supp2), e12447. https://doi.org/10.1111/mcn.12447

Chaudhary, P., Agrawal, M., & Suman, R. L. (2018). Management of moderate acute malnutrition: Comparison of different approaches. *Asian Journal of Clinical Nutrition*, *10*(1), 47–57. https://doi.org/10.3923/ajcn.2018.47.57

Chhoun, P., Pal, K., Oy, S., Collins, C., Tout, S., & Yi, S. (2016). Social determinants of maternal and child undernutrition in Cambodia: A systematic review. *International Journal of Food and Nutritional Science*, *3*(2), 331–337. https://doi.org/10.15436/2377-0619.16.881

Ciliberto, M. A., Sandige, H., Ndekha, M. J., Ashorn, P., Briend, A., Ciliberto, H. M., & Manary, M. J. (2005). Comparison of home-based therapy with ready-to-use therapeutic food with standard therapy in the treatment of malnourished Malawian children: A controlled, clinical effectiveness trial. *American Journal of Clinical Nutrition*, *81*(4), 864–870. https://doi.org/10.1093/ajcn/81.4.864

Clark, H. R., Goyder, E., Bissell, P., Blank, L., & Peters, J. (2007). How do parents' child-feeding behaviours influence child weight? Implications for childhood obesity policy.

Journal of Public Health, *29*(2), 132–141. https://doi.org/10.1093/pubmed/fdm012

Cobbinah, P. B., Black, R., & Thwaites, R. (2013). Dynamics of poverty in developing countries: Review of poverty reduction approaches. *Journal of Sustainable Development*, *6*(9), 25–35. https://doi.org/10.5539/jsd.v6n9p25

Cobbinah, P. B., Erdiaw-Kwasie, M. O., & Amoateng, P. (2015a). Africa's urbanisation: Implications for sustainable development. *Cities*, *47*, 62–72. https://doi.org/10.1016/j.cities.2015.03.013

Cobbinah, P. B., Erdiaw-Kwasie, M. O., & Amoateng, P. (2015b). Rethinking sustainable development within the framework of poverty and urbanisation in developing countries. *Environmental Development*, *13*, 18–32. https://doi.org/10.1016/j.envdev.2014.11.001

Corsi, D. J., Mejía-Guevara, I., & Subramanian, S. V. (2016). Risk factors for chronic undernutrition among children in India: Estimating relative importance, population attributable risk and fractions. *Social Science and Medicine*, *157*, 165–185. https://doi.org/10.1016/j.socscimed.2015.11.014

Crouch, P., O'dea, J. A., & Battisti, R. (2007). Child feeding practices and perceptions of childhood overweight and childhood obesity risk among mothers of preschool children. *Nutrition and Dietetics*, *64*(3), 151–158. https://doi.org/10.1111/j.1747-0080.2007.00180.x

Cusick, S. E., & Georgieff, M. K. (2016). The role of nutrition in brain development: The golden opportunity of the "First 1000 Days." *Journal of Pediatrics, 175*, 16–21. https://doi.org/10.1016/j.jpeds.2016.05.013

De, P., & Chattopadhyay, N. (2019). Effects of malnutrition on child development: Evidence from a backward district of India. *Clinical Epidemiology and Global Health*, *7*(3), 439–445. https://doi.org/10.1016/j.cegh.2019.01.014

Demissie, S., & Worku, A. (2013). Magnitude and factors associated with malnutrition in children 6-59 months of age in Pastoral Community of Dollo Ado District, Somali Region, Ethiopia. *Science Journal of Public Health*, *1*(4), 175–183. https://doi.org/10.11648/j.sjph.20130104.12

Department of Statistic Malaysia. (2020). *Demographic statistics fourth quarter 2019*. https://www.statistics.gov.my/index.php?r=column/cthemeByCat&cat=77&bul_id=RlBvVUUwNVZnb0NkL2I2UnBjOUZhUT09&menu_id=OEY5SWtFSVVFVUpmUXEyaHppMVhEdz09

Department of Statistics Malaysia. (2010). Population distribution and basic demographic characteristics. In *Population and Housing Census of Malaysia 2010* (pp. 1–150).

Department of Statistics Malaysia. (2014). *Report of Household Income and Basic Amenities Survey 2014*. https://www.dosm.gov.my/v1/index.php?r=column/pdfPrev&id=aHhtTHVWNVYzTFBua2dSUlBRL1Rjdz09

Department of Statistics Malaysia. (2017). *Report of Household Income and Basic Amenities Survey 2016*. https://www.dosm.gov.my/v1/index.php?r=column/pdfPrev&id=RUZ5REwveU1ra1hGL21JWVlPRmU2Zz09%0Ahttps://www.dosm.gov.my/v1/index.php?r=column/pdfPrev&id=aHhtTHVWNVYzTFBua2dSUlBRL1Rjdz09

Department of Statistics Malaysia. (2019). *Migration survey report, Malaysia, 2018*. https://doi.org/10.1017/CBO9781107415324.004

Department of Statistics Malaysia. (2020). *Household Income and Basic Amenities Survey Report 2019*.

Dercon, S., & Singh, A. (2013). From nutrition to aspirations and self-efficacy: Gender bias over time among children in four countries. *World Development*, *45*, 31–50. https://doi.org/10.1016/j.worlddev.2012.12.001

Development Initiatives. (2018). *2018 Global Nutrition Report: Shining a light to spur action on nutrition*. https://doi.org/http://dx.doi.org/10.2499/9780896295643

Development Initiatives. (2020). *2020 Global Nutrition Report: Action on equity to end malnutrition*. https://globalnutritionreport.org/reports/2020-global-nutrition-report/

Dey, U., & Bisai, S. (2019). The prevalence of under-nutrition among the tribal children in India: A systematic review. *Anthropological Review*, *82*(2), 203–217. https://doi.org/10.2478/anre-2019-0014

Dickey, V. C., Pachón, H., Marsh, D. R., Lang, T. T., Claussenius, D. R., Dearden, K. A., Ha, T. T., & Schroeder, D. G. (2002). Implementation of nutrition education and rehabilitation programs (NERPs) in Viet Nam. *Food and Nutrition Bulletin*, *23*(4), 78–85. https://doi.org/10.1177/15648265020234s211

Eilander, A., Gera, T., Sachdev, H. S., Transler, C., Van Der Knaap, H. C. M., Kok, F. J., & Osendarp, S. J. M. (2010). Multiple micronutrient supplementation for improving cognitive performance in children: Systematic review of randomized controlled trials. *American Journal of Clinical Nutrition*, *91*(1), 115–130. https://doi.org/10.3945/ajcn.2009.28376

El Hioui, M., Ahami, A., Aboussaleh, Y., & Rusinek, S. (2016). The Relationship between Nutritional status and educational achievements in the rural school children of Morocco. *Journal of Neurology and Neurological Disorders*, *3*(1). https://doi.org/10.15744/2454-4981.3.101

El Kishawi, R. R., Soo, K. L., Abed, Y. A., & Wan Muda, W. A. M. (2016). Prevalence and associated factors for dual form of malnutrition in mother-child pairs at the same household in the Gaza strip-palestine. *PLoS ONE*, *11*(3), e0151494. https://doi.org/10.1371/journal.pone.0151494

Endris, N., Asefa, H., & Dube, L. (2017). Prevalence of malnutrition and associated factors among children in rural Ethiopia. *BioMed Research International*, *2019*, 8–10. https://doi.org/10.1155/2017/6587853

EPU. (2015). *Eleventh Malaysia Plan (2016-2020)*. https://www.epu.gov.my/en/rmk/eleventh-malaysia-plan-2016-2020

Ersino, G., Zello, G. A., Henry, C. J., & Regassa, N. (2018). Gender and household structure factors associated with maternal and child undernutrition in rural communities in Ethiopia. *PLoS ONE*, *13*(10), e0203914. https://doi.org/10.1371/journal.pone.0203914

Escobar, N. M. O., Márquez, I. A. V., Quiroga, J. A., Trujillo, T. G., González, F., Aguilar, M. I. G., & Escobar-Pérez, J. (2017). Using positive deviance in the prevention and control of MRSA infections in a Colombian hospital: A time-series analysis.

Epidemiology and Infection, *145*(5), 981–989. https://doi.org/10.1017/S095026881600306X

Eunice, M. J., Cheah, W. L., & Lee, P. Y. (2014). Factors influencing malnutrition among young children in a rural community of Sarawak. *Malaysian Journal of Nutrition*, *20*(2), 145–164.

Faith, M. S., Berkowitz, R. I., Stallings, V. A., Kerns, J., Storey, M., & Stunkard, A. J. (2004). Parental feeding attitudes and styles and child body mass index: Prospective analysis of a gene-environment interaction. *Pediatrics*, *114*(4), e429. https://doi.org/10.1542/peds.2003-1075-L

Faith, M. S., Heshka, S., Keller, K. L., Sherry, B., Matz, P. E., Pietrobelli, A., & Allison, D. B. (2003). Maternal-child feeding patterns and child body weight: Findings from a population-based sample. *Archives of Pediatrics and Adolescent Medicine*, *157*(9), 926–932. https://doi.org/10.1001/archpedi.157.9.926

FAO, IFAD, UNICEF, WFP, & WHO. (2018). *The state of food security and nutrition in the World 2018. Building climate resilience for food security and nutrition*. www.fao.org/publications

FAO, IFAD, UNICEF, WFP, & WHO. (2020). *The State of Food Security and Nutrition in the World 2020: Transforming food systems for affordable healthy diets*. https://doi.org/10.4060/ca9692en

Field, A. (2017). *Discovering Statistics Using IBM SPSS Statistics* (25th ed.). SAGE Publications Ltd.

Fink, G., Peet, E., Danaei, G., Andrews, K., McCoy, D. C., Sudfeld, C. R., Smith Fawzi, M. C., Ezzati, M., & Fawzi, W. W. (2016). Schooling and wage income losses due to early-childhood growth faltering in developing countries: National, regional, and global estimates. *American Journal of Clinical Nutrition*, *104*(1), 104–112. https://doi.org/10.3945/ajcn.115.123968

Fisher, J. O., Mitchell, D. C., Smiciklas-Wright, H., & Birch, L. L. (2002). Parental influences on young girls' fruit and vegetable, micronutrient, and fat intakes. *Journal of the American Dietetic Association*, *102*(1), 58–64. https://doi.org/10.1016/s0002-8223(02)90017-9

Florey, V. C. (1993). Sample size for beginners. *British Medical Journal*, *306*, 1181–1184. https://doi.org/10.1136/bmj.306.6886.1181

Fongar, A., Gödecke, T., & Qaim, M. (2019). Various forms of double burden of malnutrition problems exist in rural Kenya. *BMC Public Health*, *19*, 1543. https://doi.org/10.1186/s12889-019-7882-y

Fotso, J. C., Madise, N., Baschieri, A., Cleland, J., Zulu, E., Kavao Mutua, M., & Essendi, H. (2012). Child growth in urban deprived settings: Does household poverty status matter? At which stage of child development? *Health and Place*, *18*(2), 375–384. https://doi.org/10.1016/j.healthplace.2011.12.003

Galler, J. R., Bryce, C. P., Zichlin, M. L., Fitzmaurice, G., Eaglesfield, G. D., & Waber, D. P. (2012). Infant malnutrition is associated with persisting attention deficits in middle adulthood. *The Journal of Nutrition*, *142*(4), 788–794. https://doi.org/10.3945/jn.111.145441

Galler, J. R., Bryce, C. P., Zichlin, M. L., Waber, D. P., Exner, N., Fitzmaurice, G. M., & Costa, P. T. (2013). Malnutrition in the first year of life and personality at age 40. *Journal of Child Psychology and Psychiatry*, *54*(8), 911–919. https://doi.org/10.1111/jcpp.12066

Galler, J. R., Bryce, C., Waber, D. P., Zichlin, M. L., Fitzmaurice, G. M., & Eaglesfield, D. (2012). Socioeconomic outcomes in adults malnourished in the first year of life: A 40-year study. *Pediatrics*, *130*(1), 1–7. https://doi.org/10.1542/peds.2012-0073

Galloway, A. ., LauraFiorito, Lee, Y., & L.Birch, L. (2005). Parental pressure, dietary patterns, and weight status among girls who are "picky eaters." *Journal of the American Dietetic Association*, *105*(4), 541–548. https://doi.org/10.1016/j.jada.2005.01.029

GCPH. (2012). *Putting asset based approaches into practice: identification, mobilisation and measurement of assets July*. https://www.gcph.co.uk/assets/0000/3433/GCPHCS10forweb_1_.pdf

Gebre, A., Surender Reddy, P., Mulugeta, A., Sedik, Y., & Kahssay, M. (2019). Prevalence of malnutrition and associated factors among under-five children in Pastoral Communities of Afar Regional State, Northeast Ethiopia: A community-based cross-sectional study. *Journal of Nutrition and Metabolism*, 2019, 9187609. https://doi.org/10.1155/2019/9187609

Gewa, C. A., & Yandell, N. (2012). Undernutrition among Kenyan children: Contribution of child, maternal and household factors. *Public Health Nutrition*, *15*(6), 1029–1038. https://doi.org/10.1017/S136898001100245X

Ghazi, H. F., Isa, Z. M., Aljunid, S., Tamil, A. M., & Abdalqader, M. A. (2012). Nutritional status, nutritional habit and breakfast intake in relation to IQ among primary school children in Baghdad city, Iraq. *Pakistan Journal of Nutrition*, *11*(4), 379–382. https://doi.org/10.3923/pjn.2012.379.382

Ghodsi, D., Rashidian, A., Omidvar, N., Eini-Zinab, H., Raghfar, H., & Ebrahimi, M. (2018). Process evaluation of a national, community-based, food supplementary programme for improving the nutritional status of children in Iran. *Public Health Nutrition*, *21*(15), 2811–2818. https://doi.org/10.1017/S1368980018001696

Glanz, K., Rimer, B. k., & Viswanath, K. (2008). *Health behavior and health education: Theory, research and practice* (4th ed.). Jossey-Bass. https://d1wqtxts1xzle7.cloudfront.net/49289960/Health_Behavior___Health_Education_book_4th_Ed.pdf?1475413105=&response-content-disposition=inline%3B+filename%3DHealth_Behavior_and_Health_Education_boo.pdf&Expires=1597219668&Signature=SxduCvvaQnGC75AkzCn5s

Goh, H., & Norimah, A. (2012). Validation of Healthy Eating Index (HEI) for Malaysian adults. *Proceedings of 27th Scientific Conference of the Nutrition Society of Malaysia*, 24–25.

Gong, P., Liang, S., Carlton, E. J., Jiang, Q., Wu, J., Wang, L., & Remais, J. V. (2012). Urbanisation and health in China. *The Lancet*, *379*(9818), 843–852. https://doi.org/10.1016/S0140-6736(11)61878-3

Gregory, J. E., Paxton, S. J., & Brozovic, A. M. (2010). Maternal feeding practices, child eating behaviour and body mass index in preschool-aged children: A prospective

analysis. *International Journal of Behavioral Nutrition and Physical Activity, 7*(55). https://doi.org/10.1186/1479-5868-7-55

Gulliford, M. C., Mahabir, D., & Rocke, B. (2004). Reliability and validity of a short form household food security scale in a Caribbean community. *BMC Public Health, 4, 22*. https://doi.org/10.1186/1471-2458-4-22

Gyampoh, S., Otoo, G. E., & Aryeetey, R. N. O. (2014). Child feeding knowledge and practices among women participating in growth monitoring and promotion in Accra, Ghana. *BMC Pregnancy and Childbirth, 14*, 180. http://www.biomedcentral.com/1471-2393/14/180%5Cnhttp://ovidsp.ovid.com/ovidweb.cgi?T=JS&PAGE=reference&D=emed12&NEWS=N&AN=2014388816

Haycraft, E., Karasouli, E., & Meyer, C. (2017). Maternal feeding practices and children's eating behaviours: A comparison of mothers with healthy weight versus overweight/obesity. *Appetite*, *116*, 395–400. https://doi.org/10.1016/j.appet.2017.05.033

Health Promotion Board. (2011). *Energy & Nutrient Composition of Food*. https://focos.hpb.gov.sg/eservices/ENCF/

Hemming, K., Girling, A. J., Sitch, A. J., Marsh, J., & Lilford, R. J. (2011). Sample size calculations for cluster randomised controlled trials with a fixed number of clusters. *BMC Medical Research Methodology*, *11*, 102. https://doi.org/10.1186/s12874-017-0292-x

Herington, M. J., & Fliert, E. Van De. (2017). Positive deviance in theory and practice : A conceptual review. *Deviant Behavior*, *39*(5), 664-678. https://doi.org/10.1080/01639625.2017.1286194

Hien, N. N., & Hoa, N. N. (2009). Nutritional status and determinants of malnutrition in cildren under three years of age in Nghean, Vietnam. In *Pakistan Journal of food Science, 8(*7), 958–964.

Hirani, S. A. A. (2012). Malnutrition in young Pakistani children. *Journal of Ayub Medical College*, *24*(2), 150–153.

Hughes, S. O., Power, T. G., Orlet Fisher, J., Mueller, S., & Nicklas, T. A. (2005). Revisiting a neglected construct: Parenting styles in a child-feeding context. *Appetite*, *44*(1), 83–92. https://doi.org/10.1016/j.appet.2004.08.007

Huybregts, L., Houngbé, F., Salpéteur, C., Brown, R., Roberfroid, D., Ait-Aissa, M., & Kolsteren, P. (2012). The effect of adding Ready-to-Use Supplementary Food to a general food distribution on child nutritional status and morbidity: A cluster-randomized controlled trial. *PLoS Medicine*, *9*(9), e1001313. https://doi.org/10.1371/journal.pmed.1001313

Ihab, A. N., Rohana, A. J., & Wan Manan, W. M. (2015). Concept and measurements of household food insecurity and its impact on malnutrition : A review. *International Medical Journal*, *22*(6), 509–516.

Ihab, A. N., Rohana, J., Wan Manan, W. M., Wan Suriati, W. N., Zalilah, M. S., & Mohamed Rusli, A. (2013). The coexistence of dual form of malnutrition in a sample of rural Malaysia. *International Journal of Preventive Medicine*, *4*(6), 690–699.

Ihab, A. N., Rohana, J., Wan Manan, W. M., Wan Suriati, W. N., Zalilah, M. S., & Mohamed

Rusli, A. (2014). Association between household food insecurity and nutritional outcomes among children in Northeastern of Peninsular Malaysia. *Nutrition Research and Practice*, *8*(3), 304. https://doi.org/10.4162/nrp.2014.8.3.304

Ihab, A. N., Rohana, J., Wan Manan, W. M., Wan Suriati, W. N., Zalilah, M. S., & Mohamed Rusli, A. (2015). Assessment of Food Insecurity and Nutritional Outcomes in Bachok. *Journal of Nutrition & Food Sciences*, *5*(3), 1–20. https://doi.org/10.4172/2155-9600.1000373

IIPS, & ICF. (2017). *National Family Health Survey (NFHS-4), 2015-16*. http://rchiips.org/NFHS/NFHS-4Reports/India.pdf

Ijarotimi, O. S. (2013). Determinants of childhood malnutrition and consequences in developing countries. *Current Nutrition Reports*, *2*(3), 129–133. https://doi.org/10.1007/s13668-013-0051-5

Inamahoro, C., Kiguli, J., Makumbi, F. E., Wamuyu-Maina, G., & Wamani, H. (2017). Nutritional recovery outcome among moderately malnourished children in communities implementing positive deviance - Hearth or hommunity health workers ' nutrition promotion approaches in Karusi and Kirundo Provinces , Burundi. *Journal of Science & Sustainable Development*, *6*(1), 5–19. https://doi.org/10.4314/jssd.v6i1.1

Institute of Nutrition Mahidol University. (2014). *ASEAN Food Composition Database* (Electronic version 1). http://www.inmu.mahidol.ac.th/aseanfoods/doc/ASEAN_FCD_V1_2014.pdf

IPH. (2015). *National Health and Morbidity Survey 2015 (NHMS 2015). Vol. II: Non-communicable diseases, risk factors & other health problems*. http://iku.moh.gov.my/images/IKU/Document/REPORT/nhmsreport2015vol2.pdf

IPH. (2016). *National Health and Morbidity Survey 2016: Maternal and Child Health. Volume II: Findings*.

IPH. (2020). *National Health and Morbidity Survey (NHMS) 2019: Non-communicable diseases, healthcare demand, and health literacy- Technical Report Volume I*.

Irarrázaval, B., Barja, S., Bustos, E., Doirsaint, R., Senethmm, G., Guzmán, M., & Uauy, R. (2018). Influence of feeding practices on malnutrition in Haitian infants and young children. *Nutrients*, *10*(3), 382. https://doi.org/10.3390/nu10030382

Isanaka, S., Roederer, T., Djibo, A., Luquero, F. J., Nombela, N., Guerin, P. J., & Grais, R. F. (2010). Reducing wasting in young children with preventive supplementation: A cohort study in Niger. *Pediatrics*, *126*(2), e442–e450. https://doi.org/10.1542/peds.2009-2814

Jesmin, A., Yamamoto, S. S., Malik, A. A., & Haque, M. A. (2011). Prevalence and determinants of chronic malnutrition among preschool children: A cross-sectional study in Dhaka City, Bangladesh. *Journal of Health, Population and Nutrition*, *29*(5), 494–499. https://doi.org/10.3329/jhpn.v29i5.8903

Johannsen, D. L., Johannsen, N. M., & Specker, B. L. (2006). Influence of parents' eating behaviors and child feeding practices on children's weight status. *Obesity*, *14*(3), 431–439. https://doi.org/10.1038/oby.2006.57

Joshi, P. (2018). Malnutrition in children: A serious public health issue in Nepal. *Health Prospect*, *11*, 61–62. https://doi.org/10.3126/hprospect.v11i0.7439

Kanani, S., & Popat, K. (2012). Growing normally in an urban environment: Positive deviance among slum children of Vadodara, India. *Indian Journal of Pediatrics*, *79*(5), 606–611. https://doi.org/10.1007/s12098-011-0612-9

Kang, Y., Kim, S., Sinamo, S., & Christian, P. (2016). Effectiveness of a community-based nutrition programme to improve child growth in rural Ethiopia : A cluster randomized trial. *Maternal & Child Nutrition*, 1–15. https://doi.org/10.1111/mcn.12349

Kavosi, E., Rostami, Z. H., Kavosi, Z., Nasihatkon, A., Moghadami, M., & Heidari, M. (2014). Prevalence and determinants of under-nutrition among children under six: A cross-sectional survey in Fars province, Iran. *International Journal of Health Policy and Management*, *3*(2), 71–76. https://doi.org/10.15171/ijhpm.2014.63

Khambalia, A. Z., Lim, S. S., Gill, T., & Bulgiba, A. M. (2012). Prevalence and sociodemographic factors of malnutrition among children in Malaysia. *Food and Nutrition Bulletin*, *33*(1), 31–42. https://doi.org/10.1177/156482651203300103

Khan, A., Kabir, I., Ekström, E. C., Sling-Monemi, K., Alam, D., Frongillo, E. A., Yunus, M., Arifeen, S., & Persson, L. K. (2011). Effects of prenatal food and micronutrient supplementation on child growth from birth to 54 months of age: A randomized trial in Bangladesh. *Nutrition Journal*, *10*(1), 1–11. https://doi.org/10.1186/1475-2891-10-134

Khera, R., Jain, S., Lodha, R., & Ramakrishnan, S. (2014). Gender bias in child care and child health: Global patterns. *Archives of Disease in Childhood*, *99*(4), 369–374. https://doi.org/10.1136/archdischild-2013-303889

Khoo, S. L., Mohamad, S. S., Gopal, P. S., Malek, N. M., & Hamat, Z. (2018). Urban poverty alleviation strategies from multidimensional and multi-ethnic perspectives: Evidences from Malaysia. *Kajian Malaysia*, *36*(2), 43–68. https://doi.org/10.21315/km2018.36.2.3

Kinyoki, D. K., Berkley, J. A., Moloney, G. M., Kandala, N. B., & Noor, A. M. (2015). Predictors of the risk of malnutrition among children under the age of 5 years in Somalia. *Public Health Nutrition*, *18*(17), 3125–3133. https://doi.org/10.1017/S1368980015001913

Kitzinger, J. (1995). Qualitative Research: Introducing focus groups. *BMJ, 311*, 299–302. https://doi.org/10.1136/bmj.311.7000.299

Koo, H. C., Poh, B. K., & Talib, R. A. (2018). The GReat-child™ trial: A quasi-experimental intervention on whole grains with healthy balanced diet to manage childhood obesity in Kuala Lumpur, Malaysia. *Nutrients*, *10*(2). https://doi.org/10.3390/nu10020156

Kosaka, S., & Umezaki, M. (2017). A systematic review of the prevalence and predictors of the double burden of malnutrition within households. *British Journal of Nutrition*, *117*(8), 1118–1127. https://doi.org/10.1017/S0007114517000812

Lackovich-Van Gorp, A. (2017). Unearthing local forms of child protection: Positive deviance and abduction in Ethiopia. *Action Research*, *15*(1), 39–52. https://doi.org/10.1177/1476750316679240

Langendorf, C., Roederer, T., de Pee, S., Brown, D., Doyon, S., Mamaty, A. A., Touré, L. W. M., Manzo, M. L., & Grais, R. F. (2014). Preventing Acute Malnutrition among Young Children in Crises: A Prospective Intervention Study in Niger. *PLoS Medicine*, *11*(9), e1001714. https://doi.org/10.1371/journal.pmed.1001714

Lapping, K., Marsh, D. R., Rosenbaum, J., Swedberg, E., Sternin, J., Sternin, M., & Schroeder, D. G. (2002). The positive deviance approach : Challenges and opportunities for the future. *Food and Nutrition Bulletin, 23*(4), 128–135.

Le Nguyen, B. K., Le Thi, H., Nguyen Do, V. A., Tran Thuy, N., Nguyen Huu, C., Thanh Do, T., Deurenberg, P., & Khouw, I. (2013). Double burden of undernutrition and overnutrition in Vietnam in 2011: Results of the SEANUTS study in 0.5-11-year-old children. *British Journal of Nutrition*, *110* (Supp3), S45-56. https://doi.org/10.1017/S0007114513002080

Lee, H., & Keller, K. L. (2012). Children who are pressured to eat at home consume fewer high-fat foods in laboratory test meals. *Journal of the Academy of Nutrition and Dietetics*, *112*(2), 271–275. https://doi.org/10.1016/j.jada.2011.10.021

Lee, T. T., Norimah, A. K., & Safiah, M. Y. (2011). Development of Healthy Eating Index for Malaysian adults. *Proceedings of 26th Scientific Conference and Annual General Meeting of the Nutrition Society of Malaysia*, 101–102.

Lee, Z. L., Gan, W. Y., Lim, P. Y., Hasan, R., & Lim, S. Y. (2020). Associations of nutritional status, sugar and second-hand smoke exposure with dental caries among 3-to 6-year old Malaysian pre-schoolers: A cross-sectional study. *BMC Oral Health*, *20*, 164. https://doi.org/10.1186/s12903-020-01152-0

Maccoby, E. E., & Martin, J. A. (1983). Socialization in the context of thefFamily: Parent-child interaction. In P. H. Mussen & E. M. Hetherington (Eds.), *Handbook of child psychology: Vol. 4: Socialization, personality and social development* (4th ed., pp. 1–101). Wiley.

Mais, L. A., Warkentin, S., Latorre, M. do R. D. de O., Carnell, S., & Taddei, J. A. A. de C. (2017). Parental feeding Practices among Brazilian school-aged children: Associations with parent and child characteristics. *Frontiers in Nutrition*, *4*(March), 1–10. https://doi.org/10.3389/fnut.2017.00006

Maitra, C. (2018). *A review of studies examining the link between food insecurity and malnutrition*. http://www.fao.org/3/CA1447EN/ca1447en.pdf

Majamanda, J., Maureen, D., Munkhondia, T. M., & Carrier, J. (2014). The effectiveness of community-based nutrition education on the nutrition status of under-five children in developing countries. A systematic review. *Malawi Medical Journal*, *26*(4), 115–118.

Martorell, R., & Zongrone, A. (2012). Intergenerational influences on child growth and undernutrition. *Paediatric and Perinatal Epidemiology*, *26*(Suppl 1), 302–314. https://doi.org/10.1111/j.1365-3016.2012.01298.x

Mary, S. (2018). How much does economic growth contribute to child stunting reductions? *Economies*, *6*(4). https://doi.org/10.3390/economies6040055

McGovern, M. E., Krishna, A., Aguayo, V. M., & Subramanian, S. V. (2017). A review of the evidence linking child stunting to economic outcomes. *International Journal of Epidemiology*, *46*(4), 1171–1191. https://doi.org/10.1093/ije/dyx017

McPhie, S., Skouteris, H., Daniels, L., & Jansen, E. (2014). Maternal correlates of maternal child feeding practices: A systematic review. *Maternal and Child Nutrition*, *10*(1), 18–43. https://doi.org/10.1111/j.1740-8709.2012.00452.x

Mekonen, J., Addisu, S., & Mekonnen, H. (2019). Prevalence and associated factors of chronic undernutrition among under five children in Adama town, Central Ethiopia: A cross-sectional study design. *BMC Research Notes*, *12*, 532. https://doi.org/10.1186/s13104-019-4552-1

Menon, P., Bamezai, A., Subandoro, A., Ayoya, M. A., & Aguayo, V. (2015). Age-appropriate infant and young child feeding practices are associated with child nutrition in India: Insights from nationally representative data. *Maternal and Child Nutrition*, *11*(1), 73–87. https://doi.org/10.1111/mcn.12036

Merita, M., Sari, M. T., & Hesty, H. (2017). The positive deviance of feeding practices and carring with nutritional status of toddler among poor families. *KEMAS*, *13*(1), 106–112. https://doi.org/10.15294/kemas.v13i1.7919

Ministry of Finance Malaysia. (2019). *Budget 2020*.

Ministry of Health Malaysia. (2015). *Guidelines for the Programme of Rehabilitation of Malnourished Children*.

Ministry of Housing and Local Government. (2018). *People's Housing Program*.

Ministry of Human Resources Malaysia. (2013). Malaysia Standard Classification of Occupations. In *Malaysia Standard Classification of Occupations* (Issue 1). http://static.jobsmalaysia.gov.my/html/jobsm/masco/ms/Prinsip-pengelasan-pekerjaan.pdf

Mohd Nasir, M. T., Norimah, A. K., Hazizi, A. S., Nurliyana, A. R., Loh, S. H., & Suraya, I. (2012). Child feeding practices, food habits, anthropometric indicators and cognitive performance among preschoolers in Peninsular Malaysia. *Appetite*, *58*(2), 525–530. https://doi.org/10.1016/j.appet.2012.01.007

Mohseni, M., Aryankhesal, A., & Kalantari, N. (2018). Prevalence of malnutrition among Iran's under five-year-old children and the related factors: A systematic review and meta-analysis. *Iranian Journal of Pediatrics*, *28*(1), 1–9. https://doi.org/10.5812/ijp.9189

Moradi, S., Mirzababaei, A., Mohammadi, H., Moosavian, S. P., Arab, A., Jannat, B., & Mirzaei, K. (2019). Food insecurity and the risk of undernutrition complications among children and adolescents: A systematic review and meta-analysis. *Nutrition*, *62*, 52–60. https://doi.org/10.1016/j.nut.2018.11.029

Morgan, D. L. (1996). Focus Groups. *Annual Review of Sociology*, *22*, 129–152. https://doi.org/10.1016/B0-12-369398-5/00039-6

Motbainor, A., Worku, A., & Kumie, A. (2015). Stunting is associated with food diversity while wasting with food insecurity among underfive children in East and West Gojjam Zones of Amhara Region, Ethiopia. *PLoS ONE*, *10*(8), e0133542. https://doi.org/10.1371/journal.pone.0133542

Mulu, E., & Mengistie, B. (2017). Household food insecurity and its association with nutritional status of under five children in Sekela District, Western Ethiopia: a comparative cross-sectional study. *BMC Nutrition*, *3*, 35. https://doi.org/10.1186/s40795-017-0149-z

Mutisya, M., Kandala, N. B., Ngware, M. W., & Kabiru, C. W. (2015). Household food

(in)security and nutritional status of urban poor children aged 6 to 23 months in Kenya Global health. *BMC Public Health*, *15*, 1052. https://doi.org/10.1186/s12889-015-2403-0

Mwase, I., Mutoro, A., Owino, V., Garcia, A. L., & Wright, C. M. (2016). Poor infant feeding practices and high prevalence of malnutrition in urban slum child care centres in Nairobi: A pilot study. *Journal of Tropical Pediatrics*, *62*, 46–54. https://doi.org/10.1093/tropej/fmv071

NCCFN. (1997). *Kajian KAP Makanan dan Pemakanan (1997) for adults*.

NCCFN. (2013). *Malaysian Dietary Guidelines for Children and Adolescents*. http://www.moh.gov.my/images/gallery/Garispanduan/MDG Children and Adolescents Summary.pdf

NCCFN. (2016). *National Plan of Action for Nutrition of Malaysia (2016-2025)*. National Coordinating Committee on Food and Nutrition (NCCFN).

NCCFN. (2017). *Recommended Nutrient Intake for Malaysians (RNI)*.

Negash, C., Whiting, S. J., Henry, C. J., Belachew, T., & Hailemariam, T. G. (2015). Association between maternal and child nutritional status in Hula, rural Southern Ethiopia: A cross sectional study. *PLoS ONE*, *10*(11), e0142301. https://doi.org/10.1371/journal.pone.0142301

Nga, T. T., Nguyen, M., Mathisen, R., Hoa, D. T. B., Minh, N. H., Berger, J., & Wieringa, F. T. (2013). Acceptability and impact on anthropometry of a locally developed Ready-to-use therapeutic food in pre-school children in Vietnam. *Nutrition Journal*, *12*(1), 1–8. https://doi.org/10.1186/1475-2891-12-120

Nieto-Sanchez, C., Baus, E. G., Guerrero, D., & Grijalva, M. J. (2015). Positive deviance study to inform a chagas disease control program in southern Ecuador. *Memorias Do Instituto Oswaldo Cruz*, *110*(3), 299–309. https://doi.org/10.1590/0074-02760140472

Nishat, N., & Batool, I. (2011). Effect of " Positive Hearth Deviance " on feeding practices and underweight prevalence among children aged 6-24 months in Quetta district , Pakistan : A comparative cross sectional study. *Sri Lanka Journal of Child Health, 40*, 57–62.

Noordzij, M., Tripepi, G., Dekker, F. W., Zoccali, C., Tanck, M. W., & Jager, K. J. (2010). Sample size calculations: Basic principles and common pitfalls. *Nephrology Dialysis Transplantation*, *25*(5), 1388–1393. https://doi.org/10.1093/ndt/gfp732

Norliza, A., Zalilah, M. S., Firdaus, M., & Lye, M.-S. (2018). Family-based intervention using face-to- face sessions and social media to improve Malay primary school children's adiposity: a randomized controlled field trial of the Malaysian REDUCE programme Norliza. *Nutrition Journal*, *17*(74), 1–13. https://doi.org/10.1186/s12937-018-0379-1

Nowell, L. S., Norris, J. M., White, D. E., & Moules, N. J. (2017). Thematic analysis: Striving to meet the trustworthiness criteria. *International Journal of Qualitative Methods*, *16*(1), 1–13. https://doi.org/10.1177/1609406917733847

Nowicka, P., Sorjonen, K., Pietrobelli, A., Flodmark, C. E., & Faith, M. S. (2014). Parental feeding practices and associations with child weight status. Swedish validation of the Child Feeding Questionnaire finds parents of 4-year-olds less restrictive. *Appetite*, *81*, 232–241. https://doi.org/10.1016/j.appet.2014.06.027

Partap, U., Young, E. H., Allotey, P., Sandhu, M. S., & Reidpath, D. D. (2019). Characterisation and correlates of stunting among Malaysian children and adolescents aged 6-19 years. *Global Health, Epidemiology and Genomics*, *4*(May). https://doi.org/10.1017/gheg.2019.1

Pascale, R. T., Sternin, J., & Sternin, M. (2010). *The Power of Positive Deviance*. Harvard Business Press.

PDI. (2017). *All positive deviance projects*. Positive Deviance Initiative. https://positivedeviance.org/all-projects

People in Need. (2019). *Positive Deviance for Nutrition*. https://www.clovekvtisni.cz/media/publications/1309/file/positive-deviance-for-nutrition_people-in-need_october-2019.pdf

Phua, K. L. (2015). The health of Malaysia’s “Orang asli” peoples: A review of the scientific evidence on nutritional outcome, parasite infestations, and discussion on implications for clinical practice. *Malaysian Journal of Public Health Medicine*, *15*(1), 83–90.

Poh, B. K., Ng, B. K., Siti Haslinda, M. D., Nik Shanita, S., Wong, J. E., Budin, S. B., Ruzita, A. T., Ng, L. O., Khouw, I., & Norimah, A. K. (2013). Nutritional status and dietary intakes of children aged 6 months to 12 years: Findings of the Nutrition Survey of Malaysian Children (SEANUTS Malaysia). *British Journal of Nutrition*, *110*(Suppl 3), S21–S35. https://doi.org/10.1017/S0007114513002092

Prado, E. L., & Dewey, K. G. (2014). Nutrition and brain development in early life. *Nutrition Reviews*, *72*(4), 267–284. https://doi.org/10.1111/nure.12102

Pravana, N. K., Piryani, S., Chaurasiya, S. P., Kawan, R., Thapa, R. K., & Shrestha, S. (2017). Determinants of severe acute malnutrition among children under 5 years of age in Nepal: A community-based case-control study. *BMJ Open*, *7*, e017084. https://doi.org/10.1136/bmjopen-2017-017084

Psaki, S., Bhutta, Z. A., Ahmed, T., Ahmed, S., Bessong, P., Islam, M., John, S., Kosek, M., Lima, A., Nesamvuni, C., Shrestha, P., Svensen, E., McGrath, M., Richard, S., Seidman, J., Caulfield, L., Miller, M., & Checkley, W. (2012). Household food access and child malnutrition: Results from the eight-country MAL-ED study. *Population Health Metrics*, *10*, 1–11. https://doi.org/10.1186/1478-7954-10-24

Quah, P. L., Fries, L. R., Chan, M. J., Fogel, A., McCrickerd, K., Goh, A. T., Aris, I. M., Lee, Y. S., Pang, W. W., Basnyat, I., Wee, H. L., Yap, F., Godfrey, K. M., Chong, Y. S., Lynette, L. P., Tan, K. H., Forde, C. G., & Chong, M. F. F. (2019). Validation of the Children’s Eating Behavior Questionnaire in 5 and 6 year-old children: The GUSTO Cohort Study. *Frontiers in Psychology*, *10*, 824. https://doi.org/10.3389/fpsyg.2019.00824

Rachmi, C. N., Agho, K. E., Li, M., & Baur, L. A. (2016). Stunting, underweight and overweight in children aged 2.0-4.9 years in Indonesia: Prevalence trends and associated risk factors. *PLoS ONE*, *11*(5), e0154756. https://doi.org/10.1371/journal.pone.0154756

Ragini, Sengupta, P., & Benjamin, A. I. (2015). Impact assessment of nutritional education and motivation of mothers in food supplementation of malnourished 2-5 years old in an urban slum of Ludhiana: A field trial. *Indian Journal of Community Health*, *26*(Supp S2), 193–196.

Rannan-Eliya, R. P., Hossain, S. M., Anuranga, C., Wickramasinghe, R., Jayatissa, R., & Abeykoon, A. T. (2013). Trends and determinants of childhood stunting and underweight in Sri Lanka. *The Ceylon Medical Journal*, *58*(1), 10–18. https://doi.org/10.4038/cmj.v58i1.5357

Ravallion, M., Chen, S., & Sangraula, P. (2007). New evidence on the urbanization of global poverty. In *World Bank Policy Research Working Paper Series* (No. 4199). https://doi.org/10.1111/j.1728-4457.2007.00193.x

Reading, R., Harvey, I., & Mclean, M. (2000). Cluster randomised trials in maternal and child health: Implications for power and sample size. *Archives of Disease in Childhood*, *82*, 79–83. https://doi.org/10.1136/adc.82.1.79

Roche, M. L., Marquis, G. S., Gyorkos, T. W., Blouin, B., Sarsoza, J., & Kuhnlein, H. . (2017). A community-based Positive Deviance/Hearth infant and young child nutrition intervention in Ecuador improved diet and reduced underweight. *Journal of Nutrition Education and Behavior*, *49*(3), 196-203.e1. https://doi.org/10.1016/j.jneb.2016.10.007

Rojroongwasinkul, N., Kijboonchoo, K., Wimonpeerapattana, W., Purttiponthanee, S., Yamborisut, U., Boonpraderm, A., Kunapan, P., Thasanasuwan, W., & Khouw, I. (2013). SEANUTS: The nutritional status and dietary intakes of 0.5-12-year-old Thai children. *British Journal of Nutrition*, *110*(Suppl 3), S36–S44. https://doi.org/10.1017/S0007114513002110

Roy, S. K., Jolly, S. P., Shafique, S., Fuchs, G. J., Mahmud, Z., Chakraborty, B., & Roy, S. (2007). Prevention of malnutrition among young children in rural Bangladesh by a food-health-care educational intervention: A randomized controlled trial. *Food and Nutrition Bulletin*, *28*(4), 375–383. https://doi.org/10.1177/156482650702800401

Rytter, M. J. H., Kolte, L., Briend, A., Friis, H., & Christensen, V. B. (2014). The immune system in children with malnutrition - A systematic review. *PLoS ONE, 9*(8), e105017. https://doi.org/10.1371/journal.pone.0105017

Saaka, M., & Osman, S. M. (2013). Does household food insecurity affect the nutritional status of preschool children aged 6–36 months? *International Journal of Population Research*, *2013*, 304169. https://doi.org/10.1155/2013/304169

Sánchez-Encalada, S., Talavera-Torres, M. M., & Wong-Chew, R. M. (2019). An educational intervention to mothers improved the nutritional status of Mexican children younger than 5 years old with mild to moderate malnutrition. *Global Pediatric Health*, *6*, 1-9. https://doi.org/10.1177/2333794X19884827

Sandjaja, S., Budiman, B., Harahap, H., Ernawati, F., Soekatri, M., Widodo, Y., Sumedi, E., Rustan, E., Sofia, G., Syarief, S. N., & Khouw, I. (2013). Food consumption and nutritional and biochemical status of 0.5-12-year-old Indonesian children: The SEANUTS study. *British Journal of Nutrition*, *110*(Suppl 3), S11-S20. https://doi.org/10.1017/S0007114513002109

Santos Felisbino-Mendes, M., Villamor, E., & Velasquez-Melendez, G. (2014). Association

of maternal and child nutritional status in Brazil: A population based cross-sectional study. *PLoS ONE, 9*(1), e87486. https://doi.org/10.1371/journal.pone.0087486

Sarma, H., Khan, J. R., Asaduzzaman, M., Uddin, F., Tarannum, S., Hasan, M. M., Rahman, A. S., & Ahmed, T. (2017). Factors influencing the prevalence of stunting among children aged below five years in Bangladesh. *Food and Nutrition Bulletin, 38*(3), 291–301. https://doi.org/10.1177/0379572117710103

Schaafsma, A., Deurenberg, P., Calame, W., Van Den Heuvel, E. G. H. M., Van Beusekom, C., Hautvast, J., Sandjaja, S., Bee Koon, P., Rojroongwasinkul, N., Le Nguyen, B. K., Parikh, P., & Khouw, I. (2013). Design of the South East Asian Nutrition Survey (SEANUTS): A four-country multistage cluster design study. *British Journal of Nutrition, 110*(Suppl 3), S2–S10. https://doi.org/10.1017/S0007114513002067

Schoenmaker, C., Juffer, F., van IJzendoorn, M. H., van den Dries, L., Linting, M., van der Voort, A., & Bakermans-Kranenburg, M. J. (2015). Cognitive and health-related outcomes after exposure to early malnutrition: The Leiden longitudinal study of international adoptees. *Children and Youth Services Review, 48,* 80–86. https://doi.org/10.1016/j.childyouth.2014.12.010

Schooley, J., & Morales, L. (2007). Learning from the community to improve maternal – child health and nutrition : The positive deviance/hearth approach. *Journal of Midwifery & Women's Health, 52*(4), 376-83.https://doi.org/10.1016/j.jmwh.2007.03.001

Schoonees, A., Lombard, M., Musekiwa, A., Nel, E., & Volmink, J. (2019). Ready-to-use therapeutic food (RUTF) for home-based nutritional rehabilitation of severe acute malnutrition in children from six months to five years of age (Review). *Cochrane Database of Systematic Reviews, 5,* 1–184. https://doi.org/10.1002/14651858.CD009000.pub3.www.cochranelibrary.com

Selamat, R., Ahmad, H., Chong, Z. L., Zainuddin, A. A., Mohd Shariff, Z., & Abu Bakar, W. A. (2015). Household food insecurity in Malaysia: findings from Malaysian Adults Nutrition Survey. *Medical Journal of Malaysia*, *70*(S1), 11. http://www.e-mjm.org/2015/v70s1/mjm-sept-suppl-2079.html#:~:text=Results%3A The prevalence of Malaysian,%3A 11.5%25%2C 15.5%25).&text=It was significantly higher in,in Peninsular Malaysia (14.8%25).

Senbanjo, I. O., Olayiwola, I. O., Afolabi, W. A., & Senbanjo, O. C. (2013). Maternal and child under-nutrition in rural and urban communities of Lagos state, Nigeria: the relationship and risk factors. *BMC Research Notes*, *6*, 286. http://ovidsp.ovid.com/ovidweb.cgi?T=JS&PAGE=reference&D=emed11&NEWS=N&AN=23880121

Sethi, V., Sternin, M., Sharma, D., Bhanot, A., & Mebrahtu, S. (2017). Applying positive deviance for improving compliance to adolescent anemia control program in tribal communities of India. *Food and Nutrition Bulletin, 38*(3), 447–452. https://doi.org/10.1177/0379572117712791

Shahar, S., Lau, H., Sharifa, E. W. P., Amara, S., & Norizan, A. R. (2019). Health, access and nutritional issues among low-income population in Malaysia: Introductory note. *BMC Public Health*, *19*(Suppl 4), 552. https://doi.org/10.1186/s12889-019-6852-8

Shariff, Z. M., Khor, G. L., Sariman, S., Huang, S. L., Chin, Y. S., Yusof, B. N. M., Chan, Y. M., & Mohamad, M. (2015). The relationship between household income and dietary

intakes of 1-10 year old urban Malaysian. *Nutrition Research and Practice*, *9*(3), 278–287. https://doi.org/10.4162/nrp.2015.9.3.278

Sharma, N., Gupta, M., Aggarwal, A. K., & Gorle, M. (2020). Effectiveness of a culturally appropriate nutrition educational intervention delivered through health services to improve growth and complementary feeding of infants: A quasi-experimental study from Chandigarh, India. *PLoS ONE*, *15*(3), e0229755. https://doi.org/10.1371/journal.pone.0229755

Shetty, P. (2006). Malnutrition and undernutrition. *Medicine (United Kingdom)*, *34*(12), 524–529. https://doi.org/10.1016/j.mpmed.2018.12.012

Shewade, H. D., Patro, B. K., Bharti, B., Soundappan, K., Kaur, A., & Taneja, N. (2013). Effectiveness of indigenous ready-to-use therapeutic food in community-based management of uncomplicated severe acute malnutrition: A randomized controlled trial from India. *Journal of Tropical Pediatrics*, *59*(5), 393–398. https://doi.org/10.1093/tropej/fmt039

Shloim, N., Edelson, L. R., Martin, N., & Hetherington, M. M. (2015). Parenting styles, feeding styles, feeding practices, and weight status in 4-12 year-old children: A systematic review of the literature. *Frontiers in Psychology*, *6*, 1–20. https://doi.org/10.3389/fpsyg.2015.01849

Singh, A., Singh, A., & Ram, F. (2014). Household food insecurity and nutritional status of children and women in Nepal. *Food and Nutrition Bulletin*, *35*(1), 3–11. https://doi.org/10.1177/156482651403500101

Sinha, R. K., Dua, R., Bijalwan, V., Rohatgi, S., & Kumar, P. (2018). Determinants of stunting, wasting and underweight in five high-burden pockets of four Indian states. *Indian Journal of Community Medicine*, *43(4)*, 279–283. https://doi.org/10.4103/ijcm.IJCM

Siti Fatihah, M., Gan, W. Y., Norhasmah, S., Mohd Shariff, Z., & Ismail, S. I. F. (2019). Sociodemographic, nutritional, and environmental factors are associated with cognitive performance among Orang Asli children in Malaysia. *PLoS ONE*, *14*(7), e0219841. https://doi.org/10.1371/journal. pone.0219841

Sosanya, M. E., Adeosun, F. F., Okafor, D. T., & Ifitezue, L. C. (2018). Positive deviance—An expeditious tool for action to ameliorate malnutrition in resource-poor settings. *Journal of Nutritional Ecology and Food Research*, *4*(2), 178–187. https://doi.org/10.1166/jnef.2017.1165

Spill, M. K., Callahan, E. H., Shapiro, M. J., Spahn, J. M., Wong, Y. P., Benjamin-Neelon, S. E., Birch, L., Black, M. M., Cook, J. T., Faith, M. S., Mennella, J. A., & Casavale, K. O. (2019). Caregiver feeding practices and child weight outcomes: A systematic review. *American Journal of Clinical Nutrition*, *109*(Suppl), 990S-1002S. https://doi.org/10.1093/ajcn/nqy276

Spoede, E., Corkins, M. R., Spear, B. A., Becker, P. J., Gunnell Bellini, S., Hoy, M. K., Piemonte, T. A., & Rozga, M. (2020). Food insecurity and pediatric malnutrition related to under- and overweight in the United States: An evidence analysis center systematic review. *Journal of the Academy of Nutrition and Dietetics*. https://doi.org/10.1016/j.jand.2020.03.009

Springer, A., Nielsen, C., & Johansen, I. (2016). Positive Deviance by the Numbers. In *Positive Deviance Initiative*. https://positivedeviance.org/background/

Srivastava, A., Gwande, K., Bhattacharya, S., & Singh, V. K. (2019). Impact of the positive deviance approach on breastfeeding practices among tribal pregnant Women: A before – after intervention study. *CHRISMED Journal of Health and Research*, *6,* 222-228. https://doi.org/10.4103/cjhr.cjhr_165_18

Sternin, J. (2002). Positive deviance: A new paradigm for addressing today's problems today. *Journal of Corporate Citizenship* , *5*(Spring 2002), 57–62.

Sternin, M., Sternin, J., & Marsh, D. (1998). *Designing a community-based nutrition program using the hearth model and the positive deviance approach - A field guide*. Save the Children.

Subramanian, S. V., Ackerson, L. K., & Smith, G. D. (2010). Parental BMI and childhood undernutrition in India: An assessment of intrauterine influence. *Pediatrics*, *126*(3), e663-e671. https://doi.org/10.1542/peds.2010-0222

Sunuwar, D. R., Singh, D. R., & Pradhan, P. M. S. (2020). Prevalence and factors associated with double and triple burden of malnutrition among mothers and children in Nepal: Evidence from 2016 Nepal demographic and health survey. *BMC Public Health*, *20*, 405. https://doi.org/10.1186/s12889-020-8356-y

Tacoli, C., McGranahan, G., & Satterthwaite, D. (2015). Urbanisation, rural–urban migration and urban poverty. In *Background Paper for World Migration Report 2015 Migrants and Cities: New Urban Partnerships to Manage Mobility* (Vol. 10, Issue 19). http://pubs.iied.org/10725IIED

Tasnim, T. (2018). Determinants of malnutrition in children under five years in developing countries: A systematic review. *Indian Journal of Public Health Research and Development*, *9*(6), 333–338. https://doi.org/10.5958/0976-5506.2018.00574.0

Tebeje, N., Bikes, G., Abebe, S., & Yesuf, M. (2017). Prevalence and major contributors of child malnutrition in developing countries: Systematic review and meta-analysis. *Journal of Childhood Obesity*, *2*(4:16), 1–7. https://doi.org/10.21767/2572-5394.100037

Tee, E. S., Noor, M. I., Azudin, M. N., & Idris, K. (1997). *Nutrient Composition of Malaysian Foods*. Institute for Medical Research.

Tette, E. M. A., Sifah, E. K., & Nartey, E. T. (2015). Factors affecting malnutrition in children and the uptake of interventions to prevent the condition. *BMC Pediatrics*, *15*, 189. https://doi.org/10.1186/s12887-015-0496-3

The CORE group. (2002). *Positive deviance / Hearth essential elements: A resource guide for sustainably rehabilitating malnourished children*. CORE group.

The CORE group, USAID, & Counterpart India. (2003). *Positive Deviance and Hearth : Mechanisms for Community-Based Management of Malnutrition*.

The World Bank. (2018). *Poverty and Shared Prosperity 2018: Piecing together the poverty puzzle*. https://doi.org/10.1596/978-1-4648-1330-6

The World Bank group. (2018). *Improving nutrition outcomes for children in Sri Lanka's estate sector*.

Tiwari, R., Ausman, L. M., & Agho, K. E. (2014). Determinants of stunting and severe stunting among under-fives: Evidence from the 2011 Nepal Demographic and Health Survey. *BMC Pediatrics, 14,* 239. https://doi.org/10.1186/1471-2431-14-239

UNICEF. (2015). *UNICEF ' s approach to scaling up nutrition for mothers and their children*.

UNICEF. (2018). *Children without: A study of urban child poverty and deprivation in low-cost flats in Kuala Lumpur*. UNICEF Malaysia.

UNICEF. (2019). *The State of the World's Children 2019. Children, Food and Nutrition: Growing well in a changing world*. UNICEF.

UNICEF, WHO, & The World Bank. (2019). *Levels and trends in child malnutrition: key findings of the 2019 Edition of the Joint Child Malnutrition Estimates*. http://www.unicef.org/media/files/JME_2015_edition_Sept_2015.pdf

UNICEF, WHO, & The World Bank. (2020a). *Levels and trends in child malnutrition*. https://data.unicef.org/resources/jme-report-2020/

UNICEF, WHO, & The World Bank. (2020b). *Prevalence of underweight, weight for age (% of children under5)*. UNICEF, WHO, The World Bank: Joint Child Malnutrition Estimates (JME). https://databank.worldbank.org/reports.aspx?source=2&series=SH.STA.MALN.ZS&country=MYS,WLD

United Nations. (2015). *Transforming our world: The 2030 Agenda for sustainable development*.

United Nations. (2019). *World Urbanization Prospects: The 2018 Revision (ST/ESA/SER.A/420)*. https://doi.org/10.4054/demres.2005.12.9

USDA. (2012). *U.S. Household Food Security Survey Module: Six-Item Short Form*. https://www.ers.usda.gov/topics/food-nutrition-assistance/food-security-in-the-us/survey-tools/#six

USDA. (2019). *AMPM - USDA Automated Multiple-Pass Method*. https://www.ars.usda.gov/northeast-area/beltsville-md-bhnrc/beltsville-human-nutrition-research-center/food-surveys-research-group/docs/ampm-usda-automated-multiple-pass-method/

Vaitkevičiūtė, J., & Petrauskienė, A. (2019). The associations between body mass index of seven-and eight-year-old children, dietary behaviour and nutrition-related parenting practices. *Medicina*, *55*(1), 1–15. https://doi.org/10.3390/medicina55010024

Victora, C. G., Adair, L., Fall, C., Hallal, P. C., Martorell, R., Richter, L., & Sachdev, H. S. (2008). Maternal and child undernutrition: consequences for adult health and human capital. *The Lancet*, *371*, 340–357. https://doi.org/10.1016/S0140-6736(07)61692-4

Vossenaar, M., Mayorga, E., Soto-Méndez, M. J., Medina-Monchez, S. B., Campos, R., Anderson, A. S., & Solomons, N. W. (2009). The positive deviance approach can be used to create culturally appropriate eating guides compatible with reduced cancer risk. *The Journal of Nutrition*, *139*(4), 755–762. https://doi.org/10.3945/jn.108.100362

Waber, D. P., Bryce, C. P., Girard, J. M., Fischer, L. K., Fitzmaurice, G. M., & Galler, J. R. (2018). Parental history of moderate to severe infantile malnutrition is associated with

cognitive deficits in their adult offspring. *Nutr Neurosci*, *21*(3), 195–201. https://doi.org/10.1080/1028415X.2016.1258379

Wafa, S. W., Ruzita, A. T., Hamzaid, N. H., McColl, J. H., Rajikan, R., Ng, L. O., Ramli, A. H., & Reilly, J. J. (2011). Randomized controlled trial of a good practice approach to treatment of childhood obesity in Malaysia: Malaysian Childhood Obesity Treatment Trial (MASCOT). *International Journal of Pediatric Obesity*, *6*(2–2), 62–69. https://doi.org/10.3109/17477166.2011.566340

Wagh, V. D., & Deore, B. R. (2018). Ready to use therapeutic food (RUTF) formulation and packaging for malnutrition: An overview. *Advances in Life Sciences and Health*, *2*(1), 1–15. https://doi.org/10.19080/NFSIJ.2018.04.555648

Wamani, H., Åstrøm, A. N., Peterson, S., Tumwine, J. K., & Tylleskär, T. (2007). Boys are more stunted than girls in Sub-Saharan Africa: A meta-analysis of 16 demographic and health surveys. *BMC Pediatrics*, *7*, 17. https://doi.org/10.1186/1471-2431-7-17

Wan Manan, W. M., Jomo Kwame, S., & Tan, Z. G. (2019). *Addressing malnutrition in Malaysia* (License: Creative Commons Attribution CC BY 3.0.).

Wardle, J., Guthrie, C. A., Sanderson, S., & Rapoport, L. (2001). Development of the children's eating behaviour questionnaire. *Journal of Child Psychology and Psychiatry and Allied Disciplines*, *42*(7), 963–970. https://doi.org/10.1111/1469-7610.00792

Weber, J. M., Ryan, K. N., Tandon, R., Mathur, M., Girma, T., Steiner-Asiedu, M., Saalia, F., Zaidi, S., Soofi, S., Okos, M., Vosti, S. A., & Manary, M. J. (2017). Acceptability of locally produced ready-to-use therapeutic foods in Ethiopia, Ghana, Pakistan and India. *Maternal and Child Nutrition*, *13*(2), 1–9. https://doi.org/10.1111/mcn.12250

WHO. (2006). *WHO Child Growth Standards : Methods and development*. http://www.who.int/childgrowth/standards/Technical_report.pdf?ua=1

WHO. (2014). Global nutrition targets 2025: policy brief series (WHO/NMH/NHD/14.2). In *WHO*. https://doi.org/10.2165/00024677-200302060-00002

WHO. (2019). *WHO Anthro Survey Analyser Quick guide*.

Wishik, S. M., & Van Der Vynckt, S. (1976). The use of nutritional "positive deviants" to identify approaches for modification of dietary practices. *American Journal of Public Health*, *66*(1), 38–42. https://doi.org/10.2105/AJPH.66.1.38

Wisniewski, S. L. W. (2010). Child nutrition, health problems, and school achievement in Sri Lanka. *World Development*, *38*(3), 315–332. https://doi.org/10.1016/j.worlddev.2009.09.009

Wollinka, O., Keeley, E., Burkhalter, B. R., & Bashir, N. (1997). *Heart nutrition model: Applications in Haiti, Vietnam and Bangladesh*. Basic Support for Institutionalizing Child Survival (BASICS) Project. https://pdfs.semanticscholar.org/f5cd/3dc7daf83a82448f84bcf0ed909c8b1078de.pdf

Wong, C. Y., Mohd Shariff, Z., Adznam, S. N., Sulaiman, N., & Chin, Y. S. (2018). Weight and height faltering in the indigenous children (Orang Asli) of Peninsular Malaysia during the first 2 years of life. *Asia Pacific Journal of Clinical Nutrition*, *27*(4), 886–892. https://doi.org/10.6133/apjcn.072017.02

Wong, C. Y., Zalilah, M. S., Chua, E. Y., Norhasmah, S., Chin, Y. S., & Siti Nur'Asyura, A.

(2015). Double-burden of malnutrition among the indigenous peoples (Orang Asli) of Peninsular Malaysia. *BMC Public Health*, *15*, 680. https://doi.org/10.1186/s12889-015-2058-x

Wong, H. J., Moy, F. M., & Nair, S. (2014). Risk factors of malnutrition among preschool children in Terengganu, Malaysia: a case control study. *BMC Public Health*, *14*, 785. http://ovidsp.ovid.com/ovidweb.cgi?T=JS&PAGE=reference&D=emed13&NEWS=N&AN=25086853

World Bank, & International Monetary Fund (IMF). (2013). *Gloabal Monitoring Report 2013: Rural-Urban Dynamics and the Millennium Development Goals*. https://doi.org/10.1596/978-0-8213-9806-7

Yuan, J., Zhang, Y., Lu, Z., Xu, T., Zhang, H., Tan, Z., Yu, L., Jiang, X., & Shang, L. (2019). Correlation between children’s eating behaviors and caregivers’ feeding behaviors among preschool children in China. *Appetite*, *138*, 146–152. https://doi.org/10.1016/j.appet.2019.03.022

Yusop, N. B. M., Zalilah, M. S., Hwu, T. T., Abd Talib, R., & Spurrier, N. (2018). The effectiveness of a stage-based lifestyle modification intervention for obese children. *BMC Public Health*, *18*, 299. https://doi.org/10.1186/s12889-018-5206-2

Zaidi, Z., Jaffery, T., Shahid, A., Moin, S., Gilani, A., & Burdick, W. (2012). Change in action: Using positive deviance to improve student clinical performance. *Advances in Health Sciences Education*, *17*(1), 95–105. https://doi.org/10.1007/s10459-011-9301-8

Zakaria, L. N., Minhat, H. S., Nor Afiah, M. Z., Baharom, A., & Norliza, A. (2019). Factors contributing towards malnutrition among under five indigenous children: A systematic review. *Indian Journal of Science and Technology*, *12*(7), 1–12. https://doi.org/10.17485/ijst/2019/v12i7/141509

Zalilah, M.S, & Tham, B. . (2002). Food Security and Child Nutritional Status Among Orang Asli (Temuan) Households in Hulu langat, Selangor. *Med J Malaysia*, *57*(1), 36–50. http://www.e-mjm.org/2002/v57n1/Food_security.pdf

Zalilah, Mohd Shariff, Khor, G. L., Sariman, S., Chin, Y. S., Barakatun, N. M. Y., Chan, Y. M., Huang, S. L., & Maznorila, M. (2016). Higher dietary energy density is associated with stunting but not overweight and obesity in a sample of urban Malaysian children. *Ecology of Food and Nutrition*, *55*(4), 378–389. https://doi.org/10.1080/03670244.2016.1181065

Zeitlin, M., Ghassemi, H., & Mohamed Mansour. (1990). Positive deviance in child nutrition. *The Journal of Nervous and Mental Disease, 181*, 464. https://doi.org/10.1097/00005053-199307000-00023
